# Involving stakeholders in research priority setting: a scoping review

**DOI:** 10.1186/s40900-021-00318-6

**Published:** 2021-10-29

**Authors:** Christiane Grill

**Affiliations:** Ludwig Boltzmann Gesellschaft (LBG), Open Innovation in Science Center, Nussdorfer Strasse 64/2, 1090 Vienna, Austria

**Keywords:** Priority setting, Stakeholder involvement, Patient and public involvement, Research priorities, Scoping review

## Abstract

**Background:**

This scoping review provides a thorough analysis of how stakeholders have so far been involved in research priority setting. The review describes, synthesizes, and evaluates research priority setting projects not only for the field of health—as previous reviews have done—but does so on a much broader scale for any research area.

**Methods:**

A comprehensive electronic literature search was conducted in the databases PubMed, Scopus, and Web of Science. Reflecting the importance of grey literature, Google Scholar and relevant websites were also screened for eligible publications. A computational approach was then used for the study selection. The final screening for inclusion was done manually.

**Results:**

The scoping review encompasses 731 research priority setting projects published until the end of 2020. Overall, the projects were conducted within the realm of 50 subject areas ranging from agriculture and environment over health to social work and technology. Key learnings include that nearly all priority setting projects aimed to identify research priorities for the field of health (93%), particularly for nursing and care, cancer, pediatrics, and mental, behavioral and neurodevelopmental disorders. Only 6% of the projects were not health-related and 1% identified research priorities at the interface between health and a non-health area. Over time, 30 different stakeholder groups took part in research priority setting. The stakeholders most frequently asked to identify research priorities were doctors, patients, academics/researchers, nurses, allied healthcare professionals, family members, friends, and carers. Nearly two thirds of all projects have been conducted in Europe and North America. Overall, only 9% of the projects emphasized the importance of stakeholders in their goals and rationales and actively involved them. In around a quarter of the projects, stakeholders deliberated on their research priorities throughout the entire process.

**Conclusion:**

By mapping out the complex landscape of stakeholder involvement in research priority setting, this review guides future efforts to involve stakeholders effectively, inclusively, and transparently, which in turn may increase the overall value of research for society. As a practical addition to this review, the first worldwide research priority setting database was created: https://ois.lbg.ac.at/en/project-database. The database contains all the projects analyzed for this review and is constantly updated with the latest published research priority setting projects.

**Supplementary Information:**

The online version contains supplementary material available at 10.1186/s40900-021-00318-6.

## Plain English summary

Involving stakeholders already at the beginning of the research process when deciding what to research is called “research priority setting”. Research priority setting brings research closer to the needs and concerns of its stakeholders, particularly patients, family members, friends, carers and ordinary citizens. There is a general need to map out the complex landscape of stakeholder involvement in research priority setting. I found 731 projects that asked stakeholders to identify priorities for research. The projects were conducted along 50 different subject areas ranging from agriculture and environment over health to social work and technology. Most projects identified research priorities for nursing and care, cancer, pediatrics, and mental, behavioral and neurodevelopmental disorders. The stakeholders most frequently asked to identify research priorities were doctors, patients, academics/researchers, nurses, allied healthcare professionals, family members, friends, and carers. Overall, half of all projects explicitly mentioned that involving stakeholders is important. Around one quarter of all projects also actively involved patients and the public. In around one quarter of all projects, stakeholders deliberated on their priorities for research throughout the entire process. As researchers are still skeptical towards the benefits of involving stakeholders in research priority setting, future research on this matter is greatly needed.

**Supplementary Information:**

The online version contains supplementary material available at 10.1186/s40900-021-00318-6.

## Introduction

Traditionally, researchers, research institutions or funding organizations decide on the questions that research should answer. The corporate world, however, has demonstrated very early on that involving stakeholders in defining research and development (R&D) activities can be very beneficial [3]. Many of the best ideas for new products and services (e.g., LEGO sets, Local Motors’ cars, or telecommunication applications for Orange) have originated from stakeholders having a say in setting the R&D agenda [4, 5]. A gradual turn of tide can also be observed in science. Influential bodies, like the European Commission (EC) [6], the Organisation for Economic Co-Operation and Development (OECD) [7], and the World Health Organization (WHO) [8], or UK’s National Institute for Health Research (NIHR) [9] are strongly advising researchers to actively involve non-research stakeholders in setting the scientific research agenda. And indeed, increasing efforts are made to identify stakeholders’ research needs by involving them in “research priority setting”.

Research priority setting^1^ encompasses any activities that involve stakeholders in identifying, prioritizing, and reaching consensus on those areas, topics, or questions that research needs to address [10, 11]. Particularly in the first stage of the research process, when deciding what to research, input by non-research stakeholders can be very beneficial. It has been shown to promote the uptake and implementation of research evidence [12], secure optimal return on investment [13], reduce “research waste” [14], and foster the relevance and legitimacy of research overall [10].

To date, several scoping reviews on research priority setting exist. These reviews have all aimed at systematically compiling, analyzing, and evaluating research priority setting for the field of health. Some reviews have done so for specific health topics [12, 15–20]. Others have looked at health research priority setting conducted in specific geographical areas [21–24], during specific time periods [25–27], or fulfilling a mix of parameters [28, 29]. And lastly, other studies have reviewed specific design characteristics of health research priority setting [30–34].

What is yet missing, however, is a thorough analysis of how non-research stakeholders have so far been involved in research priority setting. This review, thus, sets out to describe, synthesize, and evaluate research priority setting projects not only for the field of health—as previous reviews have done—but does so on a much broader scale for any research field worldwide. The review questions touch three broad areas of interest: (1) the general characteristics of research priority setting projects with stakeholder involvement, (2) the importance of stakeholder involvement, and (3) the methods and approaches to involve stakeholders in research priority setting. The specific questions guiding the review are: (1) What are the general characteristics of those research priority setting projects that involved stakeholders to set the research agenda? More precisely: (1.1) For which subject areas are stakeholders involved in setting research priorities? (1.2) Which stakeholder groups are involved in research priority setting? (1.3) In which countries are stakeholders involved in research priority setting? (2) How much importance do the priority setting projects attribute to stakeholder involvement? (2.1) Is stakeholder involvement named as an explicit goal? (2.2) Is stakeholder involvement named as a reason for conducting research priority setting? (2.3) Are stakeholders included in governance structures (i.e., steering groups, advisory boards)? (2.4) On what level is the public involved in research priority setting? (3) How are stakeholders’ research priorities elicited? More precisely: (3.1) What methods are applied to elicit stakeholders’ research priorities? (3.2) What are the specific approaches to elicit stakeholders’ research priorities?

Mapping out the complex landscape of stakeholder involvement in research priority setting may ultimately guide future efforts to involve stakeholders effectively, inclusively, and transparently, which in turn may increase the overall value of research for society.

## Methods

A study protocol was first developed to describe the rationale and planned course of action of the review [35].

### Selection criteria

Studies that reported how non-research stakeholders were involved in setting priorities for research and published by the end of 2020 in English were included. Studies in which only researchers were involved in setting priorities for research were excluded. Furthermore, studies assessing priorities for practice and policy, non-research articles (e.g., policy documents, clinical guidelines, editorials, commentaries), and articles that did not include information about stakeholders and methods were excluded.

### Search strategy

A comprehensive electronic literature search was conducted from June to July 2020. To minimize any possible biases, several sources were searched from their inception to June/July 2020. Additionally, the searches were updated in January 2021 to include all research priority setting projects published by the end of 2020.

Due to the many synonyms for priority setting, a broad search approach was applied. Thus, the following search strings were defined: “priority setting”, “research priorit*”, “priority research”, “research agenda setting”, “agenda setting + research”, “agenda setting + priorit*”, “research agenda + priorit*”, “resource allocation + priorit*”, “allocation of resources + priorit*”, and “rationing + priorit*”.

The literature databases PubMed, Scopus, and Web of Science were searched for these search strings in title or abstract. The exact search strings for each database can be found in Additional file 1. The hits with all available information (authors, title, abstract, publication year, publication outlet, the digital object identifier [DOI; i.e., the persistent identifier or handle used to identify objects]), publication type, keywords, download link) were saved in comma separated values (csv) files.

Reflecting the importance of grey literature, title searches were conducted in Google Scholar for the same search strings (see Additional file 1). Using the free software environment for statistical computing and graphics “*R*” and the freely available, web scraping R-package “rvest” [36] all hits (excluding patents and citations) with all available information (authors, title, abstract, publication year, download link) were saved in csv files. Since most of the grey literature begins to appear on Google Scholar after approximately 20 to 30 pages of results [37], it was decided to include all hits on all pages. However, regardless of the number of hits found, Google Scholar only allows to extract hits until page 99 totaling 990 hits as a maximum. Additionally, the websites of organizations that are internationally known for advising and conducting research priority setting were searched for publications. More precisely, any publications as well as final reports of priority setting partnerships published on the website of the James Lind Alliance [38, 39], publications on the website of the Cochrane Priority Setting Methods Group [40], and the website of the WHO priority setting methods [40] were downloaded automatically using “rvest”. All publications with all available information (authors, title, abstract, publication year, download link) were saved in csv files.

### Study selection

A computational approach using R was used for all study selection steps except the last one—the final screening for inclusion. First, the csv files from all searches were merged into one long list of hits (i.e., studies). Second, duplicates identified based on the DOI, or the exact title were computationally removed. Also, it was checked whether the language of title and abstract were indeed English by using the freely available R-package “cld3: Google's Compact Language Detector 3” [41]. Any non-English hits were removed. Additionally, any non-research articles were deleted from the hit list. In other words, all sorts of reviews (e.g., literature reviews, meta-analyses, article reviews, book reviews), opinion letters and personal narratives, clinical trial reports, and guidelines were deleted from the hit list. Criterion was the specification of the publication type as indicated in the databases PubMed, Scopus, and Web of Science.

These steps resulted in a longlist of hits that needed to be screened for eligibility. Some of these hits did not have an abstract. Since eligibility cannot be decided only based on the title, these hits were then manually screened for eligibility by looking them up. All hits that contained a title and an abstract were further screened for eligibility by calculating a structural topic model (STM).

The STM is a type of statistical modeling that aims to detect overarching, latent topics in documents^2^ based on the words that occur in these documents via a bag-of-words approach [42]. The STM thereby assumes that each document contains a mixture over topics, and each topic contains a mixture over words. The STM calculates for each hit the probability that the hit pertains to a specific topic (i.e., topic probabilities) and for each word the probability that the word belongs to a specific topic (i.e., word probabilities). Overall, the STM found 52 overarching, latent topics. Additional file 2 reports the top 15 words that most probably belong to each of the 52 topics. Human interpretation is then needed to evaluate the results and draw conclusions. In a first step, two human coders reflected on the top 15 words that most probably belong to each topic. This human interpretation led to the finding that the top words for topic number 1 and topic number 5 best reflect the concept of research priority setting.^3^ In a second step, ten randomly selected hits that most probably belong to each topic were extracted and two human coders evaluated the extent to which the extracted hits are studies on research priority setting. This human interpretation led to the conclusion that indeed topic number 1 and topic number 5 best reflect the concept of research priority setting. Subsequently, all hits that had a probability of more than 10% that they belonged to either of the two topics (hereby following the recommendation [42]) moved forward on the shortlist of studies to be checked manually for inclusion. Title and abstract of all eligible studies were checked manually whether they fulfilled the selection criteria. If so, the full texts were obtained and validated a final time against the selection criteria. Validity and reliability of the computational approach was tested thoroughly throughout the entire process.

### Data extraction and synthesis

A data extraction form was developed specifically for this review and piloted on a small sample of randomly selected studies (n = 25). For all included studies, the following information was extracted into a csv file. As to the general characteristics of research priority setting projects with stakeholder involvement, the project’s subject area, involved countries, and the study’s publication year were extracted. To measure the importance that the projects attribute to stakeholder involvement, the project’s goal, the reasons for conducting research priority setting, details on the governance structure (i.e., steering groups, advisory boards), and information on the level of public involvement in these projects were extracted. As to the procedure to elicit stakeholders’ research priorities, the specific methods and approaches to do so were extracted. In a subsequent step, the extracted information was manually coded (i.e., classified along broader categories) (see Additional file 3), and in a last step due to the large amount of data quantitatively analyzed.

## Results

### Search results

**Fig. 1:**
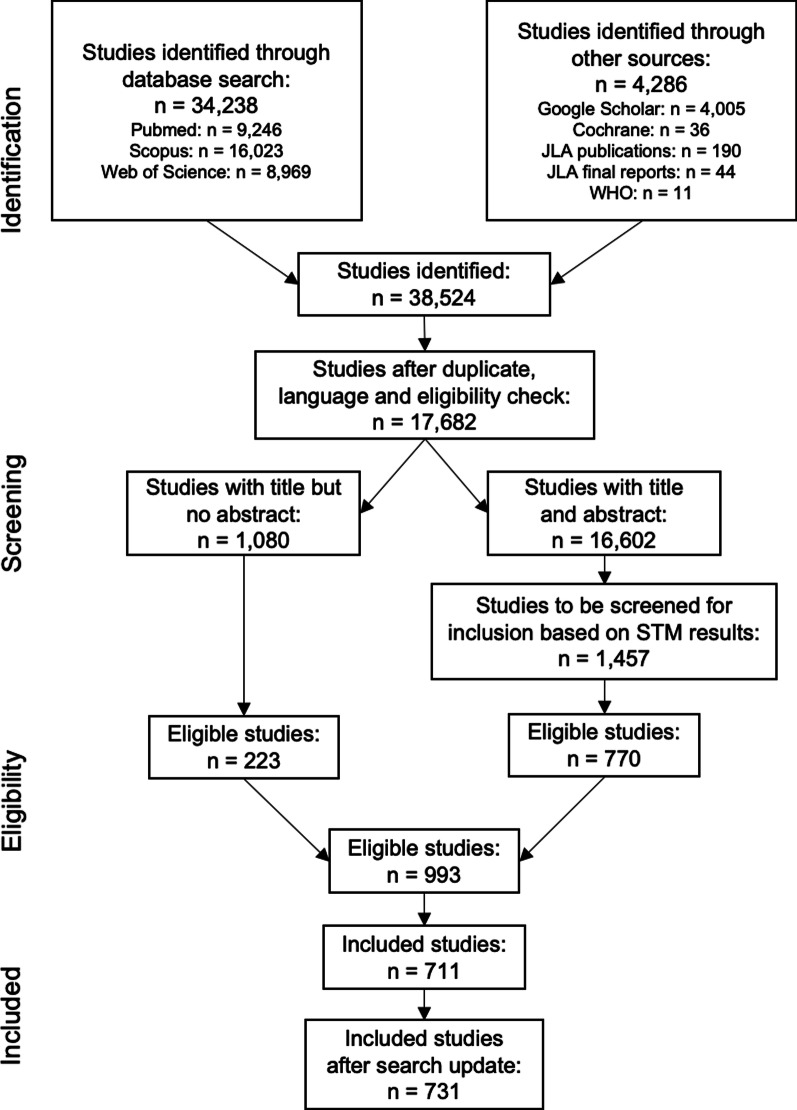
PRISMA Flowchart of Study Selection Process

Altogether, the literature search identified 38,524 studies. After removing duplicates, checking for English as the publication language, and excluding any non-research articles, 17,682 studies (45.9%) remained. Manually screening the 1,080 studies with a title but not an abstract for eligibility resulted in 223 studies. Running the STM on the 16,602 studies with a title and an abstract resulted in 1,457 studies that had a topic probability of > 0.1 for either of the two topics. The title and abstracts of these studies were then manually screened and resulted in 770 eligible studies. Overall, 993 (2.6%) studies were eligible for inclusion. After a final round of screening the full texts of the studies, 711 studies were then included in the scoping review. An additional 20 studies were added in January 2021 due to a literature search update. Altogether, the scoping review now contains 731 priority setting projects in which stakeholders were involved in identifying priorities for research. The study selection process is depicted in the PRISMA flowchart (Fig. 1).

### General characteristics of research priority setting with stakeholder involvement

**Fig. 2:**
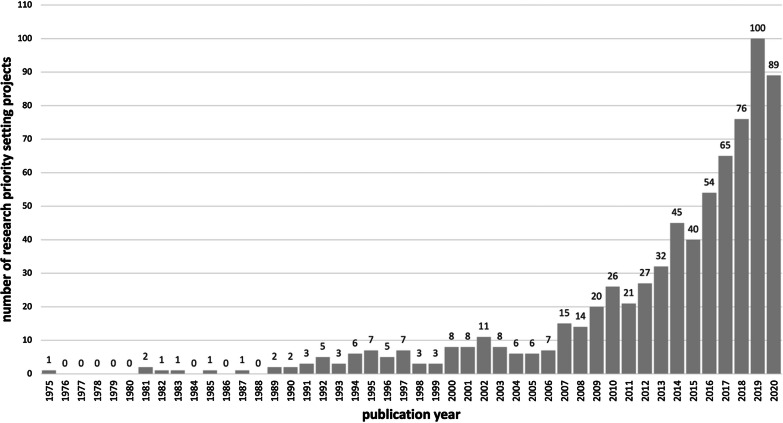
Frequency Distribution of Research Priority Setting Projects over Time

The scoping review encompasses 731 research priority setting projects that involved stakeholders and were published until the end of 2020 [see Additional file 4]. Figure 2 shows the frequency distribution of research priority setting projects over time. The first research priority setting that involved stakeholders was published in 1975 and is titled “Delphi Survey of Priorities in Clinical Nursing Research” by Carol A. Lindeman [43]. Until the mid 90’s, research priority setting projects were isolated occurrences. Since the beginning of the 2000s, the number of published projects has grown steadily with a particular large increase since 2007. The largest number of published research priority setting projects can be found for the years 2019 (n = 100) and 2020 (n = 89).

#### Subject areas

Overall, the research priority setting projects in which stakeholders were involved were conducted within the realm of 12 subject areas ranging from agriculture and environment over health to social work and technology. Due to the large number of projects within the field of health, this subject area was further divided into 38 areas along the international classification of diseases “ICD-11 for Mortality and Morbidity Statistics” provided by WHO [44] resulting in a list of 50 subject areas.

Overall, 77% of the projects related to one specific subject area, 22% of the projects to two different subject areas, and 10 projects (1%) even encompassed three subject areas. For instance, the research priority setting project “Educational Research Priorities for Pediatric Physical Therapy: A Consensus Study” by Moerchen et al. [45] was assigned to the areas: health education, pediatrics, and physical mobility and fitness; or, the project “Prioritizing Research for Integrated Implementation of Early Childhood Development and Maternal, Newborn, Child and Adolescent Health and Nutrition Platforms” by Sharma et al. [46] was assigned to the areas: nutrition, pregnancy and childbirth, and pediatrics.

Nearly all priority setting projects aimed to identify research priorities for the field of health: Of the 731 projects, 93% (n = 683) were conducted exclusively for the subject area of health and only 6% (n = 40) were not health-related at all. 1% (n = 8) of the priority setting projects identified research priorities at the interface between health and a non-health area.

Of the 48 non-health subject areas, the most common areas (see Table 1) were environment (38%, n = 18), social work (13%, n = 6), and technology (10%, n = 5). Other non-health areas for which stakeholders were asked to identify research priorities were agriculture (8%, n = 4), communication (6%, n = 3), development (6%, n = 3), education (6%, n = 3), law (4%, n = 2), citizen science (2%, n = 1), construction (2%, n = 1), human resources (2%, n = 1), and logistics (2%, n = 1).

**Table 1:** Frequency table of priority setting projects with a non-health subject area

	n	%
Environment	18	38
Social work	6	13
Technology	5	10
Agriculture	4	8
Communication	3	6
Development	3	6
Education	3	6
Law	2	4
Citizen science	1	2
Construction	1	2
Human resources	1	2
Logistics	1	2
	48	100

Frequencies are sorted in descending order by the number of projects

If non-research stakeholders were involved in setting health research priorities, they were most likely asked to do so for the area nursing and care (26%, n = 178; see Table 2). Other areas, for which stakeholders were frequently asked to set research priorities, were cancer (10%, n = 71), pediatrics (10%, n = 71), and mental, behavioral and neurodevelopmental disorders (10%, n = 65). Several projects focused on public health (6%, n = 41), pregnancy and childbirth (6%, n = 40), infectious and parasitic diseases (4%, n = 30), surgery (4%, n = 27), the nervous system (4%, n = 26), physical mobility and fitness (4%, n = 25), endocrine, nutritional and metabolic diseases (4%, n = 23), and injuries (4%, n = 23). And a few projects identified research priorities for emergency medicine (3%, n = 19), health research (3%, n = 18), the circulatory system (2%, n = 17), nutrition (2%, n = 17), the genitourinary system (2%, n = 15), the musculoskeletal system and connective tissue (2%, n = 15), aging (2%, n = 14), health and patient safety (2%, n = 14), the skin (2%, n = 13), health education (2%, n = 12), the health system (2%, n = 12), the respiratory system (2%, n = 11), the digestive system (1%, n = 10), general symptoms, signs and clinical findings (1%, n = 7), substance use and addictive behavior (1%, n = 7), sexual health (< 1%, n = 6), dentistry (< 1%, n = 5), organs and tissues (< 1%, n = 5), developmental anomalies (< 1%, n = 4), health communication (< 1%, n = 4), animal health (< 1%, n = 3), blood (< 1%, n = 3), complementary medicine (< 1%, n = 3), the ear (< 1%, n = 3), the visual system (< 1%, n = 3), and digital health (< 1%, n = 1).

**Table 2:** Frequency table of priority setting projects with a health subject area

	n	
Nursing and care	178	26
Cancer	71	10
Pediatrics	71	10
Mental, behavioral and neurodevelopmental disorders	65	10
Public health	41	6
Pregnancy and childbirth	40	6
Infectious and parasitic diseases	30	4
Surgery	27	4
Nervous system	26	4
Physical mobility and fitness	25	4
Endocrine, nutritional and metabolic diseases	23	3
Injuries	23	3
Emergency medicine	19	3
Health research	18	3
Circulatory system	17	2
Nutrition	17	2
Genitourinary system	15	2
Musculoskeletal system and connective tissue	15	2
Aging	14	2
Health and patient safety	14	2
Skin	13	2
Health education	12	2
Health system	12	2
Respiratory system	11	2
Digestive system	10	1
General symptoms, signs and clinical findings	7	1
Substance use and addictive behaviors	7	1
Sexual health	6	1
Dentistry	5	1
Organs and tissues	5	1
Developmental anomalies	4	1
Health communication	4	1
Animal health	3	0.4
Blood	3	0.4
Complementary medicine	3	0.4
Ear	3	0.4
Visual system	3	0.4
Digital health	1	0.1
	691	100

Frequencies are sorted in descending order by the number of projects

**Fig. 3:**
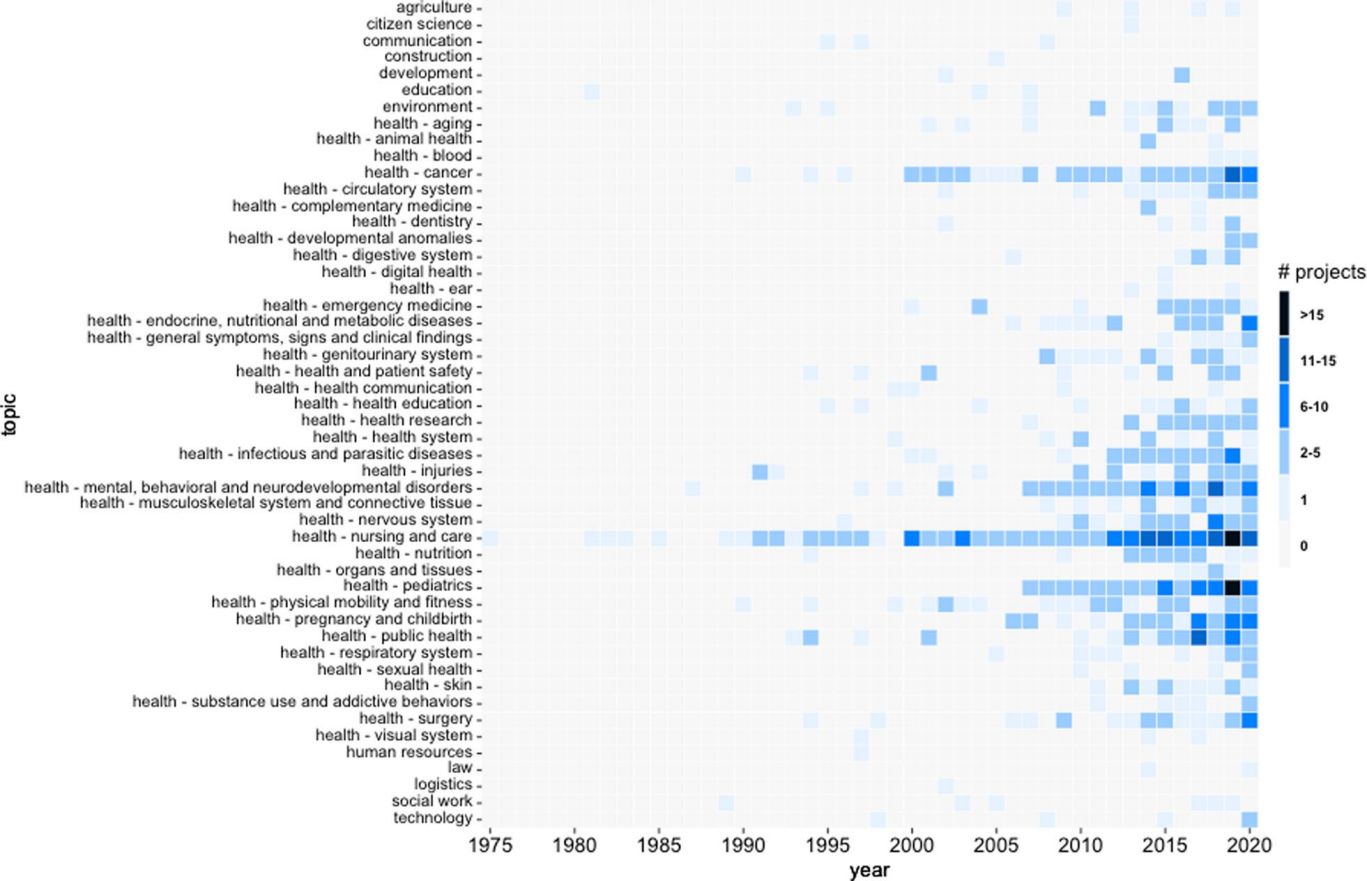
Heatmap of Subject Areas over Time

Figure 3 shows a heatmap of the subject areas over time. Specifically, the figure depicts how many research priority setting projects on each subject area with stakeholder involvement were conducted for each year ranging from 1975 to 2020. As can be seen, research priority setting projects relating to nursing and care were nearly continuously conducted since 1975. With the rise of research priority setting projects overall over time, also the number of projects aiming to identify research priorities for nursing and care increased. Furthermore, priority setting projects on cancer were frequently conducted since 1999 with a particularly large increase of conducted projects in the years 2019 and 2020. Two areas that have been trending since 2007 and especially in the last five years are pediatrics, and mental, behavioral and neurodevelopmental disorders. The most recent, trending subject areas of the last few years for which stakeholders were involved in research priority setting were infectious and parasitic diseases, public health, and pregnancy and childbirth. Even though stakeholders have been involved in a wide variety of research priority setting projects, Fig. 3 also shows that stakeholder involvement in research priority setting is unevenly distributed. While for a few subject areas many priority setting projects with stakeholder involvement have been carried out, for many other areas stakeholders have thus far barely been involved in setting the research agenda.

#### Stakeholder groups

Overall, 30 different stakeholder groups became involved in 731 research priority setting projects. The stakeholders most frequently asked to identify research priorities were doctors (43% of all projects, n = 316; see Table 3), patients (43%, n = 315), academics/researchers (41%, n = 302), nurses (37%, n = 269), allied healthcare professionals (37%, n = 267), family members/friends/carers (27%, n = 200), policymakers (15%, n = 110), healthcare providers without further specification (9%, n = 66), non-governmental organizations (9%, n = 65), governmental agencies (7%, n = 54), funders (6%, n = 42), and educators (5%, n = 39). Stakeholders infrequently involved in research priority setting were citizens (2%, n = 14), industry (2%, n = 12), learners (1%, n = 10), spiritual service providers (1%, n = 9), environmental practitioners (1%, n = 8), social workers (1%, n = 7), communication practitioners (< 1%, n = 4), agriculturists (< 1%, n = 3), business professionals (< 1%, n = 3), defense service providers (< 1%, n = 3), technology practitioners (< 1%, n = 3), development practitioners (< 1%, n = 2), veterinarian healthcare providers (< 1%, n = 2), users/consumers (< 1%, n = 2), the construction sector (< 1%, n = 1), financial service providers (< 1%, n = 1), the labor union (< 1%, n = 1), and legal service providers (< 1%, n = 1).

**Table 3:** Frequency table of involved stakeholder groups

	n	%
Doctors	316	43
Patients	315	43
Academics/researchers	302	41
Nurses	269	37
Allied healthcare professionals	267	37
Family/friends/carers	200	27
Policymakers	110	15
Healthcare providers	66	9
NGOs	65	9
Agencies	54	7
Funders	42	6
Educators	39	5
Citizens	14	2
Industry	12	2
Learners	10	1
Spiritual service providers	9	1
Environmental practitioners	8	1
Social workers	7	1
Communication practitioners	4	1
Agriculturists	3	0
Business professionals	3	0
Defense service providers	3	0
Technology practitioners	3	0
Development practitioners	2	0
Veterinarian healthcare providers	2	0
Users/consumers	2	0
Construction sector	1	0
Financial service providers	1	0
Labor union	1	0
Legal service providers	1	0
	2131	100

Frequencies are sorted in descending order by the number of projects

The overall number of different stakeholder groups involved in one research priority setting project ranged from one to nine. 29% of the projects involved one stakeholder group, followed by 19% involving three different groups, 18% four groups, 15% two groups and 13% five groups. On average, 2.9 stakeholder groups per project aimed to identify research priorities.

**Fig. 4:**
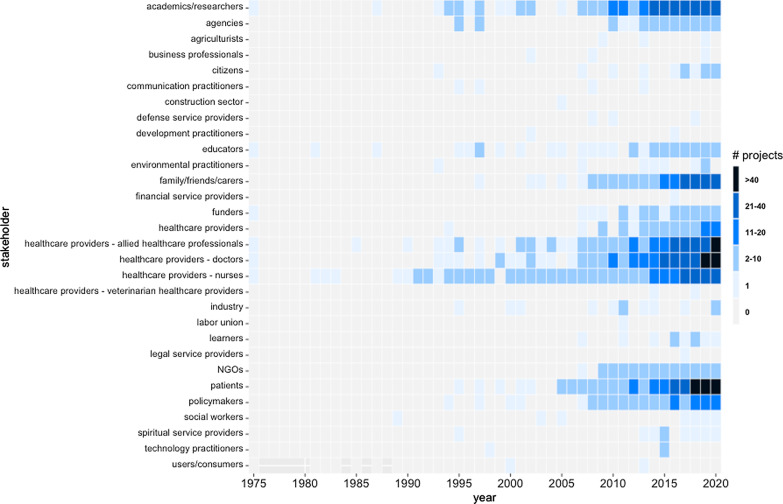
Heatmap of Involved Stakeholder Groups over Time

Figure 4 presents a heatmap of the stakeholder groups involved in research priority setting projects over time. Since health was the major research area for which priority setting projects were conducted over time, it is not surprising that healthcare providers were particularly frequently present as stakeholders over time. Other subject area-specific professions were infrequently involved. The stakeholder group that was most strongly involved in research priority setting over time were nurses. Since 2005, doctors, patients, family members/friends/carers as well as academics/researchers have more and more become involved in these projects. Looking at the last five years, the most indispensable stakeholders, who were most frequently asked to identify research priorities, were doctors, patients, and allied healthcare professionals, followed by family members/friends/carers, nurses, policymakers, and academics/researchers.

#### Countries

**Fig. 5:**
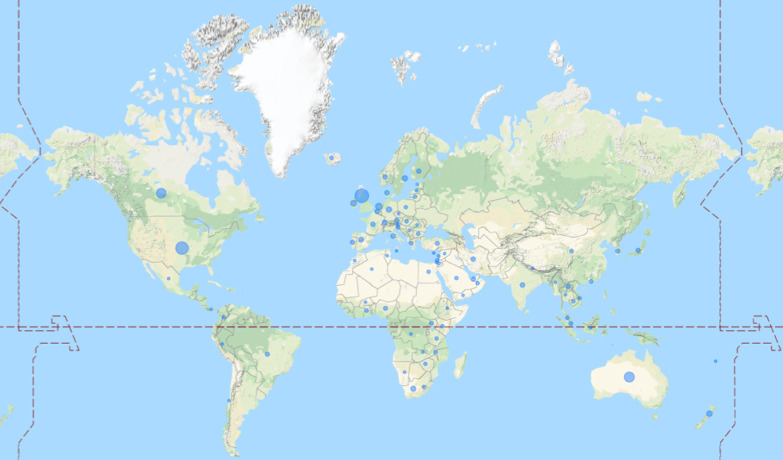
Countries of Research Priority Setting Projects. Note: The larger the blue circle, the more projects have been realized in that country. Projects that have been conducted internationally or in whole continents were excluded in this figure.

Figure 5 visualizes the countries in which research priority setting projects with stakeholder involvement were conducted. Of the 731 projects identified nearly two thirds have been conducted in Europe (38%) and North America (26%). 11% of the projects were conducted in Australia, 7% in Asia, 5% in Africa and 1% in South America. 12% of the projects were conducted internationally without any further geographic specification. The top 10 countries in which most research priority setting projects were located are the UK (21%, n = 199), the USA (17%, n = 159), Australia (9%, n = 88), Canada (9%, n = 80), the Netherlands (2%, n = 23), Ireland (2%, n = 16), New Zealand (2%, n = 14), Germany (1%, n = 13), Sweden (1%, n = 13), and South Africa (1%, n = 10).

**Fig. 6:**
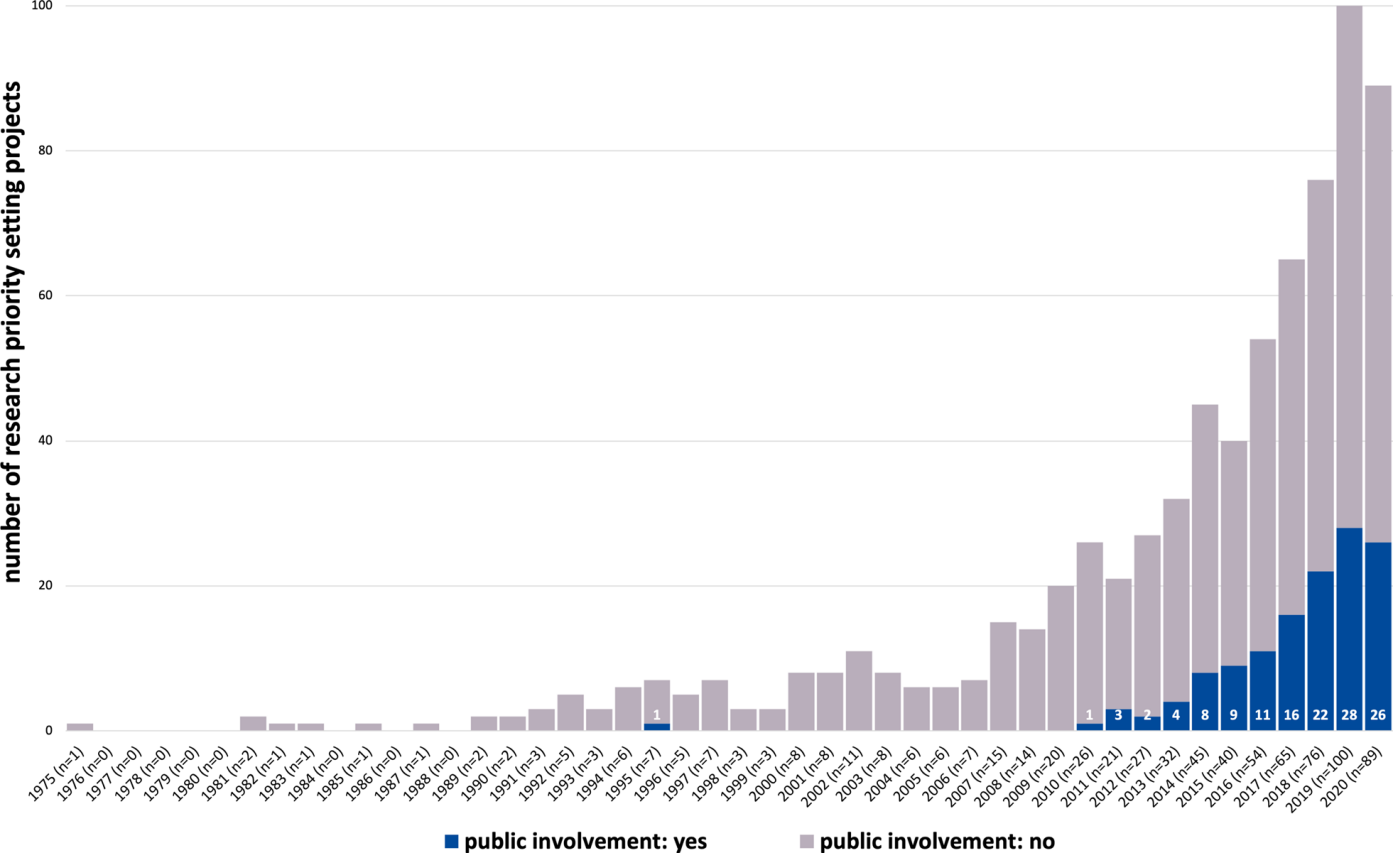
Frequency Distribution of Patient and Public Involvement over Time

### The importance of stakeholder involvement

This section analyzes how much importance research priority setting projects attributed to stakeholder involvement.

#### Goal

Regarding the question whether stakeholder involvement is named as an explicit goal, the analysis shows that 56% (n = 408) of the projects explicitly stated as their goal the involvement of stakeholders. For instance, these projects aimed “*to identify and prioritize research questions with greatest value to emergency nurses and of highest importance for health care consumers*” [47], “*to engage caregivers, clinicians, researchers, and managers to identify priority topics for caregiver research in cancer care*” [48], or “*to bring together people who smoke or vape, people who do not smoke and healthcare professionals to identify and agree on priorities for electronic cigarette research in the UK*” [49]. On the other hand, an equally large number of projects (44%, n = 323) did not mention stakeholder involvement as an explicit goal. For instance, their goals were “*to establish priorities for research in critical care medicine in the UK*” [50], “*to develop a national, prioritized research agenda for advance care planning*” [51], or “*to identify important areas for future osteoporosis research*” [52].

#### Rationale

Moreover, the question whether stakeholder involvement was named as a reason for conducting research priority setting was explored. Overall, the following reasons—individually or in combination with other reasons—were given: general need for research (e.g., because a field is under-researched [53], 48%, n = 353), no knowledge of stakeholders’ research priorities (e.g., questions of importance to patients and their families are yet unknown [54], 46%, n = 335), disease burden (e.g., arguing how many people are suffering from a specific disease [55], 10%, n = 71), impact (e.g., research on the defined priorities positively affects the lives of those concerned [56], 9%, n = 68), and more effective use of scarce resources (e.g., scare resources make it necessary to prioritize research foci [57], 9%, n = 65). As the analysis reveals, nearly half of all identified research priority setting projects named stakeholder involvement as an explicit reason for conducting research priority setting.

#### Governance

This section now answers the question whether stakeholders were included in the governance structures (i.e., steering groups, advisory boards) of research priority setting projects. Overall, if projects had steering groups or advisory boards, stakeholders were members of them; with one exception: the research priority setting project by Chamberlain et al. [58] invited only researchers to become members of the steering group. Overall, 23% (n = 168) of the projects involved stakeholders in their governance structure while more than three quarters (77%, n = 563) did not even have a governance structure at all (including the exception of Chamberlain et al.). Of the 168 projects with stakeholder involvement at the governance level, the vast majority (81%, n = 137) had a steering group, 14% (n = 13) an advisory board, and 5% (n = 8) both a steering group and an advisory board.

#### Level of public involvement

This section analyzes on what level the public was involved in research priority setting. In line with the definition of UK's public participation charity INVOLVE [59], the term “public” includes patients, potential patients, carers, people who use services, and people from organizations that represent people who use services. “Public involvement” in research is defined as research being carried out with or by member of the public rather to, about or for them. Involvement is hereby distinguished from participation and engagement. Involvement means active involvement in research projects; for instance, as grant holders, members of advisory and steering groups, co-developing materials, carrying out research. In contrast, participation means that the public takes part in research studies; for instance, they complete questionnaires or participate in interviews and focus groups. Engagement means that information and knowledge about research is provided and disseminated to the public; for instance via science festivals or through the media. The analysis revealed that in 52% (n = 384) of the identified projects the public neither participated nor was actively involved in research priority setting. In 30% (n = 216) of the projects, patients, family members/friends/carers or citizens participated in research priority setting: they took part in surveys, interviews, focus groups and so forth. In 17% (n = 126) of the projects, the public not only took part in the research studies but was also actively involved in the project as members of advisory boards or steering groups, or by co-developing materials and so forth. In 1% (n = 5) of the projects, the public was actively involved—more precisely, they were members of advisory boards and steering groups—but did not take part in the research studies as participants.

Looking at the development of public participation and involvement in research priority setting over time, the analysis provides a more granular picture. It was not until 1993 that the public took part in research priority setting for the first time. Specifically, the project “Explorations in Consultation of the Public and Health Professionals on Priority Setting in an Inner London Health District” by Bowling et al. [60] asked members of a local community to fill in a survey. Two years later, in 1995, the public was for the first time actively involved in research priority setting. In the project “Setting priorities for research and development in the NHS: a case study on the interface between primary and secondary care” by Jones et al. [61] consumers of health services were members of the advisory group. As Fig. 6 shows, the public was not actively involved again in research priority setting until 2010. Since 2010, however, active involvement of the public has constantly been growing. Particularly in the last three years (2018–2020), around one third of all annually published research priority setting projects actively involved the public.

#### Overall stakeholder importance

Lastly, the analysis looks at the overall importance that research priority setting projects attribute to stakeholder involvement. One quarter (26%, n = 192) of all projects did not attribute importance to stakeholder involvement at all. These projects did not name stakeholder involvement as an explicit goal or a reason for conducting the project and did not actively involved stakeholders in the research study. The rest of the projects attributed importance to stakeholder involvement in varying degrees. 9% (n = 67) of the identified projects explicitly mentioned stakeholder involvement as a goal and a reason for conducting the project, and actively involved stakeholders. Around one quarter of all projects (23%, n = 168) mentioned stakeholder involvement in their goals and rationales, 16% (n = 116) did so in their goals, and 12% (n = 85) in their reasons, but none of these projects actively involved stakeholders. In other words, stakeholders were mere participants of the research study. The remaining projects actively involved stakeholders—especially as members of steering groups and advisory boards—and sometimes additionally named stakeholder involvement as an explicit goal or reason for the project (governance: 4%, n = 31, goal and governance: 8%, n = 57, reason and governance: 2%, n = 15).

When looking at stakeholder importance over time, the analysis reveals that particularly during the last ten years stakeholders have become more and more important in setting research priorities. Continuously naming stakeholder involvement an explicit goal started in 2002 and since 2013 at least half of all research priority setting projects were doing so. Before that only scattered mentions of stakeholder involvement were made. The first research priority setting project that specifically set out to involve stakeholders (i.e., patients) was the study by Lewandowski et al. [62]. Justifying research priority setting by a lack of knowledge of stakeholders’ research priorities has increased in the last ten years. In the last three years, half of all projects named stakeholder involvement as a reason for conducting research priority setting. Actively involving stakeholders in priority setting was basically not existent until 2010. After that, projects slowly started to establish steering groups and advisory boards in which stakeholders became actively involved.

As to the importance of stakeholder involvement by subject area, the analysis shows that especially those areas that have been trending in the last few years (nursing and care, cancer, mental and behavioral and neurodevelopmental disorders, pregnancy and childbirth, and public health) explicitly named stakeholder involvement as a goal and a reason and actively involved them via their governance structures.

### Methods and approaches for stakeholder involvement

Lastly, this study addresses the question how stakeholders’ research priorities were elicited.

#### Methodological design

Overall, 16 different methods were applied in the 731 identified research priority setting projects. Those were the Delphi technique (27%, n = 258), surveys (21%, n = 206), the JLA methodology (13%, n = 125), workshops (11%, n = 103), focus groups (8%, n = 73), interviews (6%, n = 55), meetings (5%, n = 51), the CHNRI approach (4%, n = 38), group discussions (2%, n = 23), stakeholder consultations (1%, n = 14), webinars (> 1%, n = 6), horizon scans (> 1%, n = 3), the COHRED approach (> 1%, n = 2), the CAM approach (> 1%, n = 1), citizens’ jury (> 1%, n = 1), and the ENHR approach (> 1%, n = 1).

The top ten overall methodological designs were: the Delphi technique (30%, n = 221), the JLA methodology (17%, n = 125), surveys (12%, n = 90), the CHNRI approach (5%, n = 38), workshops (5%, n = 37), surveys and workshops (4%, n = 28), focus groups (2%, n = 16), meetings and surveys (2%, n = 16), focus groups and surveys (2%, n = 13), and focus groups and interviews, group discussions, or meetings (all three rank 10th with 2%, n = 11).

Overall, 42% (n = 331) of the projects used pure quantitative methods and 11% (n = 83) pure qualitative methods to elicit stakeholders’ priorities. Most projects (54%, n = 394) applied a mixed-methods, qualitative-quantitative design.

Around three quarters of all projects (77%, n = 561) applied a single method, followed by 17% (n = 125) two methods, 4% (n = 32) three methods, 2% (n = 12) four methods, and one of the most recent priority setting projects [63] even applied five different methods to elicit stakeholders’ research needs: the Delphi technique, interviews, meetings, surveys, and a workshop. The analysis over time shows that applying a multi-method design to elicit stakeholders’ research priorities has become more and more common in the last ten years. An increasing number of projects has been applying two and in the last five years even three to four different methods.

#### Approaches

When it comes to the approaches to elicit stakeholders’ research priorities, three different approaches are distinguished: the identification approach (how possible research priorities are identified in the first place), the prioritization approach (how all possible research priorities are prioritized), and the consensus finding approach (how the priorities are agreed on).

In 86% (n = 627) of the identified projects, stakeholders were able to nominate their research needs either as a stand-alone approach (70%, n = 511) or combined with a literature review (15%, n = 111), researchers’ nominating priorities (1%, n = 4) or all three approaches combined (n = 1). In the remaining projects, the identification of research priorities was either exclusively based on researcher nomination (6%, n = 47), a literature review (6%, n = 46), or both combined (2%, n = 11). In all these instances, stakeholders did not have a say in identifying possible research priorities.

As to prioritization approaches, stakeholders were most frequently asked to rate priorities on Likert scales (43%, n = 312) or to rank them according to their importance (38%, n = 279). A more deliberative approach was to ask stakeholders to discuss the prioritization of their research needs (20%, n = 147). Less frequently, voting (12%, n = 89) or scoring (5%, n = 40) were chosen as prioritization approaches.

To find consensus on the priorities that stakeholders can agree on, half of all projects opted for a deliberative approach and the other half for a mathematical approach. In 49% (n = 357) of the projects, agreement on the final set of research priorities was directly established via stakeholder deliberation. In 51% (n = 374), consensus on research needs was indirectly found via calculating mean/median/mode ratings, mean/median/mode rankings, standard deviations, percentages, summing up scores, or using specific mathematical formulas.

#### Overall deliberative quality

I also assessed how much stakeholders were given an opportunity to comment on, or deliberate over, the research areas, topics or questions offered to them for prioritization. 11% (n = 83) of the projects did not include any deliberative component in their approaches. In other words, in these projects stakeholders were not able to nominate their priorities during the identification phase and were not given the opportunity to discuss their priorities during the prioritization phase or consensus finding phase. Only in 16% (n = 115) of the projects, stakeholders were given opportunities to deliberate over the priorities throughout the entire process. Most commonly, the deliberative element of a research priority setting project was to ask stakeholders to nominate their priorities in the identification phase (44%, n = 318). Less frequently stakeholders could directly raise their voice and deliberate during the identification and consensus finding phase (23%, n = 171). In the remaining projects, stakeholders were given the chance to deliberate during individual stages of the process (prioritization: n = 4, consensus finding: n = 12, identification and prioritization: n = 22, prioritization and consensus: n = 6).

When looking at the deliberative quality of research priority setting over time, the analysis shows that letting stakeholders nominate their priorities has always been a crucial part of most projects. Since 2006, stakeholders were more and more frequently able to deliberate over their priorities throughout the entire process of priority setting.

Regarding the deliberative quality of research priority setting by subject area, no differences were found. Regardless of the subject area, stakeholders were always able to nominate their priorities in the first stage. As to the prioritization and consensus finding phase, the analysis shows that especially those subject areas that have been trending in the last few years (nursing and care, cancer, mental and behavioral and neurodevelopmental disorders, pregnancy and childbirth, and public health) applied deliberative approaches.

## Discussion

This scoping review aimed to provide a comprehensive overview of stakeholder involvement in research priority setting. It is the first that systematically describes, synthesizes, and evaluates stakeholder involvement in research priority setting. In doing so, it complements existing reviews that have so far have only been conducted for the field of health [12, 15, 16, 20] by including any research priority setting projects on any research area worldwide. From a methodological point of view, this review also displays how a computational approach can fruitfully be utilized for literature reviews.

### Main findings

A computational approach combined with a final manual screening for inclusion identified 731 research priority setting projects published until the end of 2020 that involved stakeholders to set the research agenda.

Until the mid 90’s, research priority setting projects with stakeholder involvement were isolated occurrences. Since the beginning of the 2000s, the number of projects continuously increased. This increase might most probably be a result of an underlying change in the research culture: Awareness of the potential value of research priority setting has risen, explicit values regarding stakeholder involvement have been developed, and more and more voices have been calling to actively involve the public in research. For instance, funders like the British National Institute of Health Research (NIHR) made stakeholder involvement an indispensable condition for funding research projects since 1996. Researchers applying for funding were specifically asked to include plans for involvement within their funding applications [64], and the INVOLVE Foundation was established in the UK to help achieve stakeholder engagement in health research [65]. Furthermore, the foundation of the James Lind Alliance (JLA) in 2003 in the UK as well as the foundation of the Patient Centered Outcome Research Institute (PCORI) in 2010 in the USA boosted collaborations between patients, carers, and health professionals to jointly identify priorities for research. Apparently, involving stakeholders in research priority setting can only be ensured if the corresponding funding and support organizations and structures are present.

As the analysis has shown, priority setting has been nearly exclusively conducted for health research. In doing so, priority setting has been assisting researchers and policymakers in effectively targeting research with the greatest potential public health benefit. Health research prioritization is therefore considered key to strengthen national health research systems and has become essential to maximize the impact of investments especially in resource-poor environments [11]. Other scientific disciplines have barely—if at all—used this approach to identify their major research needs. This is a rather surprising finding as in recent years influential bodies like the EC or the International Science Council (ISC) have advocated mission-oriented research that responds to the grand social, environmental, and economic challenges of our time [6] and one step forward in identifying these grand challenges is effective research priority setting [66].

More than one third of all research priority setting projects worldwide have either been conducted in the UK or the USA. This strong imbalance is also evident at the continental level. Nearly two thirds of all research priority setting projects have been conducted in Europe and North America while in Africa and Asia such projects have hardly ever been realized. It seems that research priority setting can easier be facilitated in high-income countries that have a long tradition in healthcare research and have the academic and structural resources to support healthcare research. In turn, setting research priorities for health issues that are particularly prevalent in low- and middle-income countries could become neglected. And indeed, when looking at the burden of disease [66] and the findings in this study, it seems that those diseases that cause great burden in Africa and Asia—like Malaria, HIV, nutritional deficiencies, diarrhea and common infectious diseases—are rarely chosen as topics for research priority setting.

As to the stakeholder groups that have so far been involved in research priority setting, the findings reveal that experts by profession (i.e., individuals who have expertise due to their formally learned knowledge in higher education or professional experience) have always been involved in research priority setting. Over time, experts by experience (i.e., individuals with direct lived experience) brought their knowledge and perspectives also into priority setting and in the last years, their involvement—especially those of patients and family members/friends/carers—has particularly increased. Two stakeholder groups that have so far been rarely involved in priority setting are funders and policymakers. Funders and policymakers are, however, pivotal in vouching for the credibility and legitimacy of whole priority setting process, for disseminating the priorities to the public, and for calling on researchers to respond to these priorities in the post-priority setting phase [1]. Therefore, any priority setting project would be well advised to involve these two groups as early as possible. Also, ordinary citizens (i.e., ordinary people in general without any particular interest or concern) have so far hardly been involved in research priority setting. Particularly for priority setting projects addressing general topics that affect the public (e.g., the health system, research ethics, data privacy) bringing people, who have no direct interests in the outcomes, to the table might be a worthwhile endeavor.

Regarding the importance of stakeholder involvement in research priority setting, the findings of this review are mixed. Only half of all projects explicitly mentioned to aim to involve stakeholders and justified the research study with a lack of knowledge about stakeholders’ research priorities. But if involving stakeholders is not explicitly highlighted within the objectives and rationales for research priority setting, stakeholder involvement can quickly become mere lip service. Furthermore, in half of all projects the public neither participated nor was actively involved in research priority setting. In only 17% of the identified projects, the public were indeed actively involved by being members of advisory boards or steering groups, co-developing materials and so forth. All in all, stakeholder involvement can then quickly become tokenistic (i.e., a false appearance of inclusiveness), which may result in devaluated stakeholder input [65] and disinterest on the part of stakeholders to become involved in research again. This in turn diminishes the chances of effective uptake and implementation of research evidence and, thus, the overall relevance and value of research.

Furthermore, the way that stakeholders’ research priorities were elicited can be seen critical considering the findings. From a deliberative democracy viewpoint, it is certainly beneficial to use those methods and approaches that have the greatest deliberative (i.e., discursive) potential due to the very positive and diverse effects that deliberation has. Deliberating research priorities helps to elicit more considered opinions on priorities [67], to refine priorities [68], and to ensure that all perspectives are considered [68, 69]. Deliberative approaches in priority setting also foster the understanding for each other’s views [68], and ultimately facilitate broad acceptance of the consensual process and its outcomes [70]. However, the results of this review show that less active, uninformed, and undeliberated methods (like surveys) were frequently chosen to elicit stakeholders’ priorities. Furthermore, the approaches to identify, prioritize, and reach consensus left little room for deliberation. To increase the legitimacy of research priority setting, future projects would, thus, be well advised to take full advantage of the power of deliberation when choosing their methodological design.

### Strengths and limitations of this study

This is the first scoping review that described, synthesized, and evaluated research priority setting with a particular focus on stakeholder involvement. By analyzing any research priority setting projects on any subject area worldwide, this review explores patterns and relations across a wide range of studies thereby creating a comprehensive and well-rounded overview of research priority setting. However, there are some noteworthy limitations.

The literature search was limited to include projects published in English. This naturally excludes any research priority setting projects published in other languages. This is important to consider especially because the number of studies from each country was counted and compared.

While a comprehensive electronic literature search was conducted, it still cannot be ruled out that some research priority setting projects that do not have some sort of published project documentation, may have been overlooked. To counteract this possible limitation, all relevant websites of organizations that themselves conduct research priority setting were screened.

Even though a broad search approach was intentionally chosen to cover the synonyms for priority setting, this procedure might have had some drawbacks. It increased the “noise” in the data (i.e., it increased the number of publications not relevant for this scoping review) which made it more difficult and time consuming to validate the computational approach. It also made the calculations of the STM computationally more demanding. Despite thorough reliability and validity check of the computational approach throughout the entire process, it cannot be ruled out that a project might have mistakenly been excluded from the analysis.

Additionally, this work very well showcases how a computational approach can be fruitfully utilized for literature reviews and thus nicely joins a few other recent applications of this approach [71–73].

### Practical implications

As a practical addition to this review, the Open Innovation in Science Center at the Ludwig Boltzmann Gesellschaft created the first worldwide research priority setting database [2]: https://ois.lbg.ac.at/en/project-database. In doing so, we have fulfilled a frequently expressed wish for an infrastructure to look up and disseminate research priority setting projects [16, 74]. The database contains all the projects analyzed for this scoping review and is also constantly updated with the latest published research priority setting projects. The database provides insights into the general characteristics, stakeholder involvement and methodological designs. The database serves as a reference guide for researchers and any interested persons to look up what research priority setting projects already exist to prevent “research waste” by unnecessarily duplicating prioritization efforts. Moreover, the database is also a source of inspiration for future priority setting projects. The information provided by the database may help researchers to design future research priority setting projects. Additionally, the listed projects may motivate researchers to conduct research on the identified priorities themselves.

## Conclusion

Involving stakeholders at the beginning of the research process, when deciding what to research, can undoubtedly be a very beneficial endeavor. Such involvement not only leads to more direct applicability of research results to stakeholders and better practical uptake, but it also fosters the democratization of research and improves the relevance and legitimacy of research overall.

By mapping out the complex landscape of stakeholder involvement in research priority setting projects, this review guides future efforts to involve stakeholders effectively, inclusively, and transparently, which in turn may increase the overall value of research for society.

However, considering researchers’ still existent skepticism towards the benefits of involving stakeholders in research priority setting [1], future research on this matter is greatly needed. Thus far, there exists anecdotal evidence. Isolated projects have proven that researchers may indeed overlook questions of relevance to stakeholders, and that answering these questions not only satisfies stakeholders’ needs, but also results in more effective research translation [75–78]. A systematic analysis of the extent to which research priority setting generates scientific but most importantly societal impact is yet missing.

## Supplementary Information

**Additional file 1**. Search Strings.

**Additional file 2**. Top Words per Topic.

**Additional file 3**. Coding Scheme.

**Additional file 4**. List of Included Studies.

## Data Availability

The dataset analyzed is available from the corresponding author on reasonable request. The analyzed studies are listed in Additional file 4 and are available online: https://ois.lbg.ac.at/en/project-database.

## Abbreviations

EC: European Commission
ISC: International Science Council
JLA: James Lind Alliance
MRC: Medical Research Council
NHMRC: National Health and Medical Research Council
NIHR: National Institute of Health Research
OECD: Organisation for Economic Co-Operation and Development
PCORI: Patient Centered Outcome Research Institute
R&D: Research and development
WHO: World Health Organization

## Footnotes

1 The terms “agenda setting”, “resource allocation” and “rationing” are sometimes synonymously used with priority setting.

2 In the case of this scoping review, the merged title and abstract of each hit represents one document.

3 The 15 top words for topic number 1 were research, nurse, priority, survey, identify, topic, study, delphi, round, area, rank, practice, result, high, respondent. The 15 top words for topic number 5 were priority, question, group, research, stakeholder, identify, participant, set, method, process, result, prioritize, people, top, engagement.
