# Supplementary material for: Involving stakeholders in research priority setting: a scoping review
Source: Res Involv Engagem. 2021 Oct 29;7:75. doi: 10.1186/s40900-021-00318-6 (PMC8555197; doi:10.1186/s40900-021-00318-6)
Supplement: Supplementary file 1 — Additional file 1. Search Strings. [file 40900_2021_318_MOESM1_ESM.pdf]

Additional File 1: Search Strings

Pubmed:

("priority setting" [Title/Abstract]) OR ("research priorit*" [Title/Abstract]) OR ("priority research" [Title/Abstract]) OR ("research agenda setting" [Title/Abstract]) OR ("agenda setting" [Title/Abstract] + research [Title/Abstract]) OR ("agenda setting" [Title/Abstract] + "priorit*" [Title/Abstract]) OR ("research agenda" [Title/Abstract] + "priorit*" [Title/Abstract]) OR ("resource allocation" [Title/Abstract] + "priorit*" [Title/Abstract]) OR ("allocation of resources" [Title/Abstract] + "priorit*" [Title/Abstract]) OR ("rationing" [Title/Abstract] + "priorit*" [Title/Abstract])

Filter: English

Web of Science:

(TI=("priority setting”) OR AB=("priority setting”)) OR (TI=("priority research”) OR AB=("priority research”)) OR (TI=("research priorit*") OR AB=("research priorit*")) OR (TI=("research agenda setting") OR AB=("research agenda setting")) OR (TI=("agenda setting" AND research) OR AB=("agenda setting" AND research)) OR (TI=("agenda setting" AND priorit*) OR AB=("agenda setting" AND priorit*)) OR (TI=("research agenda" AND priorit*) OR AB=("research agenda" AND priorit*)) OR (TI=("resource allocation" AND priorit*) OR AB=("resource allocation" AND priorit*)) OR (TI=("allocation of resources" AND priorit*) OR AB=("allocation of resources" AND priorit*)) OR (TI=("rationing” AND priorit*) OR AB=("rationing” AND priorit*)) AND LANGUAGE: (English)

Scopus:

TITLE-ABS("priority setting") OR TITLE-ABS("priority research") OR TITLE-ABS("research priorit*") OR TITLE-ABS("research agenda setting") OR TITLE-ABS("agenda setting" AND research) OR TITLE-ABS("agenda setting" AND priorit*) OR TITLE-ABS("research agenda" AND priorit*) OR TITLE-ABS("resource allocation" AND priorit*) OR TITLE-ABS("rationing” AND priorit*) AND (LIMIT-TO (LANGUAGE , "English")

Google Scholar:

allintitle: priority + setting excluding patents, excluding citations

allintitle: priority + research excluding patents, excluding citations

allintitle: priorities + research excluding patents, excluding citations

allintitle: research + agenda + setting excluding patents, excluding citations

allintitle: agenda + setting + (priority | priorities) excluding patents, excluding citations

allintitle: research + agenda + (priority | priorities) excluding patents, excluding citations

allintitle: resource + allocation + (priority | priorities) excluding patents, excluding citations

allintitle: ("rationing” + priority) | ("rationing” + priority) excluding patents, excluding citations
