# Supplementary figures and images for: Involving stakeholders in research priority setting: a scoping review
Source: Res Involv Engagem. 2021 Oct 29;7:75. doi: 10.1186/s40900-021-00318-6 (PMC8555197; doi:10.1186/s40900-021-00318-6)

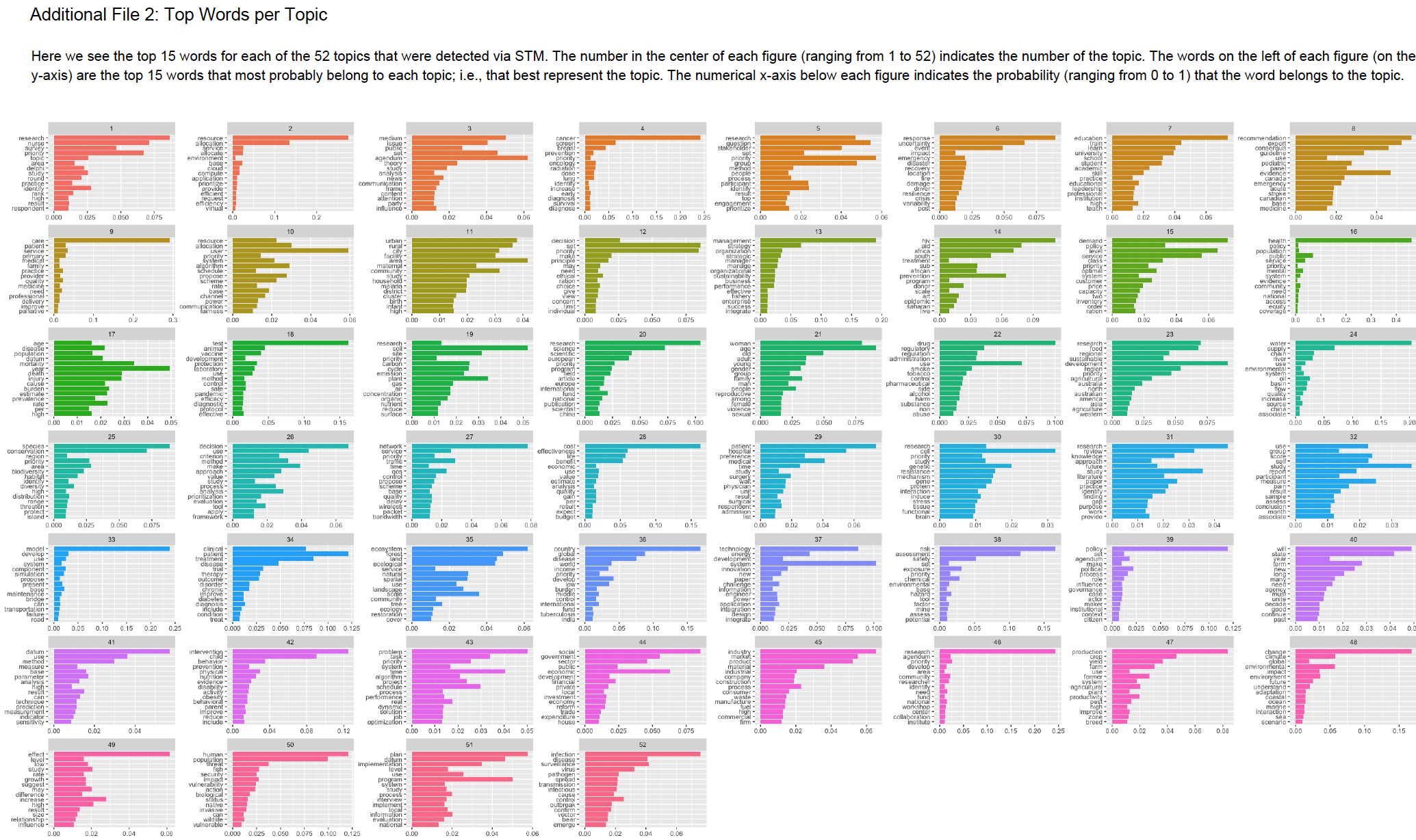

Supplement: Supplementary file 2 — Additional file 2. Top Words per Topic. [file 40900_2021_318_MOESM2_ESM.png]
