# Supplementary material for: Involving stakeholders in research priority setting: a scoping review
Source: Res Involv Engagem. 2021 Oct 29;7:75. doi: 10.1186/s40900-021-00318-6 (PMC8555197; doi:10.1186/s40900-021-00318-6)
Supplement: Supplementary file 3 — Additional file 3. Coding Scheme. [file 40900_2021_318_MOESM3_ESM.pdf]

Additional File 3: Coding Scheme

Subject area: In a first step the specific topic of each priority setting project was extracted and in a subsequent step these topics were inductively classified along subject areas. The subject area health was further classified along the international classification of diseases “ICD-11 for Mortality and Morbidity Statistics” provided by WHO. The subject areas are: agriculture, citizen science, communication, construction, development, education, environment, health - aging, health - animal health, health - blood, health - cancer, health - circulatory system, health - complementary medicine, health - dentistry, health - developmental anomalies, health - digestive system, health - digital health, health - ear, health - emergency medicine, health - endocrine, nutritional and metabolic diseases, health - general symptoms, signs and clinical findings, health - genitourinary system, health - health and patient safety, health - health communication, health - health education, health - health research, health - health system, health - infectious and parasitic diseases, health - injuries, health - mental, behavioral and neurodevelopmental disorders, health - musculoskeletal system and connective tissue, health - nervous system, health - nursing and care, health - nutrition, health - organs and tissues, health - pediatrics, health - physical mobility and fitness, health - pregnancy and childbirth, health - public health, health - respiratory system, health - sexual health, health - skin, health - substance use and addictive behaviors, health - surgery, health - visual system, human resources, law, logistics, social work, technology.

Countries: The specific countries of the research priority setting were extracted.

Stakeholder groups: In a first step the specific stakeholders were extracted and in a subsequent step these stakeholders were inductively classified along broader groups. The stakeholder groups are: academics/researchers, agencies, agriculturists, business professionals, citizens, communication practitioners, construction sector, defense service providers, development practitioners, educators, environmental practitioners, family/friends/carers, financial service providers, funders, healthcare providers, healthcare providers - allied healthcare professionals, healthcare providers - doctors, healthcare providers - nurses, healthcare providers - veterinarian healthcare providers, industry, labor union, learners, legal service providers, NGOs, patients, policymakers, social workers, spiritual service providers, technology practitioners, users/consumers.

Publication year: year of publication

Objective: coding categories: “yes, stakeholder reference”, “no, no stakeholder reference”

Rationale: coding categories: “disease burden”, “general need for research”, “impact”, “more effective use of scarce resources”, “no knowledge of stakeholders’ research priorities”

Governance structure: coding categories: “yes, advisory board”, “yes, steering group”, “no, not reported”

Level of public involvement: coding categories “no, patients/public not involved”, “yes, participation level”, “yes, participation and involvement level”, and “yes, participation level”

Method: coding categories: CAM approach, CHNRI approach, citizens' jury, COHRED approach, consultation, Delphi technique, ENHR approach, focus group, group discussion, horizon scan, interview, JLA method, meeting, survey, webinar, workshop

Identification approach: coding categories: “literature review”, “participant nomination”, “researcher nomination”

Prioritization approach: coding categories: “discussing”, “rating”, “ranking”, “scoring”, “voting”

Consensus finding approach: coding categories: “deliberation”, “mean/median/mode rating”, “mean/median/mode ranking”, “standard deviation”, “percentage”, “score sum”, “other mathematical formula”
