# Supplementary material for: Involving stakeholders in research priority setting: a scoping review
Source: Res Involv Engagem. 2021 Oct 29;7:75. doi: 10.1186/s40900-021-00318-6 (PMC8555197; doi:10.1186/s40900-021-00318-6)
Supplement: Supplementary file 4 — Additional file 4. List of Included Studies. [file 40900_2021_318_MOESM4_ESM.pdf]

Additional File 4: List of Included Studies

1. Abbott C, Diomed B, Johnson J, MacIlraith N. AORN priorities for perioperative nursing research. AORN. 1994;60:914-24.
2. Abu-Rmeileh NME, Ghandour R, Tucktuck M, Obiedallah M. Research priority-setting: reproductive health in the occupied Palestinian territory. Reprod Health. 2018;15:27.
3. Acosta S, Kumlien C, Forsberg A, Nilsson J, Ingemansson R, Gottsäter A. Engaging patients and caregivers in establishing research priorities for aortic dissection. SAGE Open Medicine. 2019;7:205031211882263.
4. Ahmad OF, Mori Y, Misawa M, Kudo S, Anderson JT, Bernal J, et al. Establishing key research questions for the implementation of artificial intelligence in colonoscopy: a modified Delphi method. Endoscopy. 2020; doi: 10.1055/a-1306-7590.
5. Ajjawi R, Barton KL, Dennis AA, Rees CE. Developing a national dental education research strategy: priorities, barriers and enablers. BMJ Open. 2017;7:e013129.
6. Ajumobi O, Uhomoibhi P, Onyiah P, Babalola O, Sharafadeen S, Ughasoro MD, et al. Setting a Nigeria national malaria operational research agenda: the process. BMC Health Serv Res. 2018;18:459.
7. Albrecht MN, Perry KM. Home health care: delineation of research priorities and formation of a national network group. Clin Nurs Res. 1992;1:305-11.
8. Alderson C, Gallimore I, Gorman R, Monahan M, Wojtasinski A. Research priorities of VA nurses: a Delphi study. Military Medicine. 1992;157:462-5.
9. Aldiss S, Fern LA, Phillips RS, Callaghan A, Dyker K, Gravestock H, et al. Research priorities for young people with cancer: a UK priority setting partnership with the James Lind Alliance. BMJ Open. 2019;9:e028119.
10. Ali M, Seuc A, Rahimi A, Festin M, Temmerman M. A global research agenda for family planning: results of an exercise for setting research priorities. Bull World Health Organ. 2014;92:93-8.
11. Al-Khatib SM, Gierisch JM, Crowley MJ, Coeytaux RR, Myers ER, Kendrick A, et al. Future research prioritization: implantable cardioverter-defibrillator therapy in older patients. J Gen Intern Med. 2015;30:1812-20.
12. Allard J, Durand C, Anthony SJ, Dumez V, Hartell D, Hébert M-J, et al. Perspectives of patients, caregivers and researchers on research priorities in donation and transplantation in Canada: a pilot workshop. Transplantat Direct. 2017;3:e127.
13. Allison P, Bedos C. What are the research priorities of Canadian dentists? J Can Dent Assoc. 2002;68:7.
14. Allotey J, Matei A, Husain S, Newton S, Dodds J, Armson AB, et al. Research prioritization of interventions for the primary prevention of preterm birth: an international survey. Eur J Obstet Gynecol Reprod Biol. 2019;236:240-8.
15. Allsopp N, Slingsby JA, Esler KJ. Identifying research questions for the conservation of the Cape Floristic Region. S Afr J Sci. 2019;115. doi:10.17159/sajs.2019/5889.
16. Al-Mawali A, Al-Harrasi A, Jayapal SK, Al-Kharusi H, Al-Rashdi M, Daniel Pinto A. Health research priority setting in Oman: towards better utilization of the available resources. J Contemp Med Sci. 2020;6. doi:10.22317/jcms.v6i3.791.
17. Alsaeed D, Davies N, Gilmartin JF-M, Jamieson E, Kharicha K, Liljas AEM, et al. Older people's priorities in health and social care research and practice: a public engagement workshop. Res Involv Engagem. 2016;2:2.
18. Alshibani A, Banerjee J, Lecky F, Coats TJ, Prest R, Mitchell Á, et al. A consensus building exercise to determine research priorities for silver trauma. BMC Emerg Med. 2020;20:63.
19. Al-Yateem N, Al-Tamimi M, Brenner M, Al Tawil H, Ahmad A, Brownie S, et al. Nurse-identified patient care and health services research priorities in the United Arab Emirates: a Delphi study. BMC Health Serv Res. 2019;19:77.

20. Al-Yateem N, Al-Tamimi M, Brenner M, Altawil H, Ahmad A, Brownie S. Research priorities for specialized nursing practice in the United Arab Emirates. Int Nurs Rev. 2018;65:381-91.

21. Al-Yateem N, Docherty C, Brenner M, Alhosany J, Altawil H, Al-Tamimi M. Research priorities for school nursing in the United Arab Emirates (UAE). J Sch Nurs. 2017;33:393-401.

22. Amato K, Park E, Nigg CR. Prioritizing multiple health behavior change research topics: expert opinions in behavior change science. Behav Med Pract Policy Res. 2016;6:220-7.

23. Angood C, Khara T, Dolan C, Berkley JA, WaSt Technical Interest Group. Research priorities on the relationship between wasting and stunting. PLoS ONE. 2016;11:e0153221.

24. Angood C, McGrath M, Mehta S, Mwangome M, Lung'aho M, Roberfroid D, et al. Research priorities to improve the management of acute malnutrition in infants aged less than six months (MAMI). PLoS Med. 2015;12:e1001812.

25. Annells M, DeRoche M, Koch T, Lewin G, Lucke J. A Delphi study of district nursing research priorities in Australia. Appl Nurs Res. 2005;18:36-43.

26. Araújo V, Teixeira PM, Yaphe J, Correia de Sousa J. The respiratory research agenda in primary care in Portugal: a Delphi study. BMC Fam Pract. 2016;17:124.

27. Ardoin SP, Daly RP, Merzoug L, Tse K, Ardalan K, et al. Research priorities in childhood-onset lupus: results of a multidisciplinary prioritization exercise. Pediatr Rheumatol. 2019;17:32.

28. Armstrong A, Nagata JM, Vicari M, Irvine C, Cluver L, Sohn AH, et al. A global research agenda for adolescents living with HIV. J Acquir Immune Defic Syndr. 2018;78:S16-21.

29. Armstrong MJ, Gamez N, Alliance S, Majid T, Taylor A, Kurasz AM, et al. Research priorities of caregivers and individuals with dementia with Lewy bodies: an interview study. PLoS ONE. 2020;15:e0239279.

30. Arora NK, Mohapatra A, Gopalan HS, Wazny K, Thavaraj V, Rasaily R, et al. Setting research priorities for maternal, newborn, child health and nutrition in India by engaging experts from 256 indigenous institutions contributing over 4000 research ideas: a CHNRI exercise by ICMR and INCLEN. J Glob Health. 2017;7:011002.

31. Ashley EA, McLean A, Chiara F, Feasey N, Jaoko W, Opintan JA, et al. Setting priorities for patient-centered surveillance of drug-resistant infections. Int J Infect Dis. 2020;97:60-5.

32. Asselin J, MacLeod MLP, Dosman JA. National consultation leads to agrivita research to practice plan for Canada. J Agromedicine. 2009;14:179-84.

33. Auais M, French SD, Beaupre L, Giangregorio L, Magaziner J. Identifying research priorities around psycho-cognitive and social factors for recovery from hip fractures: an international decision-making process. Injury. 2018;49:1466-72.

34. Aungsuroch Y, Nhu XHT, Linh TTK, Polsook R, Navicharern R, Gunawan J. Identifying nursing research priorities in Vietnam: a modified Delphi study. Frontiers of Nursing. 2019;6:249-59.

35. Aungsuroch Y, Songnavong C, Tantikosoom P, Phanpaseuth S, Sisoulath A, Gunawan J, et al. Determining nursing research priorities in Lao People's Democratic Republic: a modified Delphi study. Nurs Midwifery Stud. 2020;9:157.

36. Auramo J, Aminoff A, Punakivi M. Research agenda for e-business logistics based on professional opinions. Int Jnl Phys Dist & Log Manage. 2002;32:513-31.

37. Bäck-Pettersson S, Hermansson E, Sernert N, Björkelund C. Research priorities in nursing - a Delphi study among Swedish nurses. J Clin Nurs. 2008;17:2221-31.

38. Badrick E, Cresswell K, Ellis P, Renehan AG, Crosbie EJ, Crosbie P, et al. Top ten research priorities for detecting cancer early. Lancet Public Health. 2019;4:e551.

39. Bahl R, Martines J, Ali N, Bhan MK, Carlo W, Chan KY, et al. Research priorities to reduce global mortality from newborn infections by 2015. Pediatr Infect Dis J. 2009;28:S43-8.

40. Baker JN, Levine DR, Hinds PS, Weaver MS, Cunningham MJ, Johnson L, et al. Research priorities in pediatric palliative care. J Pediatr. 2015;167:467-470.e3.

41. Ball J, Ballinger C, De Iongh A, Dall'Ora C, Crowe S, Griffiths P. Determining priorities for research to

improve fundamental care on hospital wards. Res Involv Engagem. 2016;2:31.

42. Ball L, Barnes K, Laur C, Crowley J, Ray S. Setting priorities for research in medical nutrition education: an international approach. BMJ Open. 2016;6:e013241.

43. Ball L, Barnes K, Leveritt M, Mitchell L, Williams LT, Ball D, et al. Developing research priorities in Australian primary health care: a focus on nutrition and physical activity. Aust J Prim Health. 2017;23:554.

44. Ballesteros Gallego F, Martin C, Allard J, Anthony SJ, Dumez V, Hartell D, et al. Defining future research priorities in donation and organ and stem cell transplantation with patients, families, caregivers, healthcare providers and researchers within the Canadian national transplant research program. Transplant Direct. 2018;4:e360.

45. Banfield MA, Barney LJ, Griffiths KM, Christensen HM. Australian mental health consumers' priorities for research: qualitative findings from the SCOPE for Research project. Health Expect. 2014;17:365-75.

46. Banfield MA, Griffiths KM, Christensen HM, Barney LJ. SCOPE for research: mental health consumers' priorities for research compared with recent research in Australia. Aust N Z J Psychiatry. 2011;45:1078-85.

47. Banfield MA, Morse AR, Gulliver A, Griffiths KM. Mental health research priorities in Australia: a consumer and carer agenda. Health Res Policy Sys. 2018;16:119.

48. Bannigan K, Boniface G, Doherty P, Nicol M, Porter-Armstrong A, Scudds R. Priorities for occupational therapy research in the United Kingdom: executive summary of the POTTER project. Br J Occup Ther. 2008;71:13-6.

49. Baral S, Scheibe A, Sullivan P, Trapence G, Lambert A, Bekker L-G, et al. Assessing priorities for combination HIV prevention research for men who have sex with men (MSM) in Africa. AIDS Behav. 2013;17:60-9.

50. Barrecheguren M, O'Hara K, Wilkens M, Boyd J, Kolda E, Lara B, et al. Research priorities in α_1-antitrypsin deficiency: results of a patients' and healthcare providers' international survey from the EARCO clinical research collaboration. ERJ Open Res. 2020;6:00523-2020.

51. Barrett S, Kristjanson LJ, Sinclair T, Hyde S. Priorities for adult cancer nursing research: a West Australian replication. Cancer Nurs. 2001;24:88-98.

52. Bassett DS, Patton JR, White W, Blalock G, Smith TEC. Research issues in career development and transition: an exploratory survey of professionals in the field. Career Dev Transit Except Individ. 1997;20:81-100.

53. Batchelor JM, Ridd MJ, Clarke T, Ahmed A, Cox M, Crowe S, et al. The Eczema priority setting partnership: a collaboration between patients, carers, clinicians and researchers to identify and prioritize important research questions for the treatment of eczema. Br J Dermatol. 2013;168:577-82.

54. Bayley EW, MacLean SL, Desy P, McMahon M. ENA's Delphi study on national research priorities for emergency nurses in the United States. J Emerg Nurs. 2004;30:12-21.

55. Bell PF, Daly J, Chang EML. A study of the educational and research priorities of registered nurses in rural Australia. Journal of Adv Nurs. 1997;25:794-800.

56. Beneciuk JM, Verstandig D, Taylor C, Scott D, Levin J, Osborne R, et al. Musculoskeletal pain stakeholder engagement and partnership development: determining patient-centered research priorities. Res Involv Engagem. 2020;6:28.

57. Benevides TW, Shore SM, Palmer K, Duncan P, Plank A, Andresen M-L, et al. Listening to the autistic voice: mental health priorities to guide research and practice in autism from a stakeholder-driven project. Autism. 2020;24:822-33.

58. Bennett WL, Robinson KA, Saldanha IJ, Wilson LM, Nicholson WK. High priority research needs for gestational diabetes mellitus. J Women Health. 2012;21:925-32.

59. Bermudez LG, Williamson K, Stark L. Setting global research priorities for child protection in humanitarian action: results from an adapted CHNRI exercise. PLoS ONE. 2018;13:e0202570.

60. Bessa A, Maclennan S, Enting D, Bryan R, Josephs D, Hughes S, et al. Consensus in bladder cancer research priorities between patients and healthcare professionals using a four-stage modified Delphi method. Eur Urol. 2019;76:258-9.

61. Bethell J, Pringle D, Chambers LW, Cohen C, Commisso E, Cowan K, et al. Patient and public involvement in identifying dementia research priorities: priorities for research on dementia. J Am Geriatr Soc. 2018;66:1608-12.

62. Bethell J, Puts MTE, Sattar S, Andrew MK, Choate AS, Clarke B, et al. The Canadian frailty priority setting partnership: research priorities for older adults living with frailty. Can Geri J. 2019;22:23-33.

63. Bialy L, Plint AC, Freedman SB, Johnson DW, Curran JA, Stang AS, et al. Pediatric emergency research Canada (PERC): patient/family-informed research priorities for pediatric emergency medicine. Acad Emerg Med. 2018;25:1365-74.

64. Birnie KA, Dib K, Ouellette C, Dib MA, Nelson K, Pahtayken D, et al. Partnering for pain: a priority setting partnership to identify patient-oriented research priorities for pediatric chronic pain in Canada. CMAJ Open. 2019;7:E654-64.

65. Bissett M, Cusick DA, Adamson L. Occupational therapy research priorities in mental health. Occup Ther Health Care. 2002; 14:1-19.

66. Blackwood B, Albarran JW, Latour JM. Research priorities of adult intensive care nurses in 20 European countries: a Delphi study. J Adv Nurs. 2011;67:550-62.

67. Blum NJ, Feldman HM, Barbaresi WJ, Schonfeld DJ, Hansen RL. Research priorities for developmental-behavioral pediatrics: a DBPNet consensus study. Behav Pediatr. 2012;33:8.

68. Bogosian A, Rixon L, Hurt CS. Prioritising target non-pharmacological interventions for research in Parkinson's disease: achieving consensus from key stakeholders. Res Involv Engagem. 2020;6:35.

69. Bond S, Bond J. A Delphi survey of clinical nursing research priorities. J Adv Nurs. 1982;7:565-75.

70. Boney O, Bell M, Bell N, Conquest A, Cumbers M, Drake S, et al. Identifying research priorities in anaesthesia and perioperative care: final report of the joint National Institute of Academic Anaesthesia/James Lind Alliance research priority setting partnership. BMJ Open. 2015;5:e010006.

71. Bosco AM, Williams N, Graham JM, Malagas DL, Hauck Y. Developing research priorities for nurses working in the gynaecology setting in Western Australia. Collegian. 2018;25:73-80.

72. Bowling A, Jacobson B, Southgate L. Explorations in consultation of the public and health professionals on priority setting in an inner London health district. Soc Sci Med. 1993;37:851-7.

73. Bradley JM, Madge S, Morton AM, Quittner AL, Elborn JS. Cystic fibrosis research in allied health and nursing professions. J Cyst Fibros. 2012;11:387-92.

74. Brazil K, Maitland J, Ploeg J, Denton M. Identifying research priorities in long term care homes. J Am Med Dir Assoc. 2012;13:84.e1-84.e4.

75. Breault LJ, Rittenbach K, Hartle K, Babins-Wagner R, de Beaudrap C, Jasaui Y, et al. The top research questions asked by people with lived depression experience in Alberta: a survey. CMAJ Open. 2018;6:E398-405.

76. Brender J, Nøhr C, McNair P. Research needs and priorities in health informatics. Int J Med Inform. 2000;58-59:257-89.

77. Brenner M, Hilliard C, Regan G, Coughlan B, Hayden S, Drennan J, et al. Research priorities for children's nursing in Ireland: a Delphi Study. J Pediatr Nurs. 2014;29:301-8.

78. Bressan S, Titomanlio L, Gomez B, Mintegi S, Gervaix A, Parri N, et al. Research priorities for European paediatric emergency medicine. Arch Dis Child. 2019;104:869-73.

79. Brewer SE, Crump NM, O'Leary ST. Patient-centered research priorities: a mixed-methods approach from the Colorado children's outcomes network (COCONet). J Am Board Fam Med. 2019;32:674-84.

80. Bridges JFP, Janssen EM, Ferris A, Dy SM. Project Transform: engaging patient advocates to share their perspectives on improving research, treatment and policy. Curr Med Res Opin. 2018;34:1755-62.

81. Bright O-JM, Wang DD, Shams-White M, Bleich SN, Foreyt J, Franz M, et al. Research priorities for studies linking intake of low-calorie sweeteners and potentially related health outcomes: research methodology and study design. Curr Dev Nutr. 2017;1:e000547.

82. Britton J, Gadeke L, Lovat L, Hamdy S, Hawkey C, McLaughlin J, et al. Research priority setting in Barrett's

oesophagus and gastro-oesophageal reflux disease. Lancet Gastroenterol Hepatol. 2017;2:824-31.

83. Bröchner J, Josephson P, Alte J. Identifying management research priorities. Constr Manag Econ. 2005;23:793-6.

84. Brocklehurst PR, Mackay L, Goldthorpe J, Pretty IA. Older people and oral health: setting a patient-centred research agenda. Gerodontology. 2015;32:222-8.

85. Broerse JEW, Zweekhorst MBM, van Rensen AJML, de Haan MJM. Involving burn survivors in agenda setting on burn research: an added value? Burns. 2010;36:217-31.

86. Broome ME, Woodring B, O'Connor-Von S. Research priorities for the nursing of children and their families: a Delphi study. J Pediatr Nurs. 1996;11:281-7.

87. Brower HT, Crist MA. Research priorities in gerontologic nursing for long-term care. Image J Nurs Sch. 1985;17:22-7.

88. Brown K, Dyas J, Chahal P, Khalil Y, Riaz P, Cummings-Jones J. Discovering the research priorities of people with diabetes in a multicultural community. Br J Gen Pract. 2006;56:206-13.

89. Brown KH, Hess SY, Boy E, Gibson RS, Horton S, Osendarp SJ, et al. Setting priorities for zinc-related health research to reduce children's disease burden worldwide: an application of the Child Health and Nutrition Research Initiative's research priority-setting method. Public Health Nutr. 2009;12:389-396.

90. Browne LR, Shah MI, Studnek JR, Farrell BM, Mattrisch LM, Reynolds S, et al. 2015 Pediatric research priorities in prehospital care. Prehosp Emerg Care. 2016;20:311-6.

91. Browne N, Robinson L, Richardson A. A Delphi study on the research priorities of European oncology nurses. Eur J Oncol Nurs. 2002;6:133-44.

92. Brunsdon D, Biesty L, Brocklehurst P, Brueton V, Devane D, Elliott J, et al. What are the most important unanswered research questions in trial retention? A James Lind Alliance priority setting partnership: the PRioRiTy II (prioritising retention in randomised trials) study. Trials. 2019;20:593.

93. Bryan S, Goldsmith LJ, Suryaprakash N, Sawatzky R, Mulldoon M, Le Mercier M, et al. A research agenda to improve patients' experience of knee replacement surgery: a patient-oriented modified Delphi study of patients of South Asian origin in British Columbia. CMAJ Open. 2020;8:E226-33.

94. Brys N, Keating JA, Knobloch MJ, Safdar N. Engaging patients in health care epidemiology research: a case example. Am J Infect Control. 2019;47:139-43.

95. Buckley BS, Grant AM, Tincello DG, Wagg AS, Firkins L. Prioritizing research: patients, carers, and clinicians working together to identify and prioritize important clinical uncertainties in urinary incontinence. Neurourol Urodyn. 2009;29:708-14.

96. Burgers JS, Wittenberg J, Keuken DG, Dekker F, Hohmann FP, Leereveld D, et al. Development of a research agenda for general practice based on knowledge gaps identified in Dutch guidelines and input from 48 stakeholders. Eur J Gen Pract. 2019;25:19-24.

97. Burnette D, Morrow-Howell N, Chen L-M. Setting priorities for gerontological social work research: a national Delphi study. Gerontologist. 2003;43:828-38.

98. Burns LJ, Abbetti B, Arnold SD, Bender J, Doughtie S, El-Jawahiri A, et al. Engaging patients in setting a patient-centered outcomes research agenda in hematopoietic cell transplantation. Biol Blood Marrow Transplant. 2018;24:1111-8.

99. Bursuck WD, Epstein MH. Current research topics in learning disabilities. Learn Disabil Q. 1987;10:2-7.

100. Burt CG, Cima RR, Koltun WA, Littlejohn CE, Ricciardi R, Temple LK, et al. Developing a research agenda for the American society of colon and rectal surgeons: results of a Delphi approach. Dis Colon Rectum. 2009;52:898-905.

101. Butler MM, Meehan TC, Kemple M, Drennan J, Treacy M, Johnson M. Identifying research priorities for midwifery in Ireland. Midwifery. 2009;25:576-87.

102. Butow P, Shaw J, Vaccaro L, Sharpe L, Dhillon H, Smith B, et al. A research agenda for fear of cancer recurrence: a Delphi study conducted in Australia. Psycho-Oncology. 2019;28:989-96.

103. Buzzard IM, Sievert YA. Research priorities and recommendations for dietary assessment methodology.

Am J Clin Nutr. 1994;59:275S-280S.

104. Byham-Gray LD, Peters EN, Rothpletz-Puglia P. Patient-centered model for protein-energy wasting: stakeholder deliberative panels. J Ren Nutr. 2020;30:137-44.

105. Byles J, Parkinson L, Nair B (Kichu), Watson J, Valentine M. Determining priorities for research in ageing: a community survey. Australas J Ageing. 2007;26:71-6.

106. Byrne S, Wake M, Blumberg D, Dibley M. Identifying priority areas for longitudinal research in childhood obesity: Delphi technique survey. Int J Pediatr Obes. 2008;3:120-2.

107. Camden C, Meziane S, Maltais D, Cantin N, Brossard-Racine M, Berbari J, et al. Research and knowledge transfer priorities in developmental coordination disorder: results from consultations with multiple stakeholders. Health Expect. 2019;22:1156-64.

108. Campbell RA, Howard CA. Priorities for forestry herbicide application technology research. Can J For Res. 1993;23:2204-12.

109. Canavati SE, Lawford HLS, Fatunmbi BS, Lek D, Top-Samphor N, Leang R, et al. Establishing research priorities for malaria elimination in the context of the emergency response to artemisinin resistance framework-the Cambodian approach. Malar J. 2016;15:120.

110. Caron-Flinterman J, Broerse JEW, Teerling J, Bunders JFG. Patients’ priorities concerning health research: the case of asthma and COPD research in the Netherlands. Health Expect. 2005;8:253-63.

111. Carrougher GJ, Marvin JA, Bayley EW, Knighton CJ, Rutan RL, Weber B. Research priorities for burn nursing report of the wound care and infection control group. J Burn Care Rehabil. 1991;12:272-7.

112. Cassiani SHDB, Bassalobre-Garcia A, Reveiz L. Universal access to health and universal health coverage: identification of nursing research priorities in Latin America. Rev Latino-Am Enfermagem. 2015;23:1195-208.

113. Cavers D, Cunningham-Burley S, Watson E, Banks E, Campbell C. Setting the research agenda for living with and beyond cancer with comorbid illness: reflections on a research prioritisation exercise. Res Involv Engagem. 2020;6:17.

114. Cawley N, Webber J. Research priorities in palliative care. Int J Palliat Nurs. 1995;1:101-13.

115. Chamberlain SA, Estabrooks CA, Keefe JM, Hoben M, Berendonk C, Corbett K, et al. Citizen and stakeholder led priority setting for long-term care research: identifying research priorities within the Translating Research in Elder Care (TREC) Program. Res Involv Engagem. 2020;6:24.

116. Chan G, Storey JD, Das MK, Sacks E, Johri M, Kabakian-Khasholian T, et al. Global research priorities for social, behavioural and community engagement interventions for maternal, newborn and child health. Health Res Policy Sys. 2020;18:97.

117. Chang E, Bing Ho CK, Man Yuen AC, Hatcher D. A study of clinical nursing research priorities in aged care: a Hong Kong perspective. Contemp Nurse. 2003;15:188-98.

118. Chang E, Daly J. Clinical research priorities in oncology nursing: an Australian perspective. Int J Nurs Pract. 1996;2:21-8.

119. Chang E, Daly J. Priority areas for clinical research in palliative care nursing. Int J Nurs Pract. 1998;4:247-53.

120. Chapman E, Reveiz L, Sangalang S, Manu C, Bonfill X, Muñoz S, et al. A survey study identified global research priorities for decreasing maternal mortality. J Clin Epidemiol. 2014;67:314-24.

121. Chess C, Salomone KL, Hance BJ. Improving risk communication in government: research priorities. Risk Analysis. 1995;15:127-35.

122. Cheyne H, McCourt C, Semple K. Mother knows best: developing a consumer led, evidence informed, research agenda for maternity care. Midwifery. 2013;29:705-12.

123. Childs N, Robinson L, Chowdhury S, Ogden C, Newton JL. Consulting patients in setting priorities in Myalgic Encephalomyelitis (M.E.) research: findings from a national on-line survey. Res Involv Engagem. 2015;1:11.

124. Clarence C, Shiras T, Zhu J, Boggs MK, Faltas N, Wadsworth A, et al. Setting global research priorities for

private sector child health service delivery: results from a CHNRI exercise. Journal Glob Health. 2020;10:021201.

125. Clark DC, Babich G, Burpeau MY. Research needs in teacher activities: results of a national survey. J Early Adolesc. 1981;1:391-5.

126. Clark M, Adams D. Listening to parents to understand their priorities for autism research. PLoS ONE. 2020;15:e0237376.

127. Clinton-McHarg T, Paul C, Sanson-Fisher R, D'Este C, Williamson A. Determining research priorities for young people with haematological cancer: a value-weighting approach. Eur J Cancer. 2010;46:3263-70.

128. Cohen MZ, Harle M, Woll AM, Despa S, Munsell MF. Delphi survey of nursing research priorities. Oncol Nurs Forum. 2004;31:1011-8.

129. Colagiuri R, Boylan S, Morrice E. Research priorities for NCD prevention and climate change: an international Delphi survey. Int J Environ Res Public Health. 2015;12:12941-57.

130. Coller RJ, Berry JG, Kuo DZ, Kuhlthau K, Chung PJ, Perrin JM, et al. Health system research priorities for children and youth with special health care needs. Pediatrics. 2020;145:e20190673.

131. Collins PY, Patel V, Joestl SS, March D, Insel TR, Daar AS, Bordin IA, Costello EJ, Durkin M, Fairburn C, Glass RI. Grand challenges in global mental health. Nature. 2011;475:27-30.

132. Colucci E, Too LS, Minas H. A suicide research agenda for people from immigrant and refugee backgrounds. Death Studies. 2017;41:502-11.

133. Considine J, Curtis K, Shaban RZ, Fry M. Consensus-based clinical research priorities for emergency nursing in Australia. Australas Emerg Care. 2018;21:43-50.

134. Coole C, McBean J, Drummond A, Reagon C. The identification of research priorities for UK occupational therapists in work rehabilitation. Int J Ther Rehabil. 2015;22:329-36.

135. Cooper RA, Quatrano LA, Axelson PW. Research on physical activity and health among people with disabilities: a consensus statement. J Rehabil R D. 1999;36:142-153.

136. Coppack RJ, Ladlow P, Bennett AN. Developing UK defence rehabilitation research priorities: a 2020 clinical practitioner engagement exercise. BMJ Mil Health. 2020; doi: 10.1136/bmjmilitary-2020-001676.

137. Corner J, Wright D, Hopkinson J, Gunaratnam Y, McDonald JW, Foster C. The research priorities of patients attending UK cancer treatment centres: findings from a modified nominal group study. Br J Cancer. 2007;96:875-81.

138. Correll CK, Dave M, Paul AF, Gaizo VD, Schrandt S, Partovi RS, et al. Identifying research priorities among patients and families of children with rheumatic diseases living in the United States. J Rheumatol. 2020;47:1800-6.

139. Costa L da CM, Koes BW, Pransky G, Borkan J, Maher CG, Smeets RJEM. Primary care research priorities in low back pain: an update. Spine. 2013;38:148-56.

140. Cotts T, Khairy P, Opotowsky AR, John AS, Valente AM, Zaidi AN, et al. Clinical research priorities in adult congenital heart disease. Int J Cardiol. 2014;171:351-60.

141. Cowman S, Björkdahl A, Clarke E, Gethin G, Maguire J. A descriptive survey study of violence management and priorities among psychiatric staff in mental health services, across seventeen European countries. BMC Health Serv Res. 2017;17:59.

142. Cowman S, Gethin G, Clarke E, Moore Z, Craig G, Jordan-O'Brien J, et al. An international eDelphi study identifying the research and education priorities in wound management and tissue repair: eDelphi study on education and research priorities in wound management. J Clin Nurs. 2012;21:344-53.

143. Cox J, Halkett G, Anderson C, Heard R. A Delphi study on research priorities in radiation therapy: the Australian perspective. Radiography. 2010;16:26-39.

144. Crews DC, Greer RC, Fadrowski JJ, Choi MJ, Doggett D, Segal JB, et al. Setting an agenda for comparative effectiveness systematic reviews in CKD care. BMC Nephrol. 2012;13:74.

145. Crowley MJ, McCrory DC, Chatterjee R, Gierisch JM, Myers ER, Schmit KM, et al. Prioritization of research addressing antipsychotics for adolescents and young adults with bipolar disorder. Ann Intern Med.

2014;160:492.

146. Cullum N, Buckley H, Dumville J, Hall J, Lamb K, Madden M, et al. Wounds research for patient benefit: a 5-year programme of research. Southampton (UK): NIHR Journals Library.

147. Curtis K, Nahidi S, Gabbe B, Vallmuur K, Martin K, Shaban RZ, et al. Identifying the priority challenges in trauma care delivery for Australian and New Zealand trauma clinicians. Injury. 2020;51:2053-8.

148. Dagenais C, Degroote S, Otmani Del Barrio M, Bermudez-Tamayo C, Ridde V. Establishing research priorities in prevention and control of vector-borne diseases in urban areas: a collaborative process. Infect Dis Poverty. 2018;7:85.

149. Daly JP, Chang EM. A study of clinical nursing research priorities of renal specialist nurses caring for critically ill people. Intensive Crit Care Nurs. 1996;12:45-9.

150. Daly J, Chang EML. Clinical nursing research priorities in Australian critical care: a pilot study. J Adv Nurs. 1996;23:145-51.

151. Damen A, Delaney A, Fitchett G. Research priorities for healthcare chaplaincy: views of U.S. chaplains. J Health Care Chaplain. 2018;24:57-66.

152. Damen A, Schuhmann C, Lensvelt-Mulders G, Leget C. Research priorities for health care chaplaincy in the Netherlands: a Delphi study among Dutch chaplains. J Health Care Chaplain. 2020;26:87-102.

153. Danforth K. Comparative effectiveness topics from a large, integrated delivery system. Perm J. 2013;17:4-13.

154. Daniels L, Howlett C. The way forward: identifying palliative nursing research priorities within a hospice. Int J Palliat Nurs. 2001;7:442-8.

155. Davidson P, Merritt-Gray M, Buchanan J, Noel J. Voices from practice: mental health nurses identify research priorities. Arch Psychiatr Nurs. 1997;11:340-5.

156. Davies GF, Greenhough BJ, Hobson-West P, Kirk RGW, Applebee K, Bellingan LC, et al. Developing a collaborative agenda for humanities and social scientific research on laboratory animal science and welfare. PLoS ONE. 2016;11:e0158791.

157. Davila-Seijo P, Hernández-Martín A, Morcillo-Makow E, Lucas R de, Domínguez E, Romero N, et al. Prioritization of therapy uncertainties in Dystrophic Epidermolysis Bullosa: where should research direct to? An example of priority setting partnership in very rare disorders. Orphanet J Rare Dis. 2013;8:61.

158. Davison KM, D'Andreamatteo C, Mitchell S, Vanderkooy P. The development of a national nutrition and mental health research agenda with comparison of priorities among diverse stakeholders. Public Health Nutr. 2017;20:712-25.

159. de Haan S, Kingamkono R, Tindamanyire N, Mshinda H, Makandi H, Tibazarwa F, et al. Setting research priorities across science, technology, and health sectors: the Tanzania experience. Health Res Policy Sys. 2015;13:14.

160. de Vries K, Walton J, Nelson K, Knox R. An examination of the research priorities for a hospice service in New Zealand: a Delphi study. Pall Supp Care. 2016;14:232-40.

161. Dean R. Identification of research priorities of veterinary surgeons and cat owners with regard to the treatment of chronic kidney disease (CKD) in cats. 2014. https://www.nottingham.ac.uk/cevm/practice-based-research/small-animal/chronic-kidney-disease-in-cats.aspx. Accessed 22 July 2021.

162. Deane HC, Wilson CL, Babl FE, Dalziel SR, Cheek JA, Craig SS, et al. PREDICT prioritisation study: establishing the research priorities of paediatric emergency medicine physicians in Australia and New Zealand. Emerg Med J. 2018;35:39-45.

163. Deane KHO, Flaherty H, Daley DJ, Pascoe R, Penhale B, Clarke CE, et al. Priority setting partnership to identify the top 10 research priorities for the management of Parkinson's disease. BMJ Open. 2014;4:e006434.

164. Dennis AA, Cleland JA, Johnston P, Ker JS, Lough M, Rees CE. Exploring stakeholders' views of medical education research priorities: a national survey. Med Educ. 2014;48:1078-91.

165. Dennis KE, Howes DG, Zelauskas B. Identifying nursing research priorities: a first step in program

development. Appl Nurs Res. 1989;2:108-13.

166. Dennis L, Brealey S, Rangan A, Rookmoneea M, Watson J. Managing idiopathic frozen shoulder: a survey of health professionals' current practice and research priorities. Shoulder & Elbow. 2010;2:294-300.

167. Dewa LH, Murray K, Thibaut B, Ramtale SC, Adam S, Darzi A, et al. Identifying research priorities for patient safety in mental health: an international expert Delphi study. BMJ Open. 2018;8:e021361.

168. Dibley L, Bager P, Czuber-Dochan W, Farrell D, Jelsness-Jørgensen L-P, Kemp K, et al. Identification of research priorities for inflammatory bowel disease nursing in Europe: a nurses-European Crohn's and Colitis organisation Delphi survey. J Crohns Colitis. 2016;11:353-359.

169. Diffin J, Spence M, Spencer R, Mellor P, Grande G. Involving healthcare professionals and family carers in setting research priorities for end-of-life care. Int J Palliat Nurs. 2017;23:56-9.

170. DiIorio C, Hinkle JL, Stuifbergen A, Algase D, Amidei CS, Austin J, et al. Updated research priorities for neuroscience nursing. J Neurosci Nurs. 2011;43:149-55.

171. Dimmitt C, Carey JC, McGannon W, Henningson I. Identifying a school counseling research agenda: a Delphi study. Counselor Education and Supervision. 2005;44:214-28.

172. Dixon S, Agha K, Ali F, El-Hindi L, Kelly B, Locock L, et al. Female genital mutilation in the UK- where are we, where do we go next? Involving communities in setting the research agenda. Res Involv Engagem. 2018;4:29.

173. Doolan-Noble F, Mehta P, Waters D, Baxter GD. Supporting ageing well research: findings from a research priority setting exercise. Australas J Ageing. 2019;38:136-43.

174. Downing J, Knapp C, Muckaden MA, Fowler-Kerry S, Marston J. Priorities for global research into children's palliative care: results of an International Delphi Study. BMC Palliat Care. 2015;14:36.

175. Dowsett M, Goldhirsch A, Hayes DF, Senn H-J, Wood W, Viale G. International web-based consultation on priorities for translational breast cancer research. Breast Cancer Res. 2007;9:R81.

176. Drake C, Wald HL, Eber LB, Trojanowski JI, Nearing KA, Boxer RS. Research priorities in post-acute and long-term care: results of a stakeholder needs assessment. J Am Med Dir Assoc. 2019;20:911-5.

177. Drennan J, Meehan T, Kemple M, Johnson M, Treacy M (Pearl), Butler M. Nursing research priorities for Ireland. J Nurs Scholarsh. 2007;39:298-305.

178. Drury NE, Stoll VM, Bond CJ, Patel AJ, Hutchinson S, Clift PF. Research priorities in single-ventricle heart conditions: a United Kingdom national study. Cardiol Young. 2019;29:303-9.

179. Dudley N, Hockings M, Stolton S, Amend T, Badola R, Bianco M, et al. Priorities for protected area research. Parks. 2018;24:35-50.

180. Duffy JMN, Adamson GD, Benson E, Bhattacharya S, Bhattacharya S, Bofill M, et al. Top 10 priorities for future infertility research: an international consensus development study. Hum Reprod. 2020;35:2715-24.

181. Duncan EAS, Munro K, Nicol MM. Research priorities in forensic occupational therapy. Br J Occup Ther. 2003;66:55-64.

182. Dwyer M. A Delphi survey of research priorities and identified areas for collaborative research in health sector library and information services UK. Health Libr Rev. 1999;16:174-91.

183. Dzidowska M, Price M, Butow P. Identifying research priorities and research needs among health and research professionals in psycho-oncology: research priorities in psycho-oncology. Asia Pac J Clin Oncol. 2010;6:165-72.

184. Eberman LE, Walker SE, Floyd RT, Covassin T, Nolton E, Valier ARS, et al. The prioritized research agenda for the athletic training profession: a report from the strategic alliance research agenda task force. J Athl Train. 2019;54:237-44.

185. Edwards L, Monro M, Butterfield Y, Johl R, Loftsgard KC, Pelletier H, et al. What matters most to patients about primary healthcare: mixed-methods patient priority setting exercises within the PREFeR (PRioritiEs For Research) project. BMJ Open. 2019;9:e025954.

186. Egestad H, Halkett GKB. A Delphi study on research priorities in radiation therapy: the Norwegian perspective. Radiography. 2016;22:65-70.

187. Eldredge JD, Harris MR, Ascher MT. Defining the medical library association research agenda: methodology and final results from a consensus process. J Med Libr Assoc. 2009;97:178-85.

188. Eleftheriadou V, Whitton ME, Gawkrodger DJ, Batchelor J, Corne J, Lamb B, et al. Future research into the treatment of vitiligo: where should our priorities lie? Results of the vitiligo priority setting partnership. Br J Dermatol. 2011;164:530.

189. El-Jardali F, Makhoul J, Jamal D, Ranson MK, Kronfol NM, Tchaghchagian V. Eliciting policymakers' and stakeholders' opinions to help shape health system research priorities in the Middle East and North Africa region. Health Policy Plan. 2010;25:15-27.

190. Elwyn G, Crowe S, Fenton M, Firkins L, Versnel J, Walker S, et al. Identifying and prioritizing uncertainties: patient and clinician engagement in the identification of research questions. J Eval Clin Pract. 2010;16:627.

191. Emrich-Mills L, Hammond LL, Rivett E, Rhodes T, Richmond P, West J. Identifying research priorities for older people's mental health services. Mental Health and Social Inclusion. 2019;23:89-100.

192. Erler CJ, Thompson CB. Determining the national flight nurses association's research priorities. Air Med J. 1995;14:16-20.

193. Erves JC, Mayo-Gamble TL, Malin-Fair A, Boyer A, Joosten Y, Vaughn YC, et al. Needs, priorities, and recommendations for engaging underrepresented populations in clinical research: a community perspective. J Community Health. 2017;42:472-80.

194. Essink DR, Ratsavong K, Bally E, Fraser J, Xaypadith S, Vonglokham M, et al. Developing a national health research agenda for Lao PDR: prioritising the research needs of stakeholders. Global Health Action. 2020;13:1777000.

195. Etchegary H, Bishop L, Street C, Aubrey-Bassler K, Humphries D, Vat LE, et al. Engaging patients in health research: identifying research priorities through community town halls. BMC Health Serv Res. 2017;17:192.

196. Evans C, Rogers S, McGraw C, Battle G, Furniss L. Using consensus methods to establish multidisciplinary perspectives on research priorities for primary care. Prim Health Care Res Dev. 2004;5:52-9.

197. Evidence Aid Priority Setting Group EAPSG. Prioritization of themes and research questions for health outcomes in natural disasters, humanitarian crises or other major healthcare emergencies. PLoS Curr. 2013. doi:10.1371/currents.dis.c9c4f4db9887633409182d2864b20c31.

198. Fackrell K, Stratmann L, Kennedy V, MacDonald C, Hodgson H, Wray N, et al. Identifying and prioritising unanswered research questions for people with hyperacusis: James Lind Alliance Hyperacusis priority setting partnership. BMJ Open. 2019;9:e032178.

199. Faeth P, Hanson L. A research agenda for the energy, water, land, and climate nexus. J Environ Stud Sci. 2016;6:123-6.

200. Farmer EI. A Delphi study of research priorities in tech prep. Journal of Career and Technical Education. 1998;15. doi:10.21061/jcte.v15i1.695.

201. Feary DA, Burt JA, Bauman AG, Al Hazeem S, Abdel-Moati MA, Al-Khalifa KA, et al. Critical research needs for identifying future changes in Gulf coral reef ecosystems. Mar Pollut Bull. 2013;72:406-16.

202. Fenwick J, Butt J, Downie J, Monterosso L, Wood J. Priorities for midwifery research in Perth, Western Australia: a Delphi study. Int J Nurs Pract. 2006;12:78-93.

203. Fernandez MA, Arnel L, Gould J, McGibbon A, Grant R, Bell P, et al. Research priorities in fragility fractures of the lower limb and pelvis: a UK priority setting partnership with the James Lind Alliance. BMJ Open. 2018;8:e023301.

204. Finch CF, Talpey S, Bradshaw A, Soligard T, Engebretsen L. Research priorities of international sporting federations and the IOC research centres. BMJ Open Sport Exerc Med. 2016;2:e000168.

205. Finer S, Robb P, Cowan K, Daly A, Shah K, Farmer A. Setting the top 10 research priorities to improve the health of people with Type 2 diabetes: a Diabetes UK-James Lind Alliance priority setting partnership. Diabet Med. 2018;35:862-70.

206. Finlay-Jones A, Elliott EJ, Mayers D, Gailes H, Sargent P, Reynolds N, et al. Community priority setting for fetal alcohol spectrum disorder research in Australia. Int J Popul Data Sci. 2020;5. doi: 10.23889/ijpds.v5i1.1359.

207. Fiorillo A, Luciano M, Del Vecchio V, Sampogna G, Obradors-Tarragó C, Maj M, et al. Priorities for mental health research in Europe: a survey among national stakeholders' associations within the ROAMER project. World Psychiatry. 2013;12:165-70.

208. Fitzcharles M-A, Brachaniec M, Cooper L, Dubin R, Flynn T, Gerhold K, et al. A paradigm change to inform fibromyalgia research priorities by engaging patients and health care professionals. Can J Pain. 2017;1:137-47.

209. Fitzpatrick E, Smith A, Hoffmann E, Trice M. Clinical nursing research priorities: a Delphi study. Clin Nurse Spec. 1991;5:6.

210. Flegg K, Gelkopf MJ, Johnson SA, Dimaras H. The top 10 retinoblastoma research priorities in Canada as determined by patients, clinicians and researchers: a patient-oriented priority-setting partnership. CMAJ Open. 2020;8:E420-8.

211. Fleishman E, Blockstein DE, Hall JA, Mascia MB, Rudd MA, Scott JM, et al. Top 40 priorities for science to inform US conservation and management policy. BioScience. 2011;61:290-300.

212. Fletcher-Johnston M, Marshall SK, Straatman L. Healthcare transitions for adolescents with chronic life-threatening conditions using a Delphi method to identify research priorities for clinicians and academics in Canada: priorities in adolescent transitions. Child Care Health Dev. 2011;37:875-82.

213. Fochtman D, Hinds PS. Identifying nursing research priorities in a pediatric clinical trials cooperative group: the pediatric oncology group experience. J Pediatr Oncol Nurs. 2000;17:83-7.

214. Fontaine O, Kosek M, Bhatnagar S, Boschi-Pinto C, Chan KY, Duggan C, et al. Setting research priorities to reduce global mortality from childhood diarrhoea by 2015. PLoS Med. 2009;6:e1000041.

215. Foster J, Bautista C, Ellstrom K, Kalowes P, Manning J, Pasek TA. Creating a research agenda and setting research priorities for clinical nurse specialists. Clin Nurse Spec. 2018;32:21-8.

216. Fountain L, Tofa M, Haynes K, Taylor MR, Ferguson SJ. Older adults in disaster and emergency management: what are the priority research areas in Australia? Int J Disaster Risk Reduct. 2019;39:101248.

217. Francis N, Kazaryan AM, Pietrabissa A, Goitein D, Yiannakopoulou E, Agresta F, et al. A research agenda for the European Association for Endoscopic Surgeons (EAES). Surg Endosc. 2017;31:2042-9.

218. Franck LS, McLemore MR, Williams S, Millar K, Gordon AY, Williams S, et al. Research priorities of women at risk for preterm birth: findings and a call to action. BMC Pregnancy Childbirth. 2020;20:10.

219. Frankenberger WD, Pasmann A, Noll J, Abbadessa MK, Sandhu R, Brodecki D, et al. Nursing research priorities in the pediatric emergency care applied research network (PECARN): reaching consensus through the Delphi method. J Emerg Nurs. 2019;45:614-21.

220. Franklin S, Harhen D, Hayes M, Demos Mc Manus S, Pollock A. Top 10 research priorities relating to aphasia following stroke. Aphasiology. 2018;32:1388-95.

221. Frazier TW, Dawson G, Murray D, Shih A, Sachs JS, Geiger A. Brief report: a survey of autism research priorities across a diverse community of stakeholders. J Autism Dev Disord. 2018;48:3965-71.

222. French SD, Beliveau PJH, Bruno P, Passmore SR, Hayden JA, Srbely J, et al. Research priorities of the Canadian chiropractic profession: a consensus study using a modified Delphi technique. Chiropr Man Therap. 2017;25:38.

223. Frison S, Angood C, Khara T, Bahwere P, Black RE, Briend A, et al. Prevention of child wasting: results of a Child Health & Nutrition Research Initiative (CHNRI) prioritisation exercise. PLoS ONE. 2020;15:e0228151.

224. Fudim M, Dalgaard F, Al-Khatib SM, J. Friedman D, Lallinger K, Abraham WT, et al. Future research prioritization in cardiac resynchronization therapy. Am Heart J. 2020;223:48-58.

225. Furyk J, Ray R, Watt K, Dalziel SR, Oakely Ed, Mackay M, et al. Consensus research priorities for paediatric status epilepticus: a Delphi study of health consumers, researchers and clinicians. Seizure. 2018;56:104-9.

226. Gadsby R, Snow R, Daly AC, Crowe S, Matyka K, Hall B, et al. Setting research priorities for Type 1 diabetes: setting research priorities for Type 1 diabetes. Diabet Med. 2012;29:1321-6.

227. García D, Brazal S, Lindenberg F, Toft E, La Marca A, Borrás R, et al. Identification of research priorities in infertility and assisted reproduction: an international, multicentre study. Reprod Biomed Online. 2020;40:238-44.

228. Gaw S, Harford A, Pettigrove V, Sevicke-Jones G, Manning T, Ataria J, et al. Towards sustainable environmental quality: priority research questions for the Australasian region of Oceania. Integr Environ Assess Manag. 2019;15:917-35.

229. George M, Hernandez C, Smith S, Narsavage G, Kapella MC, Carno M, et al. Nursing research priorities in critical care, pulmonary, and sleep: international Delphi survey of nurses, patients, and caregivers: an official American thoracic society workshop report. Annals ATS. 2020;17:1-10.

230. Ghisoni M, Wilson CA, Morgan K, Edwards B, Simon N, Langley E, et al. Priority setting in research: user led mental health research. Res Involv Engagem. 2017;3:4.

231. Giangregorio LM, MacIntyre NJ, Heinonen A, Cheung AM, Wark JD, Shipp K, et al. Too fit to fracture: a consensus on future research priorities in osteoporosis and exercise. Osteoporos Int. 2014;25:1465-72.

232. Gierisch JM, Myers ER, Schmit KM, Crowley MJ, McCrory DC, Chatterjee R, et al. Prioritization of research addressing management strategies for ductal carcinoma in situ. Ann Intern Med. 2014;160:484.

233. Gillespie BM, Walker R, Lin F, Roberts S, Nieuwenhoven P, Perry J, et al. Setting the surgical wound care agenda across two healthcare districts: a priority setting approach. Collegian. 2020;27:529-34.

234. Gillis L, Tomkinson G, Olds T, Moreira C, Christie C, Nigg C, et al. Research priorities for child and adolescent physical activity and sedentary behaviours: an international perspective using a twin-panel Delphi procedure. Int J Behav Nutr Phys Act. 2013;10:112.

235. Glandon D, Meghani A, Jessani N, Qiu M, Bennett S. Identifying health policy and systems research priorities on multisectoral collaboration for health in low-income and middle-income countries. BMJ Glob Health. 2018;3 Suppl 4:e000970.

236. Goldfrad C, Vella K, Bion JF, Rowan KM, Black NA. Research priorities in critical care medicine in the UK. Intensive Care Med. 2000;26:1480-8.

237. Gomes F, Bourassa MW, Adu-Afarwuah S, Ajello C, Bhutta ZA, Black R, et al. Setting research priorities on multiple micronutrient supplementation in pregnancy. Ann NY Acad Sci. 2020;1465:76-88.

238. Goodyear-Smith F, Bazemore A, Coffman M, Fortier R, Howe A, Kidd M, et al. Primary care research priorities in low-and middle-income countries. Ann Fam Med. 2019;17:31-5.

239. Goodyear-Smith F, Bazemore A, Coffman M, Fortier R, Howe A, Kidd M, et al. Primary care financing: a systematic assessment of research priorities in low- and middle-income countries. BMJ Glob Health. 2019;4 Suppl 8:e001483.

240. Goold SD, Myers CD, Danis M, Abelson J, Barnett S, Calhoun K, et al. Members of minority and underserved communities set priorities for health research. The Milbank Quarterly. 2018;96:675-705.

241. Goold SD, Myers CD, Szymecko L, Cunningham Collins C, Martinez S, Ledón C, et al. Priorities for patient-centered outcomes research: the views of minority and underserved communities. Health Serv Res. 2017;52:599-615.

242. Goossens E, Fleck D, Canobbio MM, Harrison JL, Moons P. Development of an international research agenda for adult congenital heart disease nursing. Eur J Cardiovasc Nurs. 2013;12:7-16.

243. Goralnick E, Ezeibe C, Chaudhary MA, McCarty J, Herrera-Escobar JP, Andriotti T, et al. Defining a research agenda for layperson prehospital hemorrhage control: a consensus statement. JAMA Netw Open. 2020;3:e209393.

244. Gordon S, Rotheram-Borus MJ, Skeen S, Perry C, Bryant K, Tomlinson M. Research priorities for the intersection of alcohol and HIV/AIDS in low and middle income countries: a priority setting exercise. AIDS Behav. 2017;21:262-73.

245. Gordon SC, Barry CD. Development of a school nursing research agenda in Florida: A Delphi study. J Sch Nurs. 2006;22:114-9.

246. Graham A, Nester C. Striding ahead - setting the foot health research priorities for the future. 2019. https://footpsp.files.wordpress.com/2019/11/foot-health-psp-final-report.pdf. Accessed 22 July 2021.

247. Grant A, Crane M, Laupacis A, Griffiths A, Burnett D, Hood A, et al. Engaging patients and caregivers in research for pediatric inflammatory bowel disease: top 10 research priorities. J Pediatr Gastroenterol Nutr. 2019;69:317-23.

248. Grant GB, Masresha BG, Moss WJ, Mulders MN, Rota PA, Omer SB, et al. Accelerating measles and rubella elimination through research and innovation - findings from the measles & rubella Initiative research prioritization process, 2016. Vaccine. 2019;37:5754-61.

249. Gray TA, Dumville JC, Christie J, Cullum NA. Rapid research and implementation priority setting for wound care uncertainties. PLoS ONE. 2017;12:e0188958.

250. Green A, Gance-Cleveland B, Smith A, Toly VB, Ely E, McDowell BM. Charting the course of pediatric nursing research: the SPN Delphi study. J Pediatr Nurs. 2014;29:401-9.

251. Gregório G, Tomlinson M, Gerolin J, Kieling C, Moreira HC, Razzouk D, et al. Setting priorities for mental health research in Brazil. Braz J Psychiatry. 2012;34:434-9.

252. Griffin JP, Carroll S, Devos J, Kowba M, Moran J, O'Hare PJ, et al. Nursing research priorities for the care of the naval hospital patient: a Delphi survey. Military Medicine. 1992;157:608-10.

253. Griffin-Sobel JP, Suozzo S. Nursing research priorities for the care of the client with a gastrointestinal disorder: a Delphi survey. Gastroenterol Nurs. 2002;25:188-91.

254. Griffiths KM, Jorm AF, Christensen H, Medway J, Dear KBG. Research priorities in mental health, Part 2: an evaluation of the current research effort against stakeholders' priorities. Aust NZ J Psychiatry. 2002;36:327-39.

255. Gross PH, Bailes AF, Horn SD, Hurvitz EA, Kean J, Shusterman M, et al. Setting a patient-centered research agenda for cerebral palsy: a participatory action research initiative. Dev Med Child Neurol. 2018;60:1278-84.

256. Gulick EE. Research priorities for nurses caring for persons with multiple sclerosis. J Neurosci Nurs. 1996;28:314-22.

257. Gurusamy KS, Walmsley M, Davidson BR, Frier C, Fuller B, Madden A, et al. Top research priorities in liver and gallbladder disorders in the UK. BMJ Open. 2019;9:e025045.

258. Haesler E, Carville K, Haesler P. Priority issues for pressure injury research: an Australian consensus study. Res Nurs Health. 2018;41:355-68.

259. Haghdoost A, Sadeghi M, Nasirian M, Mirzazadeh A, Navadeh S. Research priorities in the field of HIV and AIDS in Iran. Journal of Research in Medical Sciences: The Official Journal of Isfahan University of Medical Sciences. 2012;17:481.

260. Haight BK, Bahr SRT. Setting an agenda for clinical nursing research in long-term care. Clin Nurs Res. 1992;1:144-57.

261. Halcomb EJ, Hickman L. Development of a clinician-led research agenda for general practice nurses. Aust J Adv Nurs. 2010;27:4-11.

262. Halkett GKB, Cox J, Anderson C, Heard R. Establishing research priorities for Australian radiation therapists: what patient care priorities need to be addressed? Eur J Cancer Care. 2012;21:31-40.

263. Hall DA, Mohamad N, Firkins L, Fenton M, Stockdale D. Identifying and prioritizing unmet research questions for people with tinnitus: the James Lind Alliance tinnitus priority setting partnership. Clin Investig. 2013;3:21-8.

264. Hamlet C, Rumsey N, Williamson H, Johnson K, Nduka C. Consensus research priorities for facial palsy: a Delphi survey of patients, carers, clinicians and researchers. J Plast Reconstr Aesthet Surg. 2018;71:1777-84.

265. Hand CL, Letts LJ, von Zweck CM. An agenda for occupational therapy's contribution to collaborative chronic disease research. Can J Occup Ther. 2011;78:147-55.

266. Harper MG, Asselin ME, Kurtz AC, MacArthur SK, Perron S. Research priorities for nursing professional development: a modified e-Delphi study. J Nurses Staff Dev. 2012;28:137-42.

267. Harrington JM. Research priorities in occupational medicine: a survey of United Kingdom medical opinion

by the Delphi technique. Occup Environ Med. 1994;51:289-94.

268. Hart AL, Lomer M, Verjee A, Kemp K, Faiz O, Daly A, et al. What are the top 10 research questions in the treatment of inflammatory bowel disease? A priority setting partnership with the James Lind Alliance. J Crohns Colitis. 2017;11:204-11.

269. Hart LM, Wade T. Identifying research priorities in eating disorders: a Delphi study building consensus across clinicians, researchers, consumers, and carers in Australia. Int J Eat Disord. 2020;53:31-40.

270. Hartshorn S, O'Sullivan R, Maconochie IK, Bevan C, Cleugh F, Lyttle MD. Establishing the research priorities of paediatric emergency medicine clinicians in the UK and Ireland. Emerg Med J. 2015;32:864-8.

271. Hatton JM, Nunnelee JD. Research priorities in vascular nursing. J Vasc Nurs. 1995;13:1-7.

272. Hauck Y, Kelly RG, Fenwick J. Research priorities for parenting and child health: a Delphi study. J Adv Nurs. 2007;59:129-39.

273. Haukoos JS, Mehta SD, Harvey L, Calderon Y, Rothman RE. Research priorities for human immunodeficiency virus and sexually transmitted infections surveillance, screening, and intervention in emergency departments: consensus-based recommendations. Acad Emerg Med. 2009;16:1096-102.

274. Hawarden A, Jinks C, Mahmood W, Bullock L, Blackburn S, Gwilym S, et al. Public priorities for osteoporosis and fracture research: results from a focus group study. Arch Osteoporos. 2020;15:89.

275. Hay A, Hall CW, Sealey M, Lobb EA, Breen LJ. Developing a practice-based research agenda for grief and bereavement care. Death Studies. 2021; 45:331-41.

276. Haynes SC, Rudov L, Nauman E, Hendryx L, Angove RSM, Carton T. Engaging stakeholders to develop a patient-centered research agenda: lessons learned from the research action for health network (REACHnet). Med Care. 2018;56 Suppl 1:S27-32.

277. Heal C, Roberts G. General practice research priority setting in Australia: informing a research agenda to deliver best patient care. Aust J Gen Pract. 2019;48:789-95.

278. Healy E, Brown SJ, Langan SM, Nicholls SG, Shams K, Reynolds NJ, et al. Identification of translational dermatology research priorities in the U.K.: results of an electronic Delphi exercise. Br J Dermatol. 2015;173:1191-8.

279. Healy P, Galvin S, Williamson PR, Treweek S, Whiting C, Maeso B, et al. Identifying trial recruitment uncertainties using a James Lind Alliance priority setting partnership - the PRioRiTy (Prioritising Recruitment in Randomised Trials) study. Trials. 2018;19:147.

280. Heartfield M. Research directions for specialist practice. Accid and Emerg Nurs. 2000;8:214-22.

281. Heazell AEP, Whitworth MK, Whitcombe J, Glover SW, Bevan C, Brewin J, et al. Research priorities for stillbirth: process overview and results from UK stillbirth priority setting partnership. Ultrasound Obstet Gynecol. 2015;46:641-7.

282. Helm PC, Körten M-A, Abdul-Khaliq H, Asfour B, Baumgartner H, Breithardt G, et al. Three parties, one direction: research priorities in adults with congenital heart disease. What do professionals, patients and relatives want to know? Int J Cardiol. 2016;207:220-9.

283. Hemmelgarn BR, Pannu N, Ahmed SB, Elliott MJ, Tam-Tham H, Lillie E, et al. Determining the research priorities for patients with chronic kidney disease not on dialysis. Nephrol Dial Transplant. 2017;32:847-54.

284. Henschke N, Maher CG, Refshauge KM, Das A, McAuley JH. Low back pain research priorities: a survey of primary care practitioners. BMC Fam Pract. 2007;8:40.

285. Henshaw H, Sharkey L, Crowe D, Ferguson M. Research priorities for mild-to-moderate hearing loss in adults. Lancet. 2015;386:2140-1.

286. Herbison P, Hay-Smith J, Paterson H, Ellis G, Wilson D. Research priorities in urinary incontinence: results from citizens' juries. BJOG. 2009;116:713-8.

287. Hibbs SP, Brunskill SJ, Donald GC, Saunders HD, Murphy MF. Setting priorities for research in blood donation and transfusion: outcome of the James Lind Alliance priority-setting partnership. Transfusion. 2019;59:574-81.

288. Hill B, Skouteris H, Boyle JA, Bailey C, Walker R, Thangaratinam S, et al. Health in preconception,

pregnancy and postpartum global alliance: international network pregnancy priorities for the prevention of maternal obesity and related pregnancy and long-term complications. J Clin Med. 2020;9:822.

289. Hill B, Skouteris H, Teede HJ, Bailey C, Baxter J-AB, Bergmeier HJ, et al. Health in preconception, pregnancy and postpartum global alliance: international network preconception research priorities for the prevention of maternal obesity and related pregnancy and long-term complications. J Clin Med. 2019;8:2119.

290. Hinckley J, Boyle E, Lombard D, Bartels-Tobin L. Towards a consumer-informed research agenda for aphasia: preliminary work. Disabil Rehabil. 2014;36:1042-50.

291. Hindin MJ, Christiansen CS, Ferguson BJ. Setting research priorities for adolescent sexual and reproductive health in low- and middle-income countries. Bull World Health Organ. 2013;91:10-8.

292. Hinds PS, Norville R, Anthony LK, Briscoe BW, Gattuso JS, Quargnenti A, Riggins MS, Walters LA, Wentz LJ, Scarbrough KE, Fairclough DC. Establishing pediatric cancer nursing research priorities: a Delphi study. J Pediatr Oncol Nurs. 1990;7:101-8.

293. Hinds PS, Quargnenti A, Olson MS, Gross J, Puckett P, Randall E, Gattuso JS, Wiedenhoffer D. The 1992 APON Delphi study to establish research priorities for pediatric oncology nursing. J Pediatr Oncol Nurs. 1994.11;20-7.

294. Hitch D, Lhuede K. Research priorities in mental health occupational therapy: a study of clinician perspectives. Aust Occup Ther J. 2015;62:326-32.

295. Ho A, Webster L, Bowen L, Creighton F, Findlay S, Gale C, et al. Research priorities for pregnancy hypertension: a UK priority setting partnership with the James Lind Alliance. BMJ Open. 2020;10:e036347.

296. Hodkinson PW, Wallis LA. Emergency medicine in the developing world: a Delphi study. Acad Emerg Med. 2010;17:765-74.

297. Hoffman JM, Keeling NJ, Forrest CB, Tubbs-Cooley HL, Moore E, Oehler E, et al. Priorities for pediatric patient safety research. Pediatrics. 2019;143:e20180496.

298. Hohenadel K, Pichora E, Marrett L, Bukvic D, Brown J, Harris S, et al. Priority issues in occupational cancer research: Ontario stakeholder perspectives. Chronic Dis Inj Can. 2011;31:147-51.

299. Hollin IL, Donaldson SH, Roman C, Aliaj E, Riva D, Boyle M, et al. Beyond the expected: Identifying broad research priorities of researchers and the cystic fibrosis community. J Cyst Fibros. 2019;18:375-7.

300. Hollis C, Sampson S, Simons L, Davies EB, Churchill R, Betton V, et al. Identifying research priorities for digital technology in mental health care: results of the James Lind Alliance priority setting partnership. Lancet Psychiatry. 2018;5:845-54.

301. Horne AW, Saunders PTK, Abokhrais IM, Hogg L. Top ten endometriosis research priorities in the UK and Ireland. Lancet. 2017;389:2191-2.

302. Hosseinzadeh T, Ghanbari A, Paryad E, Maghsoudi S, Pour Alizadeh M. Research priorities in evidence-based nursing cares: a Delphi technique. Journal of Health Sciences, 2019;9:9-16.

303. Howarth E, Vainre M, Humphrey A, Lombardo C, Hanafiah AN, Anderson JK, et al. Delphi study to identify key features of community-based child and adolescent mental health services in the East of England. BMJ Open. 2019;9:e022936.

304. Howell SJ, Pandit JJ, Rowbotham DJ. National Institute of Academic Anaesthesia research priority setting exercise. Br J Anaesth. 2012;108:42-52.

305. Hubbard G, Taylor C, Beeken B, Campbell A, Gracey J, Grimmett C, et al. Research priorities about stoma-related quality of life from the perspective of people with a stoma: a pilot survey. Health Expect. 2017;20:1421-7.

306. Hudson PL, Zordan R, Trauer T. Research priorities associated with family caregivers in palliative care: international perspectives. J Palliat Med. 2011;14:397-401.

307. Huglin L, Johnsen L, Marker A. Research priorities in performance technology: a Delphi study. Performance Improvement Quarterly. 2008;20:79-95.

308. Hunter A, Ross L, Gronlund T, Cooper S. UK research priorities for electronic cigarettes: a James Lind

Alliance priority setting partnership. Int J Environ Res Public Health. 2020;17:8500.

309. Iavicoli S. Research priorities in occupational health in Italy. Occup Environ Med. 2001;58:325-9.

310. Ingram JR, Abbott R, Ghazavi M, Alexandroff AB, McPhee M, Burton T, et al. The Hidradenitis Suppurativa priority setting partnership. Br J Dermatol. 2014;171:1422-7.

311. Ingram JSI, Wright HL, Foster L, Aldred T, Barling D, Benton TG, et al. Priority research questions for the UK food system. Food Sec. 2013;5:617-36.

312. Irvine C, Armstrong A, Nagata JM, Rollins N, Schaaf D, Doherty M, et al. Setting global research priorities in pediatric and adolescent HIV using the Child Health and Nutrition Research Initiative (CHNRI) methodology. J Acquir Immune Defic Syndr. 2018;78:S3-9.

313. Ismail D, McAteer H, Majeed-Ariss R, McPhee M, Griffiths CE, Young HS. Research priorities and identification of a health-service delivery model for psoriasis from the UK psoriasis priority setting partnership. Clin Exp Dermatol. 2021;46:276-85.

314. Jacobson S, Östlund P, Wallgren L, Österberg M, Tranæus S. Top ten research priorities for attention deficit/hyperactivity disorder treatment. Int J Technol Assess Health Care. 2016;32:152-9.

315. Jagnoor J, Bekker S, Chamania S, Potokar T, Ivers R. Identifying priority policy issues and health system research questions associated with recovery outcomes for burns survivors in India: a qualitative inquiry. BMJ Open. 2018;8:e020045.

316. Jagpal P, Saunders K, Plahe G, Russell S, Barnes N, Lowrie R, et al. Research priorities in healthcare of persons experiencing homelessness: outcomes of a national multi-disciplinary stakeholder discussion in the United Kingdom. Int J Equity Health. 2020;19:86.

317. Jang S-I, Cho K-H, Kim SJ, Lee K-S, Park E-C. Setting a health policy research agenda for controlling cancer burden in Korea. Cancer Res Treat. 2014;47:149-57.

318. Jaynes CL, Werman HA, White LJ. A blueprint for critical care transport research. Air Med J. 2013;32:30-5.

319. Jibb LA, Stacey D, Carley M, Davis A, Graham ID, Green E, et al. Research priorities for the pan-canadian oncology symptom triage and remote support practice guides: a modified nominal group consensus. Curr Oncol. 2019;26:173-82.

320. Department of Health & Social Care. Priorities for adult social work research. Results from the James Lind Alliance priority setting partnership for adult social work. 2018. https://www.jla.nihr.ac.uk/priority-setting-partnerships/adult-social-work/downloads/Adult-Social-Work-PSP-final-report.pdf. Accessed 26 July 2021.

321. National Institute for Health Research. Research priorities for alcohol-related liver disease. 2016. https://www.jla.nihr.ac.uk/priority-setting-partnerships/alcohol-related-liver-disease/downloads/Alcohol-related-Liver-Disease-PSP-final-report.pdf. Accessed 26 July 2021.

322. Autistica. Your questions: shaping future autism research. 2016. https://www.jla.nihr.ac.uk/priority-setting-partnerships/autism/downloads/Autism-PSP-final-report.pdf. Accessed 26 July 2021.

323. NIHR Oxford Biomedical Research Centre. 2016. Description of final workshop to set research priorities for bipolar. https://www.jla.nihr.ac.uk/priority-setting-partnerships/bipolar/downloads/Bipolar%20PSP%20Workshop%20report%20FINAL.pdf. Accessed 26 July 2021.

324. Coeliac UK. Coeliac UK's top 10 research priorities. 2018. https://www.jla.nihr.ac.uk/priority-setting-partnerships/coeliac-disease/downloads/research-priorities-web-brochure-2.pdf. Accessed 26 July 2021.

325. The Faculty of Sexual & Reproductive Healtchare. 2017. Collaboration, choice, care: the contraception priority setting partnership. https://www.jla.nihr.ac.uk/priority-setting-partnerships/contraception/downloads/Contraception-PSP-final-report.pdf. Accessed 26 July 2021.

326. MQ: Transforming Mental Health. 2016. Depression: asking the right questions. https://www.jla.nihr.ac.uk/priority-setting-partnerships/depression/downloads/Depression-PSP-final-report.pdf. Accessed 26 July 2021.

327. Crowe S, Tarpey M, Howe T, Regan S. 2016. Generating research questions from research priorities in early osteoarthritis of hip and knee. https://www.jla.nihr.ac.uk/priority-setting-partnerships/early-hip-and-knee-

osteoarthritis/downloads/Early%20Hip%20and%20Knee%20Osteoarthritis%20final%20workshop%20report%20including%20priorities.pdf. Accessed 26 July 2021.

328. Crowe S, Regan S. 2014. Description of a process and workshop to set research priorities in hip and knee replacement for osteoarthritis. https://www.jla.nihr.ac.uk/priority-setting-partnerships/hip-and-knee-replacement-for-osteoarthritis/downloads/Final-Workshop-Report.pdf. Accessed 26 July 2021.

329. Hyperhidrosis Research Network. 2019. https://www.jla.nihr.ac.uk/priority-setting-partnerships/Hyperhidrosis/downloads/Hyperhidrosis-PSP-Final-Report-Summary.pdf. Accessed 26 July 2021.

330. Lyme Disease Action. 2012. UK Lyme disease priority setting partnership – deciding the final priorities. https://www.jla.nihr.ac.uk/priority-setting-partnerships/lyme-disease/downloads/Lyme-Disease-PSP-Process-Report.pdf. Accessed 26 July 2021.

331. McPin Foundation. 2018. Research priorities for children and young people's mental health: interventions and services. https://www.jla.nihr.ac.uk/priority-setting-partnerships/Mental-health-in-children-and-young-people/downloads/Mental-Health-in-Children-and-Young-People-PSP-Main-Report.pdf. Accessed 26 July 2021.

332. Genetic Alliance UK. 2020. Mitochondrial disease priority setting partnership: setting research priorities with patients, carers and clinicians. https://www.jla.nihr.ac.uk/priority-setting-partnerships/mitochondrial-disease/downloads/mitochondrial-Disease-PSP-final-report.pdf. Accessed 26 July 2021.

333. Ontario Brain Institute. 2018. Community priorities for research on neurodevelopmental disorders. https://www.jla.nihr.ac.uk/priority-setting-partnerships/neurodevelopmental-disorders-canada/downloads/Neurodevelopmental-Disorders-Canada-Final-Report.pdf. Accessed 26 July 2021.

334. Neuro-Oncology Group. 2015. Top 10 priorities for clinical research in primary brain and spinal cord tumours. https://www.jla.nihr.ac.uk/priority-setting-partnerships/neuro-oncology/downloads/Neuro-Oncology-Group-Final-Report-June-2015.pdf. Accessed 26 July 2021.

335. Congenital Anaemia Network. 2018. Rare inherited anaemias priority setting partnership. https://www.jla.nihr.ac.uk/priority-setting-partnerships/rare-inherited-anaemias/downloads/Rare-Inherited-Anaemias-PSP-FINAL-REPORT.PDF. Accessed 26 July 2021.

336. British Scoliosis Research Foundation. 2017. Scoliosis priority setting partnership: final report. https://www.jla.nihr.ac.uk/priority-setting-partnerships/scoliosis/downloads/Scoliosis-PSP-final-report-including-all-54-verified-unanswered-questions.pdf. Accessed 26 July 2021.

337. Alberta Health Services. 2019. Seniors' health in Alberta: asking the right 1uestions of future research. https://www.jla.nihr.ac.uk/priority-setting-partnerships/seniors-health-in-alberta/Downloads/Alberta-Seniors-Health-PSP-Final-Report.pdf. Accessed 26 July 2021.

338. Johnson AP, Hanvey L, Baxter S, Heyland DK. Development of advance care planning research priorities: a call to action. J Palliat Care. 2013;29:99-106.

339. Jones DJ, Baldwin C, Lal S, Stanmore E, Farrer K, Connolly E, et al. Priority setting for adult malnutrition and nutritional screening in healthcare: a James Lind Alliance. J Hum Nutr Diet. 2020;33:274-83.

340. Jones J, Bhatt J, Avery J, Laupacis A, Cowan K, Basappa N, et al. The kidney cancer research priority-setting partnership: Identifying the top 10 research priorities as defined by patients, caregivers, and expert clinicians. Can Urol Assoc J. 2017;11:379-87.

341. Jones R, Lamont T, Haines A. Setting priorities for research and development in the NHS: a case study on the interface between primary and secondary care. BMJ. 1995;311:1076-80.

342. Jordans MJ, Luitel NP, Tomlinson M, Komproe IH. Setting priorities for mental health care in Nepal: a formative study. BMC Psychiatry. 2013;13:332.

343. Judge S, Bloch S, McDermott CJ. Communication change in ALS: engaging people living with ALS and their partners in future research. Disabil Rehabil Assist Technol. 2019;14:675-81.

344. Kakudate N, Yokoyama Y, Sumida F, Matsumoto Y, Riley JL, Gordan VV, et al. Practice-based research agenda priorities selected by patients: findings from a dental practice-based research network. Int Dent J. 2019;69:183-91.

345. Kathuria H, Detterbeck FC, Fathi JT, Fennig K, Gould MK, Jolicoeur DG, et al. Stakeholder research priorities for smoking cessation interventions within lung cancer screening programs. An official American Thoracic Society research statement. Am J Respir Crit Care Med. 2017;196:1202-12.

346. Kearney A, Daykin A, Shaw ARG, Lane AJ, Blazeby JM, Clarke M, et al. Identifying research priorities for effective retention strategies in clinical trials. Trials. 2017;18:406.

347. Kearney A, Williamson P, Young B, Bagley H, Gamble C, Denegri S, et al. Priorities for methodological research on patient and public involvement in clinical trials: a modified Delphi process. Health Expect. 2017;20:1401-10.

348. Keller H, Beck AM, Namasivayam A. Improving food and fluid intake for older adults living in long-term care: a research agenda. J Am Med Dir Assoc. 2015;16:93-100.

349. Kellum JA, Mehta RL, Levin A, Molitoris BA, Warnock DG, Shah SV, et al. Development of a clinical research agenda for acute kidney injury using an international, interdisciplinary, three-step modified Delphi process. Clin J Am Soc Nephrol. 2008;3:887-94.

350. Kelly S, Lafortune L, Hart N, Cowan K, Fenton M, Brayne C. Dementia priority setting partnership with the James Lind Alliance: using patient and public involvement and the evidence base to inform the research agenda. Age Ageing. 2015;44:985-93.

351. Khan N, Bacon SL, Khan S, Perlmutter S, Gerlinsky C, Dermer M, et al. Hypertension management research priorities from patients, caregivers, and healthcare providers: a report from the Hypertension Canada priority setting Partnership group. J Clin Hypertens. 2017;19:1063-9.

352. Khandelwal S, Avodé G, Baingana F, Conde B, Cruz M, Deva P, et al. Mental and neurological health research priorities setting in developing countries. Soc Psychiat Epidemiol. 2010;45:487-95.

353. Khazai Z, Van Brakel W, Essink D, Gillis T, Kasang C, Kuipers P, et al. Reviewing research priorities of the leprosy research initiative (LRI): a stakeholder's consultation. Leprosy. 2019;90:3-30.

354. Kilgore KL, Scherer M, Bobblitt R, Dettloff J, Dombrowski DM, Godbold N, et al. Neuroprosthesis consumers' forum: Consumer priorities for research directions. J Rehabil Res Dev. 2001;38:655-660.

355. Kim MJ, Oh E-G, Kim C-J, Yoo J-S, Ko I-S. Priorities for Nursing Research in Korea. J Nurs Scholarsh. 2002;34:307-12.

356. Kirkwood M, Wales A, Wilson A. A Delphi study to determine nursing research priorities in the North Glasgow University Hospitals NHS Trust and the corresponding evidence base. Health Info Libr J. 2003;20:53-8.

357. Klaprat NMD, Askin N, MacIntosh A, Brunton N, Hay JL, Yardley JE, et al. Filling gaps in type 1 diabetes and exercise research: a scoping review and priority-setting project. BMJ Open Diab Res Care. 2020;8:e001023.

358. Klein G, Gold LS, Sullivan SD, Buist DS, Ramsey S, Kreizenbeck K, et al. Prioritizing comparative effectiveness research for cancer diagnostics using a regional stakeholder approach. J Comp Eff Res. 2012;1:241-55.

359. Klotz R, Doerr-Harim C, Ahmed A, Tjaden C, Tarpey M, Diener MK, et al. Top ten research priorities for pancreatic cancer therapy. Lancet Oncology. 2020;21:e295-6.

360. Knight SR, Metcalfe L, O'Donoghue K, Ball ST, Beale A, Beale W, et al. Defining priorities for future research: results of the UK kidney transplant priority setting partnership. PLoS ONE. 2016;11:e0162136.

361. Knight SR, Pathak S, Christie A, Jones L, Rees J, Davies H, et al. Use of a modified Delphi approach to develop research priorities in HPB surgery across the United Kingdom. HPB. 2019;21:1446-52.

362. Knighton J, Carrougher GJ, Marvin JA, Bayley MnEW, Rutan RL, Weber B. Research priorities for burn nursing: report of the psychosocial issues group. J Burn Care Res. 1992;13:97-104.

363. Kouyoumdjian FG, Schuler A, McIsaac KE, Pivnick L, Matheson FI, Brown G, et al. Using a Delphi process to define priorities for prison health research in Canada. BMJ Open. 2016;6:e010125.

364. Kraiss LW, Conte MS, Geary RL, Kibbe M, Ozaki CK. Setting high-impact clinical research priorities for the Society for Vascular Surgery. J Vasc Surg. 2013;57:493-500.

365. Kriss JL, Grant GB, Moss WJ, Durrheim DN, Shefer A, Rota PA, et al. Research priorities for accelerating progress toward measles and rubella elimination identified by a cross-sectional web-based survey. Vaccine. 2019;37:5745-53.

366. Kühne F, Brütt AL, Otterbeck MJ, Weck F. Research priorities set by people with OCD and OCD researchers: do the commonalities outweigh the differences? Health Expectations. 2019; doi: 10.1111/hex.13005.

367. Kurubacak G. 2007. Identifying research priorities and needs in mobile learning technologies for distance education: a Delphi study. https://files.eric.ed.gov/fulltext/ED495997.pdf. Accessed 26 July 2021.

368. Lachat C, Nago E, Roberfroid D, Holdsworth M, Smit K, Kinabo J, et al. Developing a sustainable nutrition research agenda in sub-Saharan Africa - findings from the SUNRAY project. PLoS Med. 2014;11:e1001593.

369. Lai FY, Abbasciano RG, Tabberer B, Kumar T, Murphy GJ. Identifying research priorities in cardiac surgery: a report from the James Lind Alliance priority setting partnership in adult heart surgery. BMJ Open. 2020;10:e038001.

370. Lalloo D, Demou E, Smedley J, Madan I, Asanati K, Macdonald EB. Current research priorities for UK occupational physicians and occupational health researchers: a modified Delphi study. Occup Environ Med. 2018;75:830-6.

371. Lam JR, Liu B, Bhate R, Fenwick N, Reed K, Duffy JMN, et al. Research priorities for the future health of multiples and their families: the global twins and multiples priority setting partnership. Ultrasound Obstet Gynecol. 2019;54:715-21.

372. Lambert SD, Ould Brahim L, Morrison M, Girgis A, Yaffe M, Belzile E, et al. Priorities for caregiver research in cancer care: an international Delphi survey of caregivers, clinicians, managers, and researchers. Support Care Cancer. 2019;27:805-17.

373. Landes M, Lettow M van, Cataldo F, Chan AK, Barr BT, Harries AD, et al. Building a national direction for research in the prevention of mother to child transmission of HIV: results from a national prioritization initiative in Malawi. Health Res Policy Sys. 2013;11:40.

374. Lavigne M, Birken CS, Maguire JL, Straus S, Laupacis A. Priority setting in paediatric preventive care research. Arch Dis Child. 2017;102:748-53.

375. Law E, Starr JM, Connelly PJ. Dementia research - what do different public groups want? A survey by the Scottish Dementia Clinical Research Network. Dementia. 2013;12:23-8.

376. Lawn JE, Bahl R, Bergstrom S, Bhutta ZA, Darmstadt GL, Ellis M, et al. Setting research priorities to reduce almost one million deaths from birth asphyxia by 2015. PLoS Med. 2011;8:e1000389.

377. Layton A, Eady EA, Peat M, Whitehouse H, Levell N, Ridd M, et al. Identifying acne treatment uncertainties via a James Lind Alliance priority setting partnership. BMJ Open. 2015;5:e008085.

378. Le Reste J, Nabbe P, Lingner H, Kasuba Lazic D, Assenova R, Munoz M, et al. What research agenda could be generated from the European General Practice Research Network concept of multimorbidity in family practice? BMC Fam Pract. 2015;16:125.

379. Leach CR, Alfano CM, Potts J, Gallicchio L, Yabroff KR, Oeffinger KC, et al. Personalized cancer follow-up care pathways: a Delphi consensus of research priorities. J Natl Cancer Inst. 2020;112:1183-9.

380. Leake AR, Oculto T, Ramones E, Caagbay CR. Diabetes Bingo: research prioritization with the Filipino community. Hawaii Med J. 2010;69:237.

381. Lechelt LA, Rieger JM, Cowan K, Debenham BJ, Krewski B, Nayar S, et al. Top 10 research priorities in head and neck cancer: results of an Alberta priority setting partnership of patients, caregivers, family members, and clinicians. Head Neck. 2018;40:544-54.

382. Lee C, Nguyen AJ, Haroz E, Tol W, Aules Y, Bolton P. Identifying research priorities for psychosocial support programs in humanitarian settings. Glob Ment Health. 2019;6:e23.

383. Lee EH, Kim JS, Chung BY, Bok MS, Song BE, Kong SW, Lee EO. Research priorities of Korean oncology nurses. Cancer Nurs. 2003;26:387-91.

384. Lee CQ, Kouyoumdjian F, Christian J. Defining research priorities for bacterial sexually transmitted

infections in Canada. Can J Public Health. 2014;105:e86-90.

385. Lewandowski LA, Kositsky AM. Research priorities for critical care nursing: a study by the American Association of Critical-Care Nurses. Heart Lung. 1983;12:35-44.

386. Lewin G, Burton E, Sparrow P, Carroll M, Kendig H. Development of a community care research agenda for Australia: community care research agenda. Australas J Ageing. 2011;30:37-40.

387. Lim AK, Rhodes S, Cowan K, O'Hare A. Joint production of research priorities to improve the lives of those with childhood onset conditions that impair learning: the James Lind Alliance priority setting partnership for 'learning difficulties.' BMJ Open. 2019;9:e028780.

388. Lindeman CA. Delphi survey of priorities in clinical nursing research. Nursing Research. 19751;24:434-41.

389. Liossi C, Anderson AK, Howard RF, NIHR CRN-C CSG in Pain and Palliative Care. Development of research priorities in paediatric pain and palliative care. Br J Pain. 2017;11:9-15.

390. Liu X, Kolli S, McDonnell P, Patel A, Quinlan M, Skym K, et al. Patient priorities in herpes simplex keratitis. BMJ Open Ophth. 2019;4:e000177.

391. Loeb M, Brazil K, Durand P, Gordon M, Krueger P, Lewis D, et al. Identifying research priorities on infections in older adults: proceedings of an interdisciplinary workshop. BMC Geriatr. 2001;1:1.

392. Lõhmus A, Fridolin H, Leivits A, Tõnisson K, Rannap R. Prioritizing research gaps for national conservation management and policy: the managers' perspective in Estonia. Biodivers Conserv. 2019;28:2565-79.

393. Lomer MC, Hart AL, Verjee A, Daly A, Solomon J, Mclaughlin J. What are the dietary treatment research priorities for inflammatory bowel disease? A short report based on a priority setting partnership with the James Lind Alliance. J Hum Nutr Diet. 2017;30:709-13.

394. Longmuir PE, Tremblay MS. Top 10 research questions related to physical literacy. Res Q Exerc Sport. 2016;87:28-35.

395. Lopez V. Critical care nursing research priorities in Hong Kong. J Adv Nurs. 2003;43:578-87.

396. Lophatananon A, Tyndale-Biscoe S, Malcolm E, Rippon HJ, Holmes K, Firkins LA, et al. The James Lind Alliance approach to priority setting for prostate cancer research: an integrative methodology based on patient and clinician participation. BJU International. 2011;108:1040-3.

397. Lorenzon AR, Garcia D, Silva L, Oliveira CA de, Chehin MB, Marinho RM, et al. Research priorities in infertility and assisted reproductive technology treatments - a James Lind Alliance priority setting partnership with Brazilian patients. JBRA Assisted Reproduction. 2020. doi:10.5935/1518-0557.20190077.

398. Lough K, Hagen S, McClurg D, Pollock A. Shared research priorities for pessary use in women with prolapse: results from a James Lind Alliance priority setting partnership. BMJ Open. 2018;8:e021276.

399. Lovalekar M, Sharp MA, Billing DC, Drain JR, Nindl BC, Zambraski EJ. International consensus on military research priorities and gaps — survey results from the 4th International Congress on Soldiers' Physical Performance. J Sci Med Sport. 2018;21:1125-30.

400. Lowry SJ, Loggers ET, Bowles EJA, Wagner EH. Evidence gaps in advanced cancer care: community-based clinicians' perspectives and priorities for comparative effectiveness research. J Oncol Pract. 2012;8:28s-33s.

401. Ludwig Boltzmann Gesellschaft. 2016. "Tell us! In your opinion, what questions about mental illness should research take up?". https://www.redensiemit.org/files/Prozessdokumentation_CRIS.pdf . Accessed 26 July 2021.

402. Ludwig Boltzmann Gesellschaft. 2019."Tell us! In your opinion, what questions about accidental injuries should research take up?". https://storage.googleapis.com/target.instance-f52b6d31-c0f2-4636-a711-f4b84da502d5.euw1.beach.flownative.cloud/fccb73883edcd513a8cae1f7891401441070e1e8/Tell_us_Report2019_en.pdf. Accessed 26 July 2021.

403. Lyons KD, Radomski MV, Alfano CM, Finkelstein M, Sleight AG, Marshall TF, et al. Delphi study to determine rehabilitation research priorities for older adults with cancer. Arch Phys Med Rehabil. 2017;98:904-14.

404. Maassen EF, Regeer BJ, Bunders JFG, Regeer EJ, Kupka RW. A research agenda for bipolar disorder

developed from a patients' perspective. J Affect Disord. 2018;239:11-7.

405. Macbeth A, Tomlinson J, Messenger A, Moore-Millar K, Michaelides C, Shipman A, et al. Establishing and prioritizing research questions for the prevention, diagnosis and treatment of hair loss (excluding alopecia areata): the Hair Loss priority setting partnership. Br J Dermatol. 2018;178:535-40.

406. Macbeth AE, Tomlinson J, Messenger AG, Moore-Millar K, Michaelides C, Shipman AR, et al. Establishing and prioritizing research questions for the treatment of alopecia areata: the Alopecia Areata priority setting partnership. Br J Dermatol. 2017;176:1316-20.

407. MacDermid JC, Fess EE, Bell-Krotoski J, Cannon NM, Evans RB, Walsh W, et al. A research agenda for hand therapy. J Hand Ther. 20021;15:3-15.

408. Mackway-Jones K, Carley S. An international expert Delphi study to determine research needs in major incident management. Prehosp Disaster med. 2012;27:351-8.

409. Madi BC, Hussein J, Hounton S, D'Ambruoso L, Achadi E, Arhinful DK. Setting priorities for safe motherhood programme evaluation: a participatory process in three developing countries. Health Policy. 2007;83:94-104.

410. Madigan EA, Vanderboom C. Home health care nursing research priorities. Appl Nurs Res. 2005;18:221-5.

411. Makeen AM, Alanazi AMM, AlAhmari MD, Murriky AA, Alfaraj M, Al-Zalabani AH. Delphi consensus on research priorities in tobacco use and substance abuse in Saudi Arabia. J Ethn Subst Abuse. 2020; doi: 10.1080/15332640.2020.1845900.

412. Malcolm C, Forbat L, Knighting K, Kearney N. Exploring the experiences and perspectives of families using a children's hospice and professionals providing hospice care to identify future research priorities for children's hospice care. Palliat Med. 2008;22:921-8.

413. Malcolm C, Knighting K, Forbat L, Kearney N. Prioritisation of future research topics for children's hospice care by its key stakeholders: a Delphi study. Palliat Med. 2009;23:398-405.

414. Mamaril M, Ross J, Poole EL, Brady JM, Clifford T. ASPAN's Delphi Study on National Research: priorities for perianesthesia nurses in the United States. J Perianesth Nurs. 2009;24:4-13.

415. Manikam L, Shah R, Reed K, Santini G, Lakhanpaul M. Using a co-production prioritization exercise involving South Asian children, young people and their families to identify health priorities requiring further research and public awareness. Health Expect. 2017;20:852-61.

416. Manning JC, Hemingway P, Redsell SA. Survived so what? Identifying priorities for research with children and families post-paediatric intensive care unit. Nurs Crit Care. 2018;23:68-74.

417. Manns B, Hemmelgarn B, Lillie E, Dip SCPG, Cyr A, Gladish M, et al. Setting research priorities for patients on or nearing dialysis. Clin J Am Soc Nephrol. 2014;9:1813-21.

418. Martin MJ, Holcomb JB, Polk T, Hannon M, Eastridge B, Malik SZ, et al. The "Top 10" research and development priorities for battlefield surgical care: results from the Committee on Surgical Combat Casualty Care research gap analysis. J Trauma Acute Care Surg. 2019;87:S14-21.

419. Martins JPN, Karle BM, Heguy JM. Needs assessment for cooperative extension dairy programs in California. J Dairy Sci. 2019;102:7597-607.

420. Marvin JA, Carrougher G, Bayley B, Weber B, Knighton J, Rutan R. Burn nursing Delphi study: setting research priorities. J Burn Care Rehabil. 1991;12:190-7.

421. Mathews JA, Kalson NS, Tarrant PM, Toms AD, Revision Knee Replacement priority setting partnership steering group. Top ten research priorities for problematic knee arthroplasty: a priority setting partnership led by the British Association for Surgery of the Knee and the James Lind Alliance. Bone Joint J. 2020;102-B:1176-82.

422. Mathioudakis AG, Custovic A, Deschildre A, Ducharme FM, Kalayci O, Murray C, et al. Research priorities in pediatric asthma: results of a global survey of multiple stakeholder groups by the Pediatric Asthma in Real Life (PeARL) think tank. J Allergy Clin Immunol Pract. 2020;8:1953-1960.e9.

423. Matthews E, Collins CT, Ellison V, Hussey L, Slade J, Keir A, et al. Top 10 research priorities for human milk banking and use of donor human milk: a partnership between parents and clinicians. J Paediatr Child Health. 2020;56:770-6.

424. Mc Conalogue D, Kinn S, Mulligan JA, McNeil M. International consultation on long-term global health research priorities, research capacity and research uptake in developing countries. Health Res Pol Syst. 2017;15:1-6.

425. Mc Laughlin L, Spence S, Noyes J. Identifying integrated health services and social care research priorities in kidney disease in Wales: research prioritisation exercise. BMJ Open. 2020;10:e036872.

426. Mc Sharry J, Fredrix M, Hynes L, Byrne M. Prioritising target behaviours for research in diabetes: Using the nominal group technique to achieve consensus from key stakeholders. Res Involv Engagem. 2016;2:14.

427. McAllister M, Munday J, Taikato M, Waterhouse B, Dunn PK. Determining mental health research priorities in a Queensland region: an inclusive and iterative approach with mental health service clinicians, consumers and carers. Advances in Mental Health. 2012;10:268-76.

428. McCarthy G, Savage E, Lehane E. Research priorities for nursing and midwifery in Southern Ireland. Int Nurs Rev. 2006;53:123-8.

429. McDonald L, Hitzig SL, Pillemer KA, Lachs MS, Beaulieu M, Brownell P, et al. Developing a research agenda on resident-to-resident aggression: recommendations from a consensus conference. J Elder Abuse Negl. 2015;27:146-67.

430. McDonough S, McKenna H, Keeney S, Hasson F, Ward M, Kelly G, Lagan K, Duffy O. 2011. A Delphi study to identify research priorities for the therapy professions in Northern Ireland - executive summary report. https://pure.ulster.ac.uk/ws/files/11374687/NI_Delphi_Study_Executive_Summary.pdf. Accessed 26 July 2021.

431. Mcilfatrick SJ, Keeney S. Identifying cancer nursing research priorities using the Delphi technique. J Adv Nurs. 2003;42:629-36.

432. Mcintyre S, Novak I, Cusick A. Consensus research priorities for cerebral palsy: a Delphi survey of consumers, researchers, and clinicians. Dev Med Child Neurol. 2010;52:270-5.

433. McLay SV, McCutcheon D, Arendts G, Macdonald SP, Fatovich DM. Patient perspectives on priorities for emergency medicine research: the PERSPEX study. Emerg Med Australas. 2018;30:228-35.

434. McKeen DM, Banfield JC, McIsaac DI, McVicar J, McGavin C, et al. Top ten priorities for anesthesia and perioperative research: a report from the Canadian Anesthesia Research priority setting partnership. J Can Anesth. 2020;67:641-54.

435. McMullen CK, Safford MM, Bosworth HB, Phansalkar S, Leong A, Fagan MB, et al. Patient-centered priorities for improving medication management and adherence. Patient Educ Couns. 2015;98:102-10

436. McNair AGK, Heywood N, Tiernan J, Verjee A, Bach SP, Fearnhead NS, et al. A national patient and public colorectal research agenda: integration of consumer perspectives in bowel disease through early consultation. Colorectal Dis. 2017;19:O75-85.

437. McPherson AC, Ball GDC, Maltais DB, Swift JA, Cairney J, Knibbe TJ, et al. A call to action: setting the research agenda for addressing obesity and weight-related topics in children with physical disabilities. Childhood Obesity. 2016;12:59-69.

438. Medlow S, Patterson P. Determining research priorities for adolescent and young adult cancer in Australia: AYA research priorites. Eur J Cancer Care. 2015;24:590-9.

439. Melnick ER, Probst MA, Schoenfeld E, Collins SP, Breslin M, Walsh C, et al. Development and testing of shared decision making interventions for use in emergency care: a research agenda. Acad Emerg Med. 2016;23:1346-53.

440. Menozzi D, Kostov K, Sogari G, Arpaia S, Moyankova D, Mora C. A stakeholder engagement approach for identifying future research directions in the evaluation of current and emerging applications of GMOs. Bio-based and Applied Economics. 2017;18:57-79.

441. Mercer K, Baskerville N, Burns CM, Chang F, Giangregorio L, Tomasson Goodwin J, et al. Using a collaborative research approach to develop an interdisciplinary research agenda for the study of mobile health interventions for older adults. JMIR mHealth uHealth. 2015;3:e11.

442. Mickute G, Staley K, Delaney H, Gardiner O, Hunter A, Keen R, et al. Rare musculoskeletal diseases in adults: a research priority setting partnership with the James Lind Alliance. Orphanet J Rare Dis.

2020;15:117.

443. Mikton CR, Tanaka M, Tomlinson M, Streiner DL, Tonmyr L, Lee BX, et al. Global research priorities for interpersonal violence prevention: a modified Delphi study. Bull World Health Organ. 2017;95:36-48.

444. Miles-Tapping C, Dyck A, Brunham S, Simpson E, Barber L. Canadian therapists' priorities for clinical research: a Delphi study. Physical Therapy. 1990;70:448-54.

445. Misener TR, Watkins JG, Ossege J. Public health nursing research priorities: a collaborative Delphi study. Public Health Nurs. 1994;11:66-74.

446. Moerchen VA, Lundeen H, Dole RL. Educational research priorities for pediatric physical therapy: a consensus study. Pediatr Phys Ther. 2020;32:60-9.

447. Mollan S, Hemmings K, Herd CP, Denton A, Williamson S, Sinclair AJ. What are the research priorities for idiopathic intracranial hypertension? A priority setting partnership between patients and healthcare professionals. BMJ Open. 2019;9:e026573.

448. Monterosso L, Ross-Adjie G, Keeney S. Developing a research agenda for nursing and midwifery: a modified Delphi study. Contemp Nurse. 2015;51:83-95.

449. Monterosso L. Priorities for paediatric cancer nursing research in Western Australia: a Delphi study. Contemp Nurse. 2001;11:142-52.

450. Moorcraft SY, Sangha A, Peckitt C, Sanchez R, Lee M, Pattison N, et al. Does cancer research focus on areas of importance to patients? ecancer. 2016;10. doi:10.3332/ecancer.2016.ed51.

451. Moran L, Spencer L, Russell D, Hull M, Robertson S, Varcoe T, et al. Research priorities for fertility and conception research as identified by multidisciplinary health care practitioners and researchers. Nutrients. 2016;8:35.

452. Moreno-Casbas T, Martin-Arribas C, Orts-Cortes I, Comet-Cortes P. Identification of priorities for nursing research in Spain: a Delphi study. J Adv Nurs. 2001;35:857-63.

453. Morley JE, Caplan G, Cesari M, Dong B, Flaherty JH, Grossberg GT, et al. International survey of nursing home research priorities. J Am Med Dir Assoc. 2014;15:309-12.

454. Morof DF, Kerber K, Tomczyk B, Lawn J, Blanton C, Sami S, et al. Neonatal survival in complex humanitarian emergencies: setting an evidence-based research agenda. Confl Health. 2014;8:8.

455. Morris C, Simkiss D, Busk M, Morris M, Allard A, Denness J, et al. Setting research priorities to improve the health of children and young people with neurodisability: a British Academy of childhood disability-James Lind Alliance research priority setting partnership. BMJ Open. 2015;5:e006233-e006233.

456. Morris M, Crank H, Loosemore M, Stevinson C. Identification of research priorities in exercise oncology: a consensus study. J Cancer. 2020;11:2702-7.

457. Morris RL, Stocks SJ, Alam R, Taylor S, Rolfe C, Glover SW, et al. Identifying primary care patient safety research priorities in the UK: a James Lind Alliance priority setting partnership. BMJ Open. 2018;8:e020870.

458. Morton C, Muller S, Bucknall M, Gilbert K, Mallen CD, Hider SL. Examining management and research priorities in patients with polymyalgia rheumatica: a primary care questionnaire survey. Clin Rheumatol. 2019;38:1767-72.

459. Mosavel M, Simon C, van Stade D, Buchbinder M. Community-based participatory research (CBPR) in South Africa: Engaging multiple constituents to shape the research question. Soc Sci Med. 2005;61:2577-87.

460. Murphy A, Cowman S. Research priorities of oncology nurses in the Republic of Ireland. Cancer Nurs. 2006;29:283-90.

461. Murta FR, Waxman J, Skilton A, Wickwar S, Bonstein K, Cable R, et al. The first UK national blepharospasm patient and public involvement day; identifying priorities. Orbit. 2020;39:233-40.

462. Nabeiei P, Amini M, Ghanavati S, Marhamati S. Research priorities in medical education at Shiraz University of Medical Sciences: categories and subcategories in the Iranian context. J Adv Med Educ Prof. 2016;4:26.

463. Nagarajan G, Arumugam E, Tharion G, Bhattacharji S. Perceptions of patients with spinal cord injury on future research in South India. Social Care Neurodisability. 2012;3:20-6.

464. Nagata JM, Ferguson BJ, Ross DA. Research priorities for eight areas of adolescent health in low- and middle-income countries. J Adolesc Health. 2016;59:50-60.

465. Nalder EJ, Zabjek K, Dawson DR, Bottari CL, Gagnon I, McFadyen BJ, et al. Research priorities for optimizing long-term community integration after brain injury. Can J Neurol Sci. 2018;45:643-51.

466. Nast I, Tal A, Schmid S, Schoeb V, Rau B, Barbero M, et al. Physiotherapy research priorities in Switzerland: views of the various stakeholders. Physiother Res Int. 2016;21:137-46.

467. Nathens AB, Cook CH, Machiedo G, Moore EE, Namias N, Nwariaku F. Defining the research agenda for surgical infection: a consensus of experts using the Delphi approach. Surgical Infections. 2006;7:101-10.

468. National Institute for Health Research Global Health Research Unit on Global Surgery, Nepogodiev D, Moore R, Biccard B, Rayne S, Costas-Chavarri A, et al. Prioritizing research for patients requiring surgery in low- and middle-income countries. Br J Surg. 2019;106:e113-20.

469. Nelson ML, McKellar KA, Munce S, Kelloway L, Hans PK, Fortin M, et al. Addressing the evidence gap in stroke rehabilitation for complex patients: a preliminary research agenda. Arch Phys Med Rehabil. 2018;99:1232-41.

470. Nevill RJ, Hall PM, Beale J. Forest health research needs in British Columbia. The Forestry Chronicle. 1995;71:489-96.

471. Newell G, Acheampong P, Worzala E. Property research priorities in Australia. Pacific Rim Property Research Journal. 2002;8:127-39.

472. Nierse CJ, Abma TA, Horemans AMC, van Engelen BGM. Research priorities of patients with neuromuscular disease. Disabil Rehabil. 2013;35:405-12.

473. Nierse CJ, Abma TA. Developing voice and empowerment: the first step towards a broad consultation in research agenda setting. J Intellect Disabil Res. 2011;55:411-21.

474. Nixon NA, Simmons C, Lemieux J, Verma S. Research priorities in metastatic breast cancer: a James Lind Alliance priority setting partnership. Breast J. 2020;26:488-93.

475. Noroozi M, Larijani B, Nedjat S, Aramesh K, Salari P. Priority setting for research in the field of medical ethics in the Islamic Republic of Iran: a Delphi study. East Mediterr Health J. 2020;26:531-8.

476. Obeid N, McVey G, Seale E, Preskow W, Norris ML. Cocreating research priorities for anorexia nervosa: the Canadian eating disorder priority setting partnership. Int J Eat Disord. 2020;53:662-72.

477. O'Brien KK, Ibáñez-Carrasco F, Solomon P, Harding R, Brown D, Ahluwalia P, et al. Research priorities for rehabilitation and aging with HIV: a framework from the Canada-International HIV and Rehabilitation Research Collaborative (CIHRRC). AIDS Res Ther. 2020;17:21.

478. Oczkowski SJW, Au S, des Ordons AR, Gill M, Potestio ML, Smith O, et al. A modified Delphi process to identify clinical and research priorities in patient and family centred critical care. J Crit Care. 2017;42:243-7.

479. O'Donnell SM, Carison A, Adams J, Long E, Babl FE. Delphi assessment of audit and research priorities in an emergency department. Emerg Med Australas. 2020;32:556-61.

480. O'Keefe BM, Kozak NB, O'Keefe BM, Kozak NB, Schuller R, O'Keefe BM, et al. Research priorities in augmentative and alternative communication as identified by people who use AAC and their facilitators. Augment Altern Commun. 2007;23:89-96.

481. Oldekop JA, Fontana LB, Grugel J, Roughton N, Adu-Ampong EA, Bird GK, et al. 100 key research questions for the post-2015 development agenda. Dev Policy Rev. 2016;34:55-82.

482. Oliver S, Uhm S, Duley L, Crowe S, David AL, James CP, et al. Top research priorities for preterm birth: results of a prioritisation partnership between people affected by preterm birth and healthcare professionals. BMC Pregnancy Childbirth. 2019;19:528.

483. O'Neill B, Aversa V, Rouleau K, Lazare K, Sullivan F, Persaud N. Identifying top 10 primary care research priorities from international stakeholders using a modified Delphi method. PLoS ONE. 2018;13:e0206096.

484. O'Regan M, Gethin G, O'Loughlin A, O'Connor G, Dineen S, Pandit A, et al. Public & patient involvement to

guide research in wound care in an Irish context. A round table report. J Tissue Viability. 2020;29:7-11.

485. Ota S, Cron RQ, Schanberg LE, O'Neil K, Mellins ED, Fuhlbrigge RC, et al. Research priorities in pediatric rheumatology: the Childhood Arthritis and Rheumatology Research Alliance (CARRA) consensus. Pediatr Rheumatol. 2008;6:5.

486. Owens C, Ley A, Aitken P. Do different stakeholder groups share mental health research priorities? A four-arm Delphi study. Health Expect. 2008;11:418-31.

487. Padfield R, Waldron S, Drew S, Papargyropoulou E, Kumaran S, Page S, et al. Research agendas for the sustainable management of tropical peatland in Malaysia. Envir Conserv. 2015;42:73-83.

488. Palermo C, King O, Brock T, Brown T, Crampton P, Hall H, et al. Setting priorities for health education research: a mixed methods study. Medical Teacher. 2019;41:1029-38.

489. Panchal AR, Cash RE, Crowe RP, Coute R, Way D, Aufderheide T, et al. Delphi analysis of science gaps in the 2015 American Heart Association Cardiac Arrest Guidelines. J Am Heart Assoc. 2018;7:e008571.

490. Parker SG, Corner L, Laing K, Nestor G, Craig D, Collerton J, et al. Priorities for research in multiple conditions in later life (multi-morbidity): findings from a James Lind Alliance priority setting partnership. Age Ageing. 2019;48:401-6.

491. Parlour R, Slater P. Developing nursing and midwifery research priorities: a health service executive (HSE) North West study. Worldviews Evid Based Nurs. 2014;11:200-8.

492. Parsons S, Thomson W, Cresswell K, Starling B, McDonagh JE. What do young people with rheumatic disease believe to be important to research about their condition? A UK-wide study. Pediatr Rheumatol. 2017;15:53.

493. Paskins Z, Jinks C, Mahmood W, Jayakumar P, Sangan CB, Belcher J, et al. Public priorities for osteoporosis and fracture research: results from a general population survey. Arch Osteoporos. 2017;12:45.

494. Patel K, Moorthy D, Chan JA, Concannon TW, Ratichek SJ, Chung M, et al. High priority future research needs for obstructive sleep apnea diagnosis and treatment. J Clin Sleep Med. 2013;09:395-402.

495. Paterson DL. Determining research priorities for clinician-initiated trials in infectious diseases. Med J Aust. 2013;198:270-2.

496. Paul CL, Sanson-Fisher R, Douglas HE, Clinton-Mcharg T, Williamson A, Barker D. Cutting the research pie: a value-weighting approach to explore perceptions about psychosocial research priorities for adults with haematological cancers. Eur J Cancer Care. 2011;20:345-53.

497. Payne JB, Dance KV, Farone M, Phan A, Ho CD, Gutierrez M, et al. Patient and caregiver perceptions of lymphoma care and research opportunities: a qualitative study. Cancer. 2019;125:4096-104.

498. Peeks F, Boonstra WF, Baere L, Carøe C, Casswall T, Cohen D, et al. Research priorities for liver glycogen storage disease: an international priority setting partnership with the James Lind Alliance. J Inher Metab Dis. 2020;43:279-89.

499. Pellicano E, Dinsmore A, Charman T. What should autism research focus upon? Community views and priorities from the United Kingdom. Autism. 2014;18:756-70.

500. Pérez-Sales P, Witcombe N, Oyague DO. Rehabilitation of torture survivors and prevention of torture: priorities for research through a modified Delphi Study. Torture J. 2017;27:46.

501. Perkins P, Barclay S, Booth S. What are patients' priorities for palliative care research? Focus group study. Palliat Med. 2007;21:219-25.

502. Perkins P, Booth S, Vowler SL, Barclay S. What are patients' priorities for palliative care research? A questionnaire study. Palliat Med. 2008;22:7-12.

503. Perry DC, Wright JG, Cooke S, Roposch A, Gaston MS, Nicolaou N, et al. A consensus exercise identifying priorities for research into clinical effectiveness among children's orthopaedic surgeons in the United Kingdom. Bone Joint J. 2018;100-B:680-4.

504. Phillips R, Williams D, Bowen D, Morris D, Grant A, Pell B, et al. Reaching a consensus on research priorities for supporting women with autoimmune rheumatic diseases during pre-conception, pregnancy and early parenting: a Nominal Group Technique exercise with lay and professional stakeholders. Wellcome

Open Res. 2018;3:75.

505. Piano MR, Artinian NT, DeVon HA, Pressler ST, Hickey KT, Chyun DA. Cardiovascular nursing science priorities: a statement from the American Heart Association Council on cardiovascular and stroke nursing. J Cardiovasc Nurs. 2018;33:E11-20.

506. Pillay P, Thorburn J. Research priorities in the education of visually impaired students in New Zealand. J Vis Impair Blind. 1997;91:87-8.

507. Pinyerd BJ, Blair JM, Chavez R, Stout-Shaffer S. Setting a research agenda to promote nursing research. Clin Nurs Res. 1993;2:232-9.

508. Pitman A, Khrisna Putri A, Kennedy N, De Souza T, King M, Osborn D. Priorities for the development and evaluation of support after suicide bereavement in the UK: results of a discussion group. Bereave Care. 2016;35:109-16.

509. Plint AC, Stang AS, Calder LA. Establishing research priorities for patient safety in emergency medicine: a multidisciplinary consensus panel. Int J Emerg Med. 2015;8:1.

510. Pollard C, Pollard R. Research priorities in educational technology: a Delphi study. J Research Technology in Education. 2004;37:145-60.

511. Pollock A, St George B, Fenton M, Firkins L. Top 10 research priorities relating to life after stroke – consensus from stroke survivors, caregivers, and health professionals. Int J Stroke. 2014;9:313-20.

512. Porter J, Charlton K, Tapsell L, Truby H. Using the Delphi process to identify priorities for dietetic research in Australia 2020-2030. Nutr Diet. 2020;77:437-43.

513. Poulin P, Shergill Y, Romanow H, Busse JW, Chambers CT, Cooper L, et al. Researching what matters to improve chronic pain care in Canada: a priority-setting partnership process to support patient-oriented research. Can J Pain. 2018;2:191-204.

514. Powell RA, Harding R, Namisango E, Katabira E, Gwyther L, Radbruch L, et al. Palliative care research in Africa: consensus building for a prioritized agenda. J Pain Symptom Manage. 2014;47:315-24.

515. Prior M, Bagness C, Brewin J, Coomarasamy A, Easthope L, Hepworth-Jones B, et al. Priorities for research in miscarriage: a priority setting partnership between people affected by miscarriage and professionals following the James Lind Alliance methodology. BMJ Open. 2017;7:e016571.

516. Prudhon C, Maclaine A, Hall A, Benelli P, Harrigan P, Frize J. Research priorities for improving infant and young child feeding in humanitarian emergencies. BMC Nutr. 2016;2:27.

517. Purkey E, Htoo SN, Whelan R, Mhote NPP, Davison CM. Creating a locally driven research agenda for the ethnic minorities of Eastern Myanmar. Health Res Policy Sys. 2019;17:64.

518. Rafie CL, Zimmerman EB, Moser DE, Cook S, Zarghami F. A lung cancer research agenda that reflects the diverse perspectives of community stakeholders: process and outcomes of the SEED method. Res Involv Engagem. 2019;5:3.

519. Rafie E, Karamali M, Bahadori M, Yazdanian M, Ravangard R. Needs assessment and research priorities in the oral and dental health with health promotion approach in Iran. J Educ Health Promot. 2019;8:93.

520. Rahman SA, Otim ME, Almarzouqi A, Rahman S. Setting priorities in childhood cancer in low income countries using Nominal Group Technique: experience from an international childhood cancer forum exercise in Bangladesh. Asian Pac J Cancer Prev. 2019;20:97-103.

521. Ramelet AS, Gill F. A Delphi study on national PICU nursing research priorities in Australia and New Zealand. Aust Crit Care. 2012;25:41-57.

522. Ramirez AG, Chalela P, Gallion KJ, Green LW, Ottoson J. *Salud America*! Developing a national Latino childhood obesity research agenda. Health Educ Behav. 2011;38:251-60.

523. Rangan A, Upadhaya S, Regan S, Toye F, Rees JL. Research priorities for shoulder surgery: results of the 2015 James Lind Alliance patient and clinician priority setting partnership. BMJ Open. 2016;6:e010412.

524. Rankin G, Rushton A, Olver P, Moore A. Chartered Society of Physiotherapy's identification of national research priorities for physiotherapy using a modified Delphi technique. Physiotherapy. 2012;98:260-72.

525. Rankin G, Summers R, Cowan K, Barker K, Button K, Carroll SP, et al. Identifying priorities for

physiotherapy research in the UK: the James Lind Alliance physiotherapy priority setting partnership. Physiotherapy. 2020;107:161-8.

526. Rankin M, Borah GL, Kosa E. Research priorities and concerns of plastic surgical nurses. Plast Surg Nurs. 1998;18:86.
527. Rankin NM, Butow PN, Price MA, Evans A. Views of psycho-oncology health professionals on priority psycho-oncology research questions. Support Care Cancer. 2011;19:1133-41.
528. Ranse J, Hutton A, Jeeawody B, Wilson R. What are the research needs for the field of disaster nursing? An international Delphi study. Prehosp Disaster Med. 2014;29:448-54.
529. Ranson K, Law TJ, Bennett S. Establishing health systems financing research priorities in developing countries using a participatory methodology. Soc Sci Med. 2010;70:1933-42.
530. Ranson MK, Chopra M, Atkins S, Dal Poz MR, Bennett S. Priorities for research into human resources for health in low- and middle-income countries. Bull World Health Organ. 2010;88:435-43.
531. Raskin MS. A Delphi study in field instruction: identification of issues and research priorities by experts. Clin Superv. 1989;6:29-46.
532. Ravaghi H, Sajadi HS. Research priorities in the field of patient safety in Iran: results of a Delphi study. J Patient Saf. 2019;15:166-71.
533. Ravindran TKS, Seshadri T. A health equity research agenda for India: results of a consultative exercise. Health Res Policy Sys. 2018;16:94.
534. Rayner J, Fetherstonhaugh D, Cowen S. Research priorities in residential aged care services: a statewide survey. Australas J Ageing. 2020;39:40-7.
535. Reay H, Arulkumaran N, Brett SJ. Priorities for future intensive care research in the UK: results of a James Lind Alliance priority setting partnership. J Intensive Care Soc. 2014;15:288-96.
536. Rees SE, Chadha R, Donovan LE, Guitard ALT, Koppula S, Laupacis A, et al. Engaging patients and clinicians in establishing research priorities for gestational diabetes mellitus. Can J Diabetes. 2017;41:156-63.
537. Rehfuess EA, Durão S, Kyamanywa P, Meerpohl JJ, Young T, Rohwer A, et al. An approach for setting evidence-based and stakeholder-informed research priorities in low- and middle-income countries. Bull World Health Organ. 2016;94:297-305.
538. Reid J, O'Reilly R, Beale B, Gillies D, Connell T. Research priorities of NSW midwives. Women Birth. 2007;20:57-63.
539. Reis RS, Kelly CM, Parra DC, Barros M, Gomes G, Malta D, et al. Developing a research agenda for promoting physical activity in Brazil through environmental and policy change. Rev Panam Salud Publica. 2012;32:93-100.
540. Restall GJ, Carnochan TN, Roger KS, Sullivan TM, Etcheverry EJ, Roddy P. Collaborative priority setting for human immunodeficiency virus rehabilitation research: a case report. Can J Occup Ther. 2016;83:7-13.
541. Reupert A, Drost LM, Marston N, Stavnes K, Van Loon LMA, Charles G, et al. Developing a shared research agenda for working with families where a parent has a mental illness. Child Youth Serv. 2016;37:194-209.
542. Richardson A, Addington-Hall J, Stark D, Foster C, Amir Z, Sharpe M. 2009. Determining research priorities for cancer survivorship: consultation and evidence review. https://eprints.soton.ac.uk/154477/2/Technical_Appendices_2_Consultation_FINAL_9-3-2010.pdf. Accessed 26 July 2021.
543. Rideout C, Gil R, Browne R, Calhoon C, Rey M, Gourevitch M, et al. Using the Delphi and snow card techniques to build consensus among diverse community and academic stakeholders. Prog Community Health Partnersh. 2013;7:331-9.
544. Robert G, Milne R. A Delphi study to establish national cost-effectiveness research priorities for positron emission tomography. Eur J Radiol. 1999;30:54-60.
545. Robotin M, Holliday C, Bensoussan A. Defining research priorities in complementary medicine in oncology.

Complement Ther Med. 2012;20:345-52.

546. Robotin MC, Jones SC, Biankin AV, Waters L, Iverson D, Gooden H, et al. Defining research priorities for pancreatic cancer in Australia: results of a consensus development process. Cancer Causes Control. 2010;21:729-36.

547. Rodger M, Hills J, Kristjanson L. A Delphi study on research priorities for emergency nurses in Western Australia. J Emerg Nurs. 2004;30:117-25.

548. Rodgers M, Booth A, Norman G, Sowden A. Research priorities relating to the debate on assisted dying: what do we still need to know? Results of a modified Delphi technique. BMJ Open. 2016;6:e012213.

549. Rodríguez ACT, Binda E, Quintero JM, Garcia H, Gómez B, Soto C, Martínez S, Clerici N. Answering the right questions. Addressing biodiversity conservation in post-conflict Colombia. Environmental Science & Policy. 2020;104:82-7.

550. Rollins N, Chanza H, Chimbwandira F, Eliya M, Nyasulu I, Thom E, et al. Prioritizing the PMTCT implementation research agenda in 3 African countries: integrating and scaling up PMTCT through implementation research (INSPIRE). J Acquir Immune Defic Syndr. 2014;67:6.

551. Roney L, McKenna C. Determining the education and research priorities in pediatric trauma nursing: a Delphi Study. J Trauma Nurs. 2018;25:290-7.

552. Ropka ME, Guterbock TM, Krebs LU, Murphy-Ende K, Stetz KM, Summers BL, et al. Year 2000 Oncology Nursing Society research priorities survey. Oncol Nurs Forum. 2002;29:481-91.

553. Rose D, Fleischman P, Wykes T. What are mental health service users' priorities for research in the UK? J Ment Health. 2008;17:520-30.

554. Rossi SH, Blick C, Handforth C, Brown JE, Stewart GD. Essential research priorities in renal cancer: a modified Delphi consensus statement. Eur Urol Focus. 2020;6:991-8.

555. Rowat A, Lawrence M, Horsburgh D, Legg L, Smith LN. Stroke research questions: a nursing perspective. Br J Nurs. 2009;18:100-5.

556. Rowat A, Pollock A, St George B, Cowey E, Booth J, Lawrence M, et al. Top 10 research priorities relating to stroke nursing: a rigorous approach to establish a national nurse-led research agenda. J Adv Nurs. 2016;72:2831-43.

557. Rowbotham NJ, Smith S, Leighton PA, Rayner OC, Gathercole K, Elliott ZC, et al. The top 10 research priorities in cystic fibrosis developed by a partnership between people with CF and healthcare providers. Thorax. 2018;73:388-90.

558. Rowe F, Wormald R, Cable R, Acton M, Bonstein K, Bowen M, et al. The sight loss and vision priority setting partnership (SLV-PSP): overview and results of the research prioritisation survey process. BMJ Open. 2014;4:e004905-e004905.

559. Rubinstein SM, Bolton J, Webb AL, Hartvigsen J. The first research agenda for the chiropractic profession in Europe. Chiropr Man Therap. 2014;22:9.

560. Rubinstein TB, Ogbu EA, Rodriguez M, Waqar L, Woo JMP, Davis AM, et al. Prioritized agenda for mental health research in pediatric rheumatology from the Childhood Arthritis and Rheumatology Research Alliance Mental Health Workgroup. J Rheumatol. 2020;47:1687-95.

561. Rudan I, El Arifeen S, Bhutta ZA, Black RE, Brooks A, Chan KY, et al. Setting research priorities to reduce global mortality from childhood pneumonia by 2015. PLoS Med. 2011;8:e1001099.

562. Rudd MA, Beazley KF, Cooke SJ, Fleishman E, Lane DE, Mascia MB, et al. Generation of priority research questions to inform conservation policy and management at a national level: research questions to inform policy. Conserv Biol. 2011;25:476-84.

563. Rudd MA, Moore AFP, Rochberg D, Bianchi-Fossati L, Brown MA, D'Onofrio D, et al. Climate research priorities for policy-makers, practitioners, and scientists in Georgia, USA. Environ Manage. 2018;62:190-209.

564. Rudolph S, Hiscock H, Price A, Efron D, Sewell J, South M, et al. What research questions matter to Australian paediatricians? National Delphi study. J Paediatr Child Health. 2009;45:704-10.

565. Rushton A, Moore A. International identification of research priorities for postgraduate theses in musculoskeletal physiotherapy using a modified Delphi technique. Man Ther. 2010;15:142-8.

566. Rushton AB, Fawkes CA, Carnes D, Moore AP. A modified Delphi consensus study to identify UK osteopathic profession research priorities. Man Ther. 2014;19:445-52.

567. Rustøen T, Schjølberg TKr. Cancer nursing research priorities: a Norwegian perspective. Cancer Nurs. 2000;23:375-81.

568. Sadhra S. Occupational health research priorities in Malaysia: a Delphi study. Occup Environ Med. 2001;58:426-31.

569. Sage L, Russo ML, Byers PH, Demasi J, Morris SA, Puryear LN, et al. Setting a research agenda for vascular Ehlers-Danlos syndrome using a patient and stakeholder engagement model. J Vasc Surg. 2020;72:1436-1444.e2.

570. Sakashita A, Morita T, Kishino M, Aoyama M, Kizawa Y, Tsuneto S, et al. Which research questions are important for the bereaved families of palliative care cancer patients? A nationwide survey. J Pain Symptom Manage. 2018;55:379-86.

571. Salman RA-S, Kitchen N, Thomson J, Ganesan V, Mallucci C, Radatz M. Top ten research priorities for brain and spine cavernous malformations. Lancet Neurology. 2016;15:354-5.

572. Sandberg DE, Singer D, Bugajski B, Gebremariam A, Scerbak T, Dooley Maley KL, et al. Research priorities of people living with Turner syndrome. Am J Med Genet. 2019;181:13-21.

573. Sangvatanakul P, Hillege S, Lalor E, Levi C, Hill K, Middleton S. Setting stroke research priorities: the consumer perspective. J Vasc Nurs. 2010;28:121-31.

574. Santana M-J, Zelinsky S, Ahmed S, Doktorchik C, James M, Wilton S, et al. Patients, clinicians and researchers working together to improve cardiovascular health: a qualitative study of barriers and priorities for patient-oriented research. BMJ Open. 2020;10:e031187.

575. Saunders C, Crossing S. Towards meeting the research needs of Australian cancer consumers. BMC Res Notes. 2012;5:667.

576. Saunders C, Gooden H, Robotin M, Mumford J. As the bell tolls: a foundation study on pancreatic cancer consumer's research priorities. BMC Res Notes. 2009;2:179.

577. Sawford K, Dhand NK, Toribio J-AL, Taylor MR. The use of a modified Delphi approach to engage stakeholders in zoonotic disease research priority setting. BMC Public Health. 2014;14:182.

578. Sawin KJ, Lewin LC, Niederhauser VP, Brady MA, Jones D, Butz A, et al. A survey of NAPNAP members' clinical and professional research priorities. J Pediatr Health Care. 2012;26:5-15.

579. Schipper K, Abma TA. Coping, family and mastery: top priorities for social science research by patients with chronic kidney disease. Nephrol Dial Transplant. 2011;26:3189-95.

580. Schipper K, Dauwerse L, Hendrikx A, Leedekerken JW, Abma TA. Living with Parkinson's disease: priorities for research suggested by patients. Parkinsonism Relat Disord. 2014;20:862-6.

581. Schmidt K, Montgomery LA, Bruene D, Kenney M. Determining research priorities in pediatric nursing: a Delphi study. J Pediatr Nurs. 1997;12:201-7.

582. Schneider PJ, Evaniew N, McKay P, Ghert M. Moving forward through consensus: a modified Delphi approach to determine the top research priorities in orthopaedic oncology. Clin Orthop Relat Res. 2017;475:3044-55.

583. Schoenly L. Research priorities in correctional nursing practice: results of a three-round Delphi study. J Correct Health Care. 2015;21:400-7.

584. Schölvinck A-FM, de Graaff BMB, van den Beld MJ, Broerse JEW. Research in haematological cancers: what do patients in the Netherlands prioritise? Eur J Cancer Care. 2019;28:e12989.

585. Schölvinck A-FM, Pittens CACM, Broerse JEW. The research priorities of people with visual impairments in the Netherlands. J Vis Impair Blind. 2017;111:201-17.

586. Scott ES, Murphy LS, Warshawsky NE. Nursing administration research priorities: findings from a Delphi study. J Nurs Adm. 2016;46:238-44.

587. Scott K, Jessani N, Qiu M, Bennett S. Developing more participatory and accountable institutions for health: identifying health system research priorities for the Sustainable Development Goal-era. Health Policy Plan. 2018;33:975-87.

588. Seakins A, Dillon J. Exploring research themes in public engagement within a natural history museum: a modified Delphi approach. Int J Sci Educ B Commun Public Engagem. 2013;3:52-76.

589. Segelov E, Chan D, Lawrence B, Pavlakis N, Kennecke HF, Jackson C, et al. Identifying and prioritizing gaps in neuroendocrine tumor research: a modified delphi process with patients and health care providers to set the research action plan for the newly formed commonwealth neuroendocrine tumor collaboration. J Glob Oncol. 2017;3:380-8.

590. Selman L, Young T, Vermandere M, Stirling I, Leget C. Research priorities in spiritual care: an international survey of palliative care researchers and clinicians. J Pain Symptom Manage. 2014;48:518-31.

591. Selman LE, Brighton LJ, Sinclair S, Karvinen I, Egan R, Speck P, et al. Patients' and caregivers' needs, experiences, preferences and research priorities in spiritual care: a focus group study across nine countries. Palliat Med. 2018;32:216-30.

592. Setty K, Jiménez A, Willetts J, Leifels M, Bartram J. Global water, sanitation and hygiene research priorities and learning challenges under Sustainable Development Goal 6. Dev Policy Rev. 2020;38:64-84.

593. Sewell W, Steyn JN, Venter P, Mason RB. Governance of strategically relevant research in the wholesale and retail sector. Risk Governance and Control: Financial Markets and Institutions. 2016;6:60-8.

594. Shapiro S, Stephensen D, Camp C, Carroll L, Collins P, Elston D, et al. The top 10 research priorities in bleeding disorders: a James Lind Alliance priority setting partnership. Br J Haematol. 2019;186. doi:10.1111/bjh.15928.

595. Sharan P, Gallo C, Gureje O, Lamberte E, Mari JJ, Mazzotti G, et al. Mental health research priorities in low- and middle-income countries of Africa, Asia, Latin America and the Caribbean. Br J Psychiatry. 2009;195:354-63.

596. Sharma R, Buccioni M, Gaffey MF, Mansoor O, Scott H, Bhutta ZA, et al. Setting an implementation research agenda for Canadian investments in global maternal, newborn, child and adolescent health: a research prioritization exercise. CMAJ Open. 2017;5:E82-9.

597. Sharma R, Gaffey MF, Alderman H, Bassani DG, Bogard K, Darmstadt GL, et al. Prioritizing research for integrated implementation of early childhood development and maternal, newborn, child and adolescent health and nutrition platforms. J Glob Health. 2017;7:011002.

598. Sheehan WJ, Williams MA, Paskins Z, Costa ML, Fernandez MA, Gould J, et al. Research priorities for the management of broken bones of the upper limb in people over 50: a UK priority setting partnership with the James Lind Alliance. BMJ Open. 2019;9:e030028.

599. Sheikh A, Rudan I, Cresswell K, Dhingra-Kumar N, Tan ML, Häkkinen ML, et al. Agreeing on global research priorities for medication safety: an international prioritisation exercise. J Glob Health. 2019;9:010422.

600. Shepherd V, Wood F, Hood K. Establishing a set of research priorities in care homes for older people in the UK: a modified Delphi consensus study with care home staff. Age Ageing. 2016;46:284-90.

601. Sherifali D, Meneilly G. Diabetes management and education in older adults: the development of a national consensus of key research priorities. Can J Diabetes. 2016;40:31-4.

602. Siebrits R, Winter K, Barnes J, Dent M, Ginster M, Harrison J, et al. Priority water research questions for South Africa developed through participatory processes. Water SA. 2014;40:199.

603. Siegfried AL, Carbone EG, Meit MB, Kennedy MJ, Yusuf H, Kahn EB. Identifying and prioritizing information needs and research priorities of public health emergency preparedness and response practitioners. Disaster Med Public Health Prep. 2017;11:552-61.

604. Sigurdardottir KR, Haugen DF, Rijt CCD van der, Sjøgren P, Harding R, Higginson IJ, et al. Clinical priorities, barriers and solutions in end-of-life cancer care research across Europe. Report from a workshop. Eur J Cancer. 2010;46:1815-22.

605. Simacek KF, Nelson T, Miller-Baldi M, Bolge SC. Patient engagement in type 2 diabetes mellitus research:

what patients want. Patient Prefer Adherence. 2018;12:595-606.

606. Simpson E, Goyal NK, Dhepyasuwan N, Flaherman VJ, Chung EK, Von Kohorn I, et al. Prioritizing a research agenda: a Delphi study of the better outcomes through research for newborns (BORN) network. Hospital Pediatrics. 2014;4:195-202.

607. Simpson PL, Guthrie J, Butler T. Prison health service directors' views on research priorities and organizational issues in conducting research in prison: outcomes of a national deliberative roundtable. Int J Prison Health. 2017;13:113-23.

608. Simpson RC, Cooper SM, Kirtschig G, Larsen S, Lawton S, McPhee M, et al. Future research priorities for lichen sclerosus - results of a James Lind Alliance priority setting partnership. Br J Dermatol. 2019;180:1236-7.

609. Sinclair M, McCullough JE, Elliott D, Latos-Bielenska A, Braz P, Cavero-Carbonell C, et al. Exploring research priorities of parents who have children with down syndrome, cleft lip with or without cleft palate, congenital heart defects, or spina bifida using ConnectEpeople: a social media coproduction research study. J Med Internet Res. 2019;21:e15847.

610. Sitzia J, Harlow W. Lymphoedema 4: research priorities in lymphoedema care. Br J Nurs. 2002;11:531-41.

611. Sivananthan SN, Chambers LW. A method for identifying research priorities for health systems research on health and aging. Healthc Manage Forum. 2013;26:33-6.

612. Sleep J, Bullock I, Grayson K. Establishing priorities for research in education within one college of nursing and midwifery. Nurse Educ Today. 1995;15:439-45.

613. Smith AB, Chisolm S, Deal A, Spangler A, Quale DZ, Bangs R, et al. Patient-centered prioritization of bladder cancer research: patient engagement in research. Cancer. 2018;124:3136-44.

614. Smith J, Keating L, Flowerdew L, O'Brien R, McIntyre S, Morley R, et al. An emergency medicine research priority setting partnership to establish the top 10 research priorities in emergency medicine. Emerg Med J. 2017;34:454-6.

615. Soanes L, Gibson F, Bayliss J, Hannan J. Establishing nursing research priorities on a paediatric haematology, oncology, immunology and infectious diseases unit: a Delphi survey. Eur J Oncol Nurs. 2000;4:108-17.

616. Soanes L, Gibson F, Hannan J, Bayliss J. Establishing nursing research priorities on a paediatric haematology, oncology, immunology and infectious diseases unit: involving doctors and parents. Eur J Oncol Nurs. 2003;7:110-9.

617. Soeteman M, Potratz J, Nielsen JSA, Willems J, Valla FV, et al. Research priorities in pediatric onco-critical care: an international Delphi consensus study. Intensive Care Med. 2019;45:1681-3.

618. Soma M, Hosoi T, Yaeda J. Exploring high-priority research questions in physical therapy using the Delphi study. J Phys Ther Sci. 2009;21:367-71.

619. Somanadhan S, Nicholson E, Dorris E, Brinkley A, Kennan A, Treacy E, Atif A, Ennis S, McGrath V, Mitchell D, O'Sullivan G. Rare disease research partnership (RAinDRoP): a collaborative approach to identify research priorities for rare diseases in Ireland. HRB Open Research. 2020;3:13.

620. Souza JP, Widmer M, Gülmezoglu AM, Lawrie TA, Adejuyigbe EA, Carroli G, et al. Maternal and perinatal health research priorities beyond 2015: an international survey and prioritization exercise. Reprod Health. 2014;11:61.

621. Sowell RL. Identifying HIV/AIDS research priorities for the next millennium: a Delphi study with nurses in AIDS care. J Assoc Nurses AIDS Care. 2000;11:42-52.

622. Spies LA, Gray J, Opollo J, Mbalinda S. Uganda nursing research agenda: a Delphi study. Int Nurs Rev. 2015;62:180-6.

623. Steele R, Bosma H, Johnston MF, Cadell S, Davies B, Siden H, et al. Research priorities in pediatric palliative care: a Delphi study. J Palliat Care. 2008;24:229-39.

624. Steele SG, Booy R, Mor SM. Establishing research priorities to improve the One Health efficacy of Australian general practitioners and veterinarians with regard to zoonoses: a modified Delphi survey. One Health. 2018;6:7-15.

625. Stefanidis D, Cochran A, Sevdalis N, Mellinger J, Phitayakorn R, Sullivan M, et al. Research priorities for multi-institutional collaborative research in surgical education. Am J Surg. 2015;209:52-8.

626. Stefanidis D, King WC, Puzziferri N, Butler AR, Hutter M, Sudan R. Development of ASMBS research agenda for bariatric surgery using the Delphi methodology. Surg Obes Relat Dis. 2019;15:1563-9.

627. Stefanidis D, Montero P, Urbach DR, Qureshi A, Perry K, Bachman SL, et al. SAGES research agenda in gastrointestinal and endoscopic surgery: updated results of a Delphi study. Surg Endosc. 2014;28:2763-71.

628. Stephens RJ, Whiting C, Cowan K. Research priorities in mesothelioma: a James Lind Alliance priority setting partnership. Lung Cancer. 2015;89:175-80.

629. Stevens KR, Ovretveit J. Improvement research priorities: USA survey and expert consensus. Nurs Res Pract. 2013;2013:1-8.

630. Steward B. The BAHT 2004 R&D prioritisation exercise: results of a study using a nominal group technique to identify priority research topics and research training needs for hand therapists. The British J Hand Ther. 2004;9:128-32.

631. Stewart MK, Archie DS, Marshall SA, Allison MK, Robinson C. Transform health Arkansas: a transgender-led partnership engaging transgender/non-binary Arkansans in defining health research priorities. Prog Community Health Partnersh. 2017;11:427-39.

632. Strand M, Zvrskovec J, Hübel C, Peat CM, Bulik CM, Birgegård A. Identifying research priorities for the study of atypical anorexia nervosa: a Delphi study. Int J Eat Disord. 2020;53:1729-38.

633. Strauss VY, Carter P, Ong BN, Bedson J, Jordan KP, Jinks C, et al. Public priorities for joint pain research: results from a general population survey. Rheumatology. 2012;51:2075-82.

634. Struwe LA, Douglas-Ybarra C, Kingston EM, Laws RP, Song WK, Gillispie GM, et al. Research priorities of VA nurses. Nurs Manag. 2018;49:36-41.

635. Sullivan R, Ugalde A, Sinclair C, Breen LJ. Developing a research agenda for adult palliative care: a modified Delphi study. J Palliat Med. 2019;22:480-8.

636. Sun C, Dohrn J, Klopper H, Malata A, Omoni G, Larson E. Clinical nursing and midwifery research priorities in Eastern and Southern African countries: results from a Delphi survey. Nursing Research. 2015;64:466-75.

637. Sun C, Dohrn J, Oweis A, Huijer HA-S, Abu-Moghli F, Dawani H, et al. Delphi survey of clinical nursing and midwifery research priorities in the Eastern Mediterranean region. J Nurs Scholarsh. 2017;49:223-35.

638. Sun C, Prufeta P. Using a Delphi survey to develop clinical nursing research priorities among nursing management. J Nurs Adm. 2019;49:156-62.

639. Sun CJ, Fu CJ, Altaweli R, Al Touby S, Ghazi C, Guimei M. Research priorities of clinical nurses and midwives in the Eastern Mediterranean region: a mixed methods study. J Epidemiol Glob Health. 2019;9:36.

640. Swedish Agency for Health Technology Assessment and Assessment of Social Services. 2018. Prioritised research areas within the fields of prevention, diagnosis and treatment of maternal birth injuries. https://www.sbu.se/en/publications/Prioiritisation-of-scientific-evidence-gaps/prioritised-research-areas-within-the-fields-of-prevention-diagnosis-and-treatment-of-maternal-birth-injuries/. Accessed 26 July 2021.

641. Swedish Agency for Health Technology Assessment and Assessment of Social Services. 2019. Priorities for research on social services: perspectives from users, policy-makers and practitioners. https://www.sbu.se/en/publications/Prioiritisation-of-scientific-evidence-gaps/priorities-for-research-on-social-services/. Accessed 26 July 2021.

642. Swedish Agency for Health Technology Assessment and Assessment of Social Services. 2019. Prioritisation of research questions about maternal birth injuries. https://www.sbu.se/en/publications/Prioiritisation-of-scientific-evidence-gaps/prioritisation-of-research-questions-about-maternal-birth-injuries/. Accessed 26 July 2021.

643. Synnot A, Bragge P, Lowe D, Nunn JS, O'Sullivan M, Horvat L, et al. Research priorities in health communication and participation: international survey of consumers and other stakeholders. BMJ Open. 2018;8:e019481.

644. Synnott G, McKie D. International issues in PR: researching research and prioritizing priorities. Journal of Public Relations Research. 1997;9:259-82.

645. Tan NC, Ng CJ, Rosemary M, Wahid K, Goh LG. Developing a primary care research agenda through collaborative efforts - a proposed “6E” model. Asia Pac Fam Med. 2014;13:17.

646. Tao L, Fedoruk C, Turner KA, Cumin J, Carrier M-E, Carboni-Jiménez A, et al. The Scleroderma research topics survey for patients and health care professionals: a Scleroderma patient-centered intervention network project. J Scleroderma Relat Disord. 2019;4:165-72.

647. Taryana AA, Krishnasamy R, Bohm C, Palmer SC, Wiebe N, Boudville N, et al. Physical activity for people with chronic kidney disease: an international survey of nephrologist practice patterns and research priorities. BMJ Open. 2019;9:e032322.

648. Tatham KC, McAuley DF, Borthwick M, Henderson NG, Bashevoy G, Brett SJ. The National Institute for Health Research critical care research priority setting survey 2018. J Intensive Care Soc. 2020;21:198-201.

649. Taylor CJ, Huntley AL, Burden J, Gadoud A, Gronlund T, Jones NR, et al. Research priorities in advanced heart failure: James Lind alliance priority setting partnership. Open Heart. 2020;7:e001258.

650. The African Peri-Operative Research Group (APORG) Working Group 2020: Priorities for peri-operative research in Africa. Anaesthesia. 2020;75:e28-33.

651. Thomas KS, Brindle R, Chalmers JR, Gamble B, Francis NA, Hardy D, et al. Identifying priority areas for research into the diagnosis, treatment and prevention of cellulitis (erysipelas): results of a James Lind Alliance priority setting partnership. Br J Dermatol. 2017;177:541-3.

652. Thomas RH, Hammond CL, Bodger OG, Rees MI, Smith PEM. Identifying and prioritising epilepsy treatment uncertainties. J Neurol Neurosurg Psychiatry. 2010;81:918-21.

653. Thompson W, Reeve E, Moriarty F, Maclure M, Turner J, Steinman MA, et al. Deprescribing: future directions for research. Res Social Adm Pharm. 2019;15:801-5.

654. Thumboo J, Yoon S, Wee S, Yeam CT, Low ECT, Lee CE. Developing population health research priorities in Asian city state: results from a multi-step participatory community engagement. PLoS ONE. 2019;14:e0216303.

655. Tiernan J, Cook A, Geh I, George B, Magill L, Northover J, et al. Use of a modified Delphi approach to develop research priorities for the Association of Coloproctology of Great Britain and Ireland. Colorectal Dis. 2014;16:965-70.

656. Tikellis G, Tong A, Lee JY, Corte TJ, Hey-Cunningham AJ, Bartlett M, Crawford T, Glaspole I, Price J, Maloney J, Holland AE. Top 10 research priorities for people living with pulmonary fibrosis, their caregivers, healthcare professionals and researchers. Thorax. 2021;76:575-81.

657. Tomlinson M, Chopra M, Sanders D, Bradshaw D, Hendricks M, Greenfield D, et al. Setting priorities in child health research investments for South Africa. PLoS Med. 2007;4:e259.

658. Tomlinson M, Darmstadt GL, Yousafzai AK, Daelmans B, Britto P, Gordon SL, et al. Global research priorities to accelerate programming to improve early childhood development in the sustainable development era: a CHNRI exercise. J Glob Health. 2019;9:020703.

659. Tomlinson M, Jordans M, MacMillan H, Betancourt T, Hunt X, Mikton C. Research priority setting for integrated early child development and violence prevention (ECD+) in low and middle income countries: an expert opinion exercise. Child Abuse Neglect. 2017;72:131-9.

660. Tomlinson M, Swartz L, Officer A, Chan KY, Rudan I, Saxena S. Research priorities for health of people with disabilities: an expert opinion exercise. Lancet. 2009;374:1857-62.

661. Tomlinson M, Yasamy MT, Emerson E, Officer A, Richler D, Saxena S. Setting global research priorities for developmental disabilities, including intellectual disabilities and autism: Setting research priorities for developmental disabilities. J Intellect Disabil Res. 2014;58:1121-30.

662. Tomlinson M. Setting priorities for global mental health research. Bull World Health Org. 2009;87:438-46.

663. Tong A, Crowe S, Chando S, Cass A, Chadban SJ, Chapman JR, et al. Research priorities in CKD: report of a national workshop conducted in Australia. Am J Kidney Dis. 2015;66:212-22.

664. Tong A, Sainsbury P, Carter SM, Hall B, Harris DC, Walker RG, et al. Patients' priorities for health research: focus group study of patients with chronic kidney disease. Nephrol Dial Transplant. 2008;23:3206-14.

665. Trezzini B, Phillips B. Impact of legal regulations and administrative policies on persons with SCI: identifying potential research priorities through expert interviews. Disabil Rehabil. 2014;36:1883-91.

666. Tuffrey-Wijne I, Wicki M, Heslop P, McCarron M, Todd S, Oliver D, et al. Developing research priorities for palliative care of people with intellectual disabilities in Europe: a consultation process using nominal group technique. BMC Palliat Care. 2016;15:36.

667. Tume LN, van den Hoogen A, Wielenga JM, Latour JM. An electronic Delphi study to establish pediatric intensive care nursing research priorities in twenty European countries. Pediatr Crit Care Med. 2014;15:e206-13.

668. Tunnicliffe DJ, Singh-Grewal D, Craig JC, Howell M, Tugwell P, Mackie F, et al. Healthcare and research priorities of adolescents and young adults with systemic lupus erythematosus: a mixed-methods study. J Rheumatol. 2017;44:444-51.

669. Turner GM, Backman R, McMullan C, Mathers J, Marshall T, Calvert M. Establishing research priorities relating to the long-term impact of TIA and minor stroke through stakeholder-centred consensus. Res Involv Engagem. 2018;4:2.

670. Turner S, Ollerhead E, Cook A. Identifying research priorities for public health research to address health inequalities: use of Delphi-like survey methods. Health Res Policy Sys. 2017;15:87.

671. Underwood E, Woods M, Riches K, Keeley V, Wallace A, Freeman J. Lymphedema research prioritization partnership: a collaborative approach to setting research priorities for lymphedema managementc. 2019;17:356-61.

672. Uneke CJ, Ezeoha AE, Ndukwe CD, Oyibo PG, Onwe F, Aulakh BK. Research priority setting for health policy and health systems strengthening in Nigeria: the policymakers' and stakeholders' perspective and involvement. Pan Afr Med J. 2013;16. doi:10.11604/pamj.2013.16.10.2318.

673. Urbach DR, Horvath KD, Baxter NN, Jobe BA, Madan AK, Pryor AD, et al. A research agenda for gastrointestinal and endoscopic surgery. Surg Endosc. 2007;21:1518-25.

674. Urcullo G, Muñoz R, Bitrán R. 2008. Identification of priority research questions in LAC within the areas of health financing, human resources for health and the role of the non-state sector final report. https://www.who.int/alliance-hpsr/researchsynthesis/Alliance%20HPSR%20-%20IPRQ%20-%20LAC-%20Bitran.pdf. Accessed 26 July 2021.

675. Uscher-Pines L, Babin SM, Farrell CL, Hsieh Y-H, Moskal MD, Gaydos CA, et al. Research priorities for syndromic surveillance systems response: consensus development using nominal group technique. J Public Health Manag Pract. 2010;16:529-34.

676. Uyei J, Li L, Braithwaite RS. HIV and Alcohol Research Priorities of City, State, and Federal Policymakers: Results of a Delphi Study. Am J Public Health. 2015;105:e23-6.

677. van de Glind I, Berben S, Zeegers F, Poppen H, Hoogeveen M, Bolt I, et al. A national research agenda for pre-hospital emergency medical services in the Netherlands: a Delphi-study. Scand J Trauma Resusc Emerg Med. 2016;24:2.

678. van der Beek AJ, Frings-Dresen MH, van Dijk FJ, Houtman IL. Priorities in occupational health research: a Delphi study in the Netherlands. Occup Environ Med. 1997;54:504-10.

679. van Furth EF, van der Meer A, Cowan K. Top 10 research priorities for eating disorders. Lancet Psychiatry. 2016;3:706-7.

680. van Galen KP, Lavin M, Skouw-Rasmussen N, Ivanova E, Mauser-Bunschoten E, Punt M, Romana G, Elfvinge P, D'Oiron R, Abdul-Kadir R. Clinical management of woman with bleeding disorders: a survey among European haemophilia treatment centres. Haemophilia. 2020;26:657-62.

681. van Hoving DJ, Barnetson BK, Wallis LA. Emergency care research priorities in South Africap. S Afr Med J. 2015;105:202.

682. van Merode T, Bours S, van Steenkiste B, Sijbers T, van der Hoek G, Vos C, et al. Describing patients' needs in the context of research priorities in patients with multiple myeloma or Waldenstrom's disease: a

truly patient-driven study. Z Evid Fortbild Qual Gesundhwes. 2016;112:11-8.

683. van Middendorp JJ, Allison HC, Ahuja S, Bracher D, Dyson C, Fairbank J, et al. Top ten research priorities for spinal cord injury: the methodology and results of a British priority setting partnership. Spinal Cord. 2016;54:341-6.

684. van Schalkwyk SC, Kiguli-Malwadde E, Budak JZ, Reid MJA, de Villiers MR. Identifying research priorities for health professions education research in sub-Saharan Africa using a modified Delphi method. BMC Med Educ. 2020;20:443.

685. Vasa RA, Keefer A, Reaven J, South M, White SW. Priorities for advancing research on youth with autism spectrum disorder and co-occurring anxiety. J Autism Dev Disord. 2018;48:925-34.

686. Velayutham B, Nair D, Ramalingam S, Perez-Velez CM, Becerra MC, Swaminathan S. Setting priorities for a research agenda to combat drug-resistant tuberculosis in children. Public Health Action. 2015;5:222.

687. Vella-Baldacchino M, Perry DC, Roposch A, Nicolaou N, Cooke S, Ellis P, et al. Research priorities in children requiring elective surgery for conditions affecting the lower limbs: a James Lind Alliance priority setting partnership. BMJ Open. 2019;9:e033233.

688. Ventura MR, Waligora-Serafin B. Setting priorities for nursing research. J Nurs Adm. 1981;11:30-5.

689. Villares E, Dimmitt C. Updating the school counseling research agenda: a Delphi study. Counselor Education and Supervision. 2017;56:177-92.

690. Vohra S, Zorzela L, Kemper K, Vlieger A, Pintov S. Setting a research agenda for pediatric complementary and integrative medicine: a consensus approach. Complement Ther Med. 2019;42:27-32.

691. Von Ah D, Brown C, Brown S, Bryant A, Davies M, Dodd M, et al. Research agenda of the oncology nursing society: 2019–2022. Oncol Nurs Forum. 2019;46:654-69.

692. von Scheven E, Nahal BK, Cohen IC, Kelekian R, Franck LS. Research questions that matter to us: priorities of young people with chronic illnesses and their caregivers. Pediatr Res. 2020; 17:1-5.

693. Waiswa P, Okuga M, Kabwijamu L, Akuze J, Sengendo H, Aliganyira P, et al. Using research priority-setting to guide bridging the implementation gap in countries - a case study of the Uganda newborn research priorities in the SDG era. Health Res Policy Sys. 2019;17:54.

694. Walker AM. A Delphi study of research priorities in the clinical practice of physiotherapy. Physiotherapy. 1994;80:205-7.

695. Wan YL, Beverley-Stevenson R, Carlisle D, Clarke S, Edmondson RJ, Glover S, et al. Working together to shape the endometrial cancer research agenda: the top ten unanswered research questions. Gynecol Oncol. 2016;143:287-93.

696. Ward DS, Vaughn A, Story M. Expert and stakeholder consensus on priorities for obesity prevention research in early care and education settings. Childhood Obesity. 2013;9:116-24.

697. Wathen CN, MacGregor JC, Hammerton J, Coben JH, Herrman H, et al. Priorities for research in child maltreatment, intimate partner violence and resilience to violence exposures: results of an international Delphi consensus development process. BMC Public Health. 2012;12:684.

698. Watson T. Public relations research priorities: a Delphi study. Journal of Communication Management. 2008;12:104-23.

699. Wazny K, Arora NK, Mohapatra A, Gopalan HS, Das MK, Nair M, et al. Setting priorities in child health research in India for 2016-2025: a CHNRI exercise undertaken by the Indian Council for Medical Research and INCLEN Trust. J Glob Health. 2019;9:020701.

700. Wazny K, Sadruddin S, Zipursky A, Hamer DH, Jacobs T, Kallander K, et al. Setting global research priorities for integrated community case management (iCCM): Results from a CHNRI (Child Health and Nutrition Research Initiative) exercise. J Glob Health. 2014;4. doi:10.7189/jogh.04.020413.

701. Webber GC, Spitzer DL. Sexual and reproductive health issues facing Southeast Asian beer promoters: a qualitative pilot study. BMC Public Health. 2010;10:389.

702. Weeks LC, Seely D, Balneaves LG, Boon HS, Leis A, Oneschuk D, et al. Canadian integrative oncology research priorities: results of a consensus-building process. Curr Oncol. 2013;20:289-99.

703. Weeks R, Adams VM. Research priorities for conservation and natural resource management in Oceania's small-island developing states. Conservation Biology. 2018;32:72-83.

704. Weenen TC, Jentink A, Pronker ES, Commandeur HR, Claassen E, Boirie Y, et al. Patient needs and research priorities in the enteral nutrition market - a quantitative prioritization analysis. Clin Nutr. 2014;33:793-801.

705. Welfare MR, Colligan J, Molyneux S, Pearson P, Barton JR. The identification of topics for research that are important to people with ulcerative colitis. Eur J Gastroenterol Hepatol. 2006;18:939-44.

706. Wielenga JM, Tume LN, Latour JM, van den Hoogen A. European neonatal intensive care nursing research priorities: an e-Delphi study. Arch Dis Child Fetal Neonatal Ed. 2015;100:F66-71.

707. Wiener B, Chacko S, Brown TR, Cron SG, Cohen MZ. Delphi survey of research priorities. J Nurs Manag. 2009;17:532-8.

708. Wilkes L, Cummings J, McKay N. Developing a culture to facilitate research capacity building for clinical nurse consultants in generalist paediatric practice. Nurs Res Pract. 2013;2013:1-8.

709. Willett KM, Gray B, Moran CG, Giannoudis PV, Pallister I. Orthopaedic trauma research priority-setting exercise and development of a research network. Injury. 2010;41:763-7.

710. Williams A, Sell D, Oulton K, Wilson N, Wray J, Gibson F. Identifying research priorities with nurses at a tertiary children's hospital in the United Kingdom. Child Care Health Dev. 2017;43:211-21.

711. Williams MT, Lewthwaite H, Brooks D, Jensen D, Abdallah SJ, Johnston KN. Chronic breathlessness explanations and research priorities: findings from an international Delphi survey. J Pain Symptom Manage. 2020;59:310-319.e12.

712. Wilson MSJ, Blencowe NS, Boyle C, Knight SR, Petty R, Vohra RS, et al. A modified Delphi process to establish future research priorities in malignant oesophagogastric surgery. The Surgeon. 2020;18:321-6.

713. Wilson MSJ, Knight S, Vaughan-Shaw P, Blakemore AI, O'Kane M, Boyle C, et al. A modified AUGIS Delphi process to establish research priorities in bariatric and metabolic surgery. Clin Obes. 2020;10. doi:10.1111/cob.12344.

714. Wilson MSJ, Vaughan-Shaw P, Boyle C, Yong GL, Oglesby S, et al. A modified AUGIS Delphi process to establish future research priorities in benign upper gastrointestinal surgery. World J Surg. 2020;44:1216-22.

715. Wilson P, Gurusamy KS, Morley R, Whiting C, Maeso B, FitzGerald G, et al. Top research priorities in healthcare-associated infection in the UK. J Hosp Infect. 2019;103:382-7.

716. Wilson S, Ramelet A-S, Zuiderduyn S. Research priorities for nursing care of infants, children and adolescents: a West Australian Delphi study. J Clin Nurs. 2010;19:1919-28.

717. Wong CHL, Wu IXY, Balneaves LG, Lo RSK, Witt CM, Wu JCY, et al. Prioritizing Chinese medicine clinical research questions in cancer palliative care: international Delphi survey. J Pain Symptom Manage. 2019;58:1002-1014.e7.

718. Woodward A, Sondorp E, Witter S, Martineau T. Health systems research in fragile and conflict-affected states: a research agenda-setting exercise. Health Res Policy Sys. 2016;14:51.

719. Wright M-O, Carter E, Pogorzelska M, Murphy C, Hanchett M, Stone PW. The APIC research agenda: results from a national survey. Am J Infect Control. 2012;40:309-13.

720. Wright TSA. Developing research priorities with a cohort of higher education for sustainability experts. Int J of Sus in Higher Ed. 2007;8:34-43.

721. Wynaden D, Heslop K, Al Omari O, Nelson D, Osmond B, Taylor M, et al. Identifying mental health nursing research priorities: a Delphi study. Contemp Nurse. 2014;47:16-26.

722. Yan A, Millon-Underwood S, Walker A, Patten C, Nevels D, Dookeran K, et al. Engaging young African American women breast cancer survivors: a novel storytelling approach to identify patient-centred research priorities. Health Expect. 2020;23:473-82.

723. Yin TJC, Hsu N, Tsai SL, Wang BW, Shaw FL, Shih FJ, et al. Priority-setting for nursing research in the Republic of China. J Adv Nurs. 2000;32:19-27.

724. Yong GL, Weir-McCall J, Wilson M, Roditi GH, Bull R, Williams MC, et al. Research priorities in cardiovascular imaging. Open Heart. 2020;7:e001389.

725. Young AF, Chesson RA. Determining research questions on health risks by people with learning disabilities, carers and care-workers. Br J Learning Disab. 2008;36:22-31.

726. Zeigler VL, Decker-Walters B. Determining psychosocial research priorities for adolescents with implantable cardioverter defibrillators using Delphi methodology. J Cardiovasc Nurs. 2010;25:398-404.

727. Zeitlin J, Sentenac M, Morgan AS, Ancel PY, Barros H, Cuttini M, et al. Priorities for collaborative research using very preterm birth cohorts. Arch Dis Child Fetal Neonatal Ed. 2020;105:538-44.

728. Zimmerman EB, Cook SK, Haley AD, Woolf SH, Price SK. A patient and provider research agenda on diabetes and hypertension management. Am J Prev Med. 2017;53:123-9.

729. Zipursky A, Wazny K, Black R, Keenan W, Duggan C, Olness K, et al. Global action plan for childhood diarrhoea: developing research priorities. J Glob Health. 2013;3. doi:10.7189/jogh.03.010406.

730. Zitko P, Borghero F, Zavala C, Markkula N, Santelices E, Libuy N, et al. Priority setting for mental health research in Chile. Int J Ment Health Syst. 2017;11:61.

731. Zugcic M, Davis JE, Gorski LA, Alexander M. Establishing research priorities for the Infusion Nurses Society. J Infus Nurs. 2010;33:176-82.
